# Captivating Synergistic, Dose-Dependent Anticancer Effects of Tumor-Regulation Modulators Chloroquine and Ivermectin Completely Abolished by an Opposing Modulator, Deoxycholic Acid, in Hamster Fibrosarcoma: In Vivo, In Vitro, and Literature Review

**DOI:** 10.3390/ph19030407

**Published:** 2026-03-01

**Authors:** Kosta J. Popović, Dušica J. Popović, Dejan Miljković, Jovan K. Popović, Mihalj Poša, Jovana Drljača Lero, Zana Dolićanin

**Affiliations:** 1 Department of Pharmacy, Faculty of Medicine, University of Novi Sad, Hajduk Veljkova 3, 21000 Novi Sad, Serbia; kosta.popovic@mf.uns.ac.rs (K.J.P.); mihalj.posa@mf.uns.ac.rs (M.P.); jovana.drljaca-lero@mf.uns.ac.rs (J.D.L.); 2 Department of Biomedical Sciences, State University of Novi Pazar, Vuka Karadžića 9, 36300 Novi Pazar, Serbia; dusicapopovic@np.ac.rs (D.J.P.); zdolicanin@np.ac.rs (Z.D.); 3 Department of Histology and Embryology, Faculty of Medicine, University of Novi Sad, Hajduk Veljkova 3, 21000 Novi Sad, Serbia; dejan.miljkovic@mf.uns.ac.rs; 4 Department of Pharmacology, Toxicology and Clinical Pharmacology, Faculty of Medicine, University of Novi Sad, Hajduk Veljkova 3, 21000 Novi Sad, Serbia; 5 Academy of Medical Sciences of the Serbian Medical Society, 19 George Washington Str., 11000 Belgrade, Serbia

**Keywords:** chloroquine, ivermectin, deoxycholic acid, hamsters, fibrosarcoma, anticancer synergism

## Abstract

**Background/Objectives**: In previous studies, chloroquine and ivermectin separately exhibited similar anticancer effects on various known cancer modulatory targets. This study aimed (1) to identify a non-toxic synergistic combination of chloroquine and ivermectin that suppresses hamster fibrosarcoma; (2) to verify combined antitumor efficacy using dose–response analysis; and (3) to investigate potential synergistic mechanisms by restoring tumor progression with the reciprocal cancer-modulating agent deoxycholic acid. **Methods**: A BHK-21/C13 cell culture was subcutaneously inoculated into Syrian golden hamsters randomly divided into groups (6 animals per group): (1) untreated control; treated daily (17 days after inoculation) with (2) chloroquine 50 mg/kg; (3) ivermectin 5 mg/kg; (4) a combination of chloroquine 50 mg/kg and ivermectin 5 mg/kg; (5) a combination of chloroquine 50 mg/kg, ivermectin 5 mg/kg and deoxycholic acid 100 mg/kg; (6) a combination of chloroquine 25 mg/kg and ivermectin 2.5 mg/kg; (7) a combination of chloroquine 12.5 mg/kg and ivermectin 1.25 mg/kg. Dose–response curves were generated for chloroquine and ivermectin combinations. Characteristics of tumors (growth kinetics, biophysical, histological, immunohistochemical, pathological), hamster organs, biochemical and hematological blood tests were compared among the groups. **Results**: The synergistic, dose-dependent anticancer effects of two antiparasitic agents, similar tumor-regulation modulators chloroquine and ivermectin, in doses equivalent to human doses were observed in fibrosarcoma in hamsters (both drugs approximately 1/10 LD_50_) without toxicity and in various cell lines of human lung, colon and cervical carcinomas and hamster fibrosarcoma in vitro. The addition of a reciprocal modulator of cancer regulation, NF-κB stimulator deoxycholic acid, caused a huge rescue effect on fibrosarcoma and a reversal of the successful anticancer therapy using the combination. **Conclusions**: The chloroquine and ivermectin combination may be recommended for comprehensive additional preclinical and clinical evaluation due to its synergistic anticancer effects. Further preclinical and clinical exploration will be crucial to thoroughly define the optimal role of the combination therapy in the treatment of fibrosarcoma and potentially other cancer types.

## 1. Introduction

The development of novel therapeutics represents a prolonged, intricate, and costly endeavor. Evaluating the anticancer potential of pleiotropic compounds, which are already approved for other clinical indications, as well as their combinatorial effects, can substantially shorten the duration and reduce the financial burden of preclinical and clinical investigations. Antiparasitic agents, pointedly chloroquine and ivermectin, are among those that have various properties and mechanisms that may affect various diseases, including malignancies [1]. Before our experiment, we conducted a literature review, which indicated that chloroquine and ivermectin very likely have anticancer interactions. Furthermore, our literature review revealed so much data in favor of chloroquine and ivermectin as common anticancer targets that we summarized it all in Table 1 and Supplemental Table S1. In published studies on different malignancies (a few on normal cells, predominantly in vitro and rarely on xenograft animal models), chloroquine and ivermectin separately exhibited similar anticancer effects on various known cancer modulatory targets (Table 1 and Supplemental Table S1). Both drugs, chloroquine and ivermectin, are documented to modulate the same anticancer cell processes by a yet elusive precise mode of action. Combining chemically different drugs that affect the same anticancer targets/pathways allows the therapeutic doses of each drug to be applied with a stronger effect without enhancing the side effects and toxicity for each drug. Since these two multitargeted antiparasitic agents share the same key antitumor mechanisms, it can be postulated that a combination of these drugs may be a promising synergistic anticancer protocol. The aims of this study include (1) to discover a synergistic, non-toxic anticancer chloroquine and ivermectin combination that suppresses fibrosarcoma in hamsters and could be recommended for further preclinical and clinical investigations and have potential for future application in oncology; (2) to validate the combinatory anticancer effects by a dose–response relationship; and (3) To discover possible underlying synergistic anticancer mechanisms of a chloroquine and ivermectin combination by rescuing tumor progression with the reciprocal cancer modulatory agent deoxycholic acid. The enormous number of common anticancer targets is the reason for planning simultaneous dose–response and rescue experiments. Based on the already known anticancer mechanisms of chloroquine and ivermectin (Table 1 and Supplemental Table S1), we designed and conducted a rescue experiment with the opposite cancer growth modulator deoxycholic acid, which acts in the pro-cancer direction (Table 1 and Supplemental Table S1).

Considering that we have previous experience with a hamster fibrosarcoma experimental model in vivo and to the best of our knowledge the combination of chloroquine and ivermectin has not previously been reported as anticancer therapy, here we tested that hypothesis on fibrosarcoma in hamsters. We showed that chloroquine has synergistic interaction with ivermectin in hamster fibrosarcoma and that in rescue experiments, deoxycholic acid can be used.

## 2. Results

All animals, both control and treated, survived throughout the entire duration of the experiment.

### 2.1. Biophysical Evaluation of Hamster Fibrosarcoma Characteristics

**Figure 1:**
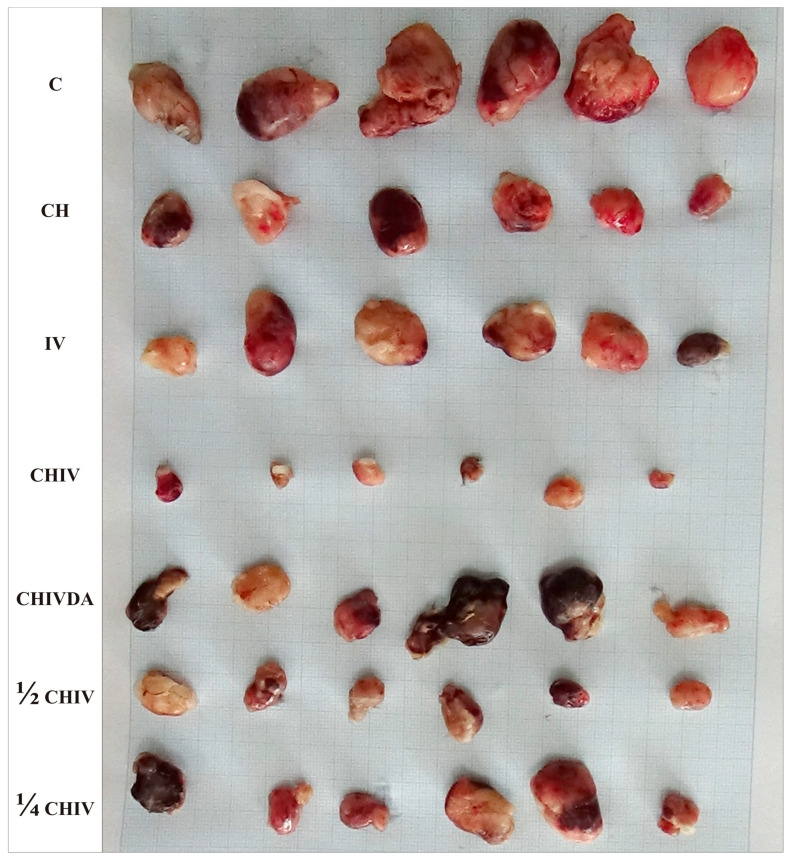
Extirpated fibrosarcomas placed on one-millimeter grid paper for visual dimension comparison: C—control group; CH—group treated with chloroquine; IV—group treated with ivermectin; CHIV—group treated with the combination of chloroquine and ivermectin; CHIVDA—group treated with the combination of chloroquine, ivermectin and deoxycholic acid; ½CHIV—group treated with 1/2 doses of chloroquine and ivermectin used in CHIV group; ¼CHIV—group treated with 1/4 doses of chloroquine and ivermectin used in CHIV group.

At the conclusion of the treatments, 17 days following BHK-21/C13 inoculation, all hamsters across all groups exhibited well-demarcated, solid tumors at the injection site (Figure 1). No adverse effects on general health or well-being were observed, and no pathological or histopathological signs of toxicity were noted in the major organs (heart, lungs, stomach, intestine, liver, kidneys, and brain), nor were metastases or ascites detected.

**Figure 2:**
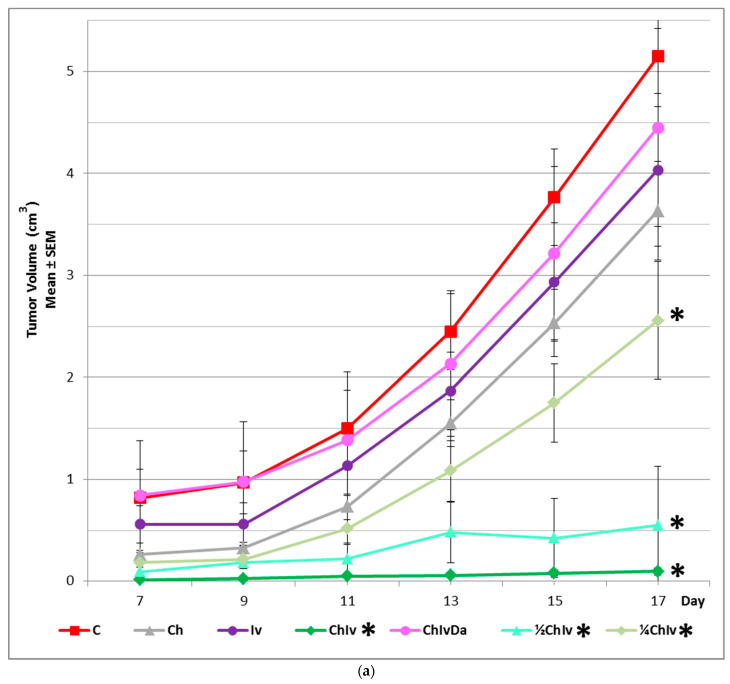

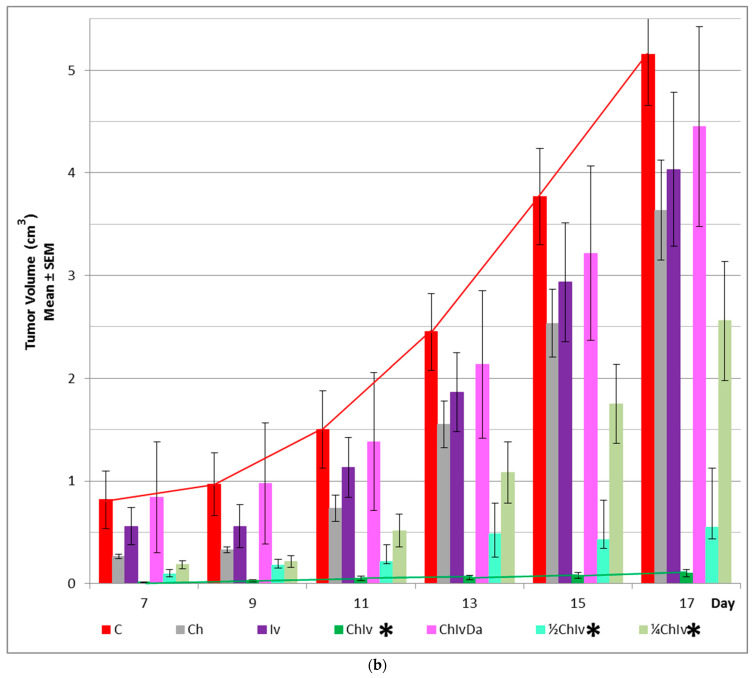
(**a**) Tumor volume growth during the experiment: interpolated line between means and SEM values. C—control group; Ch—group treated with chloroquine; Iv—group treated with ivermectin; ChIv—group treated with the combination of chloroquine and ivermectin; ChIvDa—group treated with the combination of chloroquine, ivermectin and deoxycholic acid; ½ChIv—group treated with 1/2 doses of chloroquine and ivermectin; ¼ChIv—group treated with 1/4 doses of chloroquine and ivermectin. * statistically significant compared to control. * *p* < 0.05, as indicated. (**b**) Tumor volume growth during course of the experiment: column chart with separated SEM values and interpolated lines between average values for groups that showed maximum (green line) and minimum (red line) response to therapy. C—control group; Ch—group treated with chloroquine; Iv—group treated with ivermectin; ChIv—group treated with the combination of chloroquine and ivermectin; ChIvDa—group treated with the combination of chloroquine, ivermectin and deoxycholic acid; ½ChIv—group treated with 1/2 doses of chloroquine and ivermectin; ¼ChIv—group treated with 1/4 doses of chloroquine and ivermectin. * statistically significant compared to control. * *p* < 0.05, as indicated.

In the experiment, detectable tumor formation commenced approximately 7 days post-inoculation (Figure 2a,b), with the maximum tumor diameter reaching < 3.5 cm and the tumor burden remaining below 6%, in accordance with internationally recognized standards (Supplementary Data).

In the experiment, only the co-treatment with chloroquine and ivermectin significantly inhibited tumor growth (*p* < 0.05), as evidenced by marked reductions in tumor weight, maximum length, volume, burden (relative tumor to hamster weight), density, surface area, and the ratios of maximum length/density, area/density, and volume/density, when compared to the control, one-drug treatments, and the three-drug treatment (Table 2 and Table 3).

### 2.2. Immunohistochemical Evaluation of Hamster Fibrosarcoma Characteristics

The selection of specific markers for immunohistochemical staining is based on the following rationale: p53 is utilized to assess mutational status, while Ki-67 and PCNA are cellular markers indicative of proliferation. CD34, CD31, GLUT1, iNOS, COX4 and Cytochrome C combined with caspase 3 are employed to evaluate vasculature, glucose metabolism, nitric oxide (NO) production, and apoptosis in tumor specimens, respectively. To validate and corroborate key tumor growth processes, two markers (Ki-67 and PCNA) are used to assess tumor proliferation, while CD34 and CD31 are employed to evaluate tumor angiogenesis. Additionally, three markers (COX4 and Cytochrome C combined with caspase 3) are utilized to examine apoptosis in the tumor. COX4 changes were considered indicative of mitochondrial alterations that may accompany apoptotic processes, rather than being definitive evidence of apoptosis. Apoptosis was primarily inferred from cleaved caspase 3 activation and cytochrome C staining patterns.

**Figure 3:**
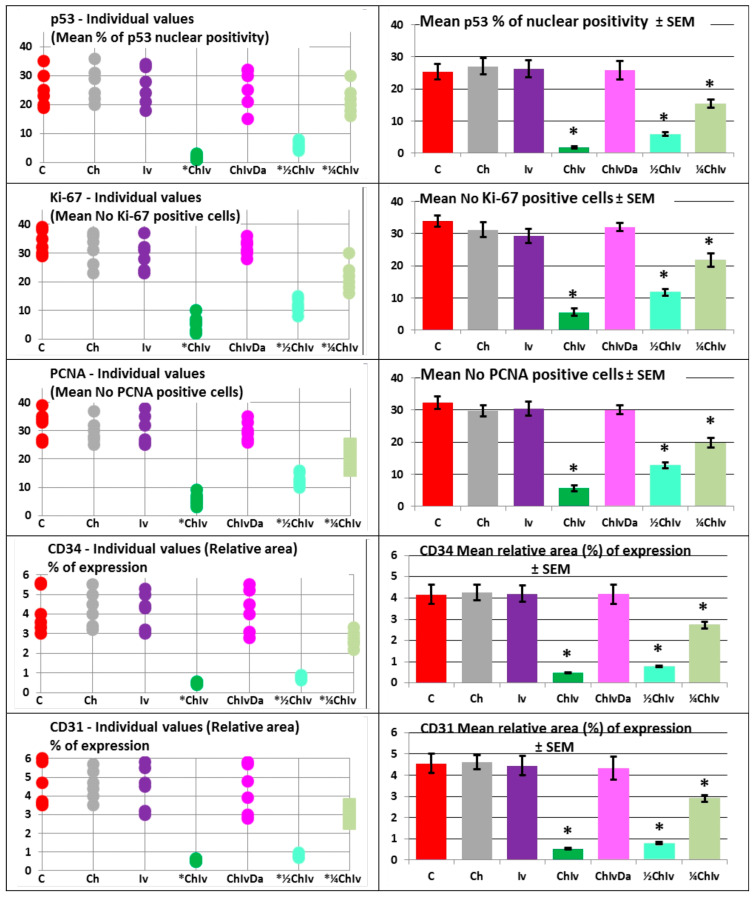
Individual values, means and standard errors (SEM) of immunohistochemical–histopathological characteristics of the excised tumors in the experiment: p53, Ki-67, PCNA, CD34, CD31. C—control group; Ch—group treated with chloroquine; Iv—group treated with ivermectin; ChIv—group treated with the combination of chloroquine and ivermectin; ChIvDa—group treated with the combination of chloroquine, ivermectin and deoxycholic acid; ½ChIv—group treated with 1/2 doses of chloroquine and ivermectin; ¼ChIv—group treated with 1/4 doses of chloroquine and ivermectin. * *p* < 0.05, as indicated.

**Figure 4:**
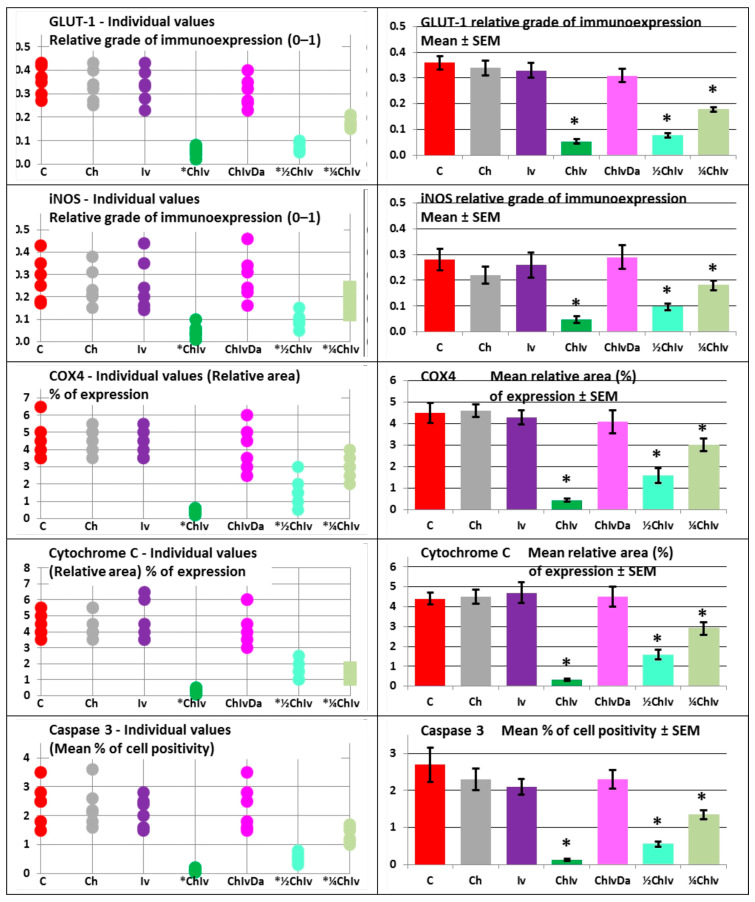
Individual values, means and standard errors (SEM) of immunohistochemical–histopathological characteristics of the excised tumors in the experiment: GLUT-1, iNOS, COX4, Cytochrome C, Caspase 3. C—control group; Ch—group treated with chloroquine; Iv—group treated with ivermectin; ChIv—group treated with the combination of chloroquine and ivermectin; ChIvDa—group treated with the combination of chloroquine, ivermectin and deoxycholic acid; ½ChIv—group treated with 1/2 doses of chloroquine and ivermectin; ¼ChIv—group treated with 1/4 doses of chloroquine and ivermectin. * *p* < 0.05, as indicated.

The pathohistological and immunohistochemical analysis of all tumor slices in the experiment confirmed the biophysical findings and revealed a reduction in tissue penetration, an expansion of necrotic and hemorrhagic areas, and a significant (*p* < 0.05) decrease in mutation status as indicated by p53. Additionally, there was a significant (*p* < 0.05) reduction in tumor cell proliferation, as demonstrated by Ki-67 and PCNA, a significant (*p* < 0.05) inhibition of tumor vasculature, as shown by CD34 and CD31, and a significant (*p* < 0.05) suppression of glucose metabolism, as evidenced by GLUT1. The results also showed significant (*p* < 0.05) inhibition of nitric oxide (NO) production, as indicated by iNOS staining, and a significant (*p* < 0.05) increase in apoptosis intensity, as evidenced by COX4 and Cytochrome C combined with caspase 3, in animals treated with the chloroquine and ivermectin combination, when compared to the control group, single treatments, and the triple treatment (Figure 3 and Figure 4, Table 4).

In the experiment, neither single treatment with chloroquine nor ivermectin demonstrated a significant anticancer effect (*p* > 0.05) when compared to the control group, as evidenced by all analyzed biophysical and immunohistochemical parameters (Figure 1, Figure 2a,b, Figure 3 and Figure 4, Table 2, Table 3 and Table 4). No significant (*p* > 0.05) differences in anticancer activities were observed between the chloroquine and ivermectin treatments (Figure 1, Figure 2a,b, Figure 3 and Figure 4, Table 2, Table 3 and Table 4).

The combined therapy of deoxycholic acid, chloroquine, and ivermectin does not affect sarcoma growth when compared to the control group (*p* > 0.05). In contrast, significant anticancer effects were observed with the chloroquine and ivermectin combination compared to the control (*p* < 0.05) (Figure 1, Figure 2a,b, Figure 3 and Figure 4, Table 2, Table 3 and Table 4).

The treatments had no significant effect (*p* > 0.05) on body weight, red and white blood cell counts, platelet numbers, hemoglobin levels, hematocrit levels, glucose levels, serum proteins, or sedimentation rate when compared to the control group in the experiment (Supplemental Table S2).

In the experiment, co-treatment with the NF-κB stimulator deoxycholic acid completely abolished the combined antitumor effects of the two NF-κB inhibitors, chloroquine and ivermectin. The cancer progression inhibition observed with the chloroquine and ivermectin combination was fully reversed by deoxycholic acid (Figure 1, Figure 2a,b, Figure 3 and Figure 4, Table 2, Table 3 and Table 4).

These results indicate that the significant synergistic anticancer effects observed with co-treatment of the two NF-κB inhibitors, chloroquine and ivermectin (at doses equivalent to or lower than the typical human doses), can be partly attributed to the synergistic inhibition of NF-κB in hamster fibrosarcoma cells.

All findings derived from parametric statistical analysis were further validated using the two-sided non-parametric Mann–Whitney U test.

### 2.3. Combination Index Analysis

Since drug concentrations were not measured, the original form of the Combination Index (CI) calculation [200] was modified by using drug doses instead of concentrations, assuming linear kinetics for the drugs (where doses correlate linearly with concentrations in animal body fluids). Given that the single drug treatments in the experiment were far less effective than their combination, the CI for all measured effects of the chloroquine and ivermectin combination remained < 1, indicating a synergistic anticancer effect.

In the experiment, the synergistic antitumor effects of the chloroquine and ivermectin combination, which were antagonized by deoxycholic acid, were observed in hamster fibrosarcoma, with CI > 1 for all analyzed tumor characteristics.

### 2.4. Dose–Response Experiment

**Figure 5:**
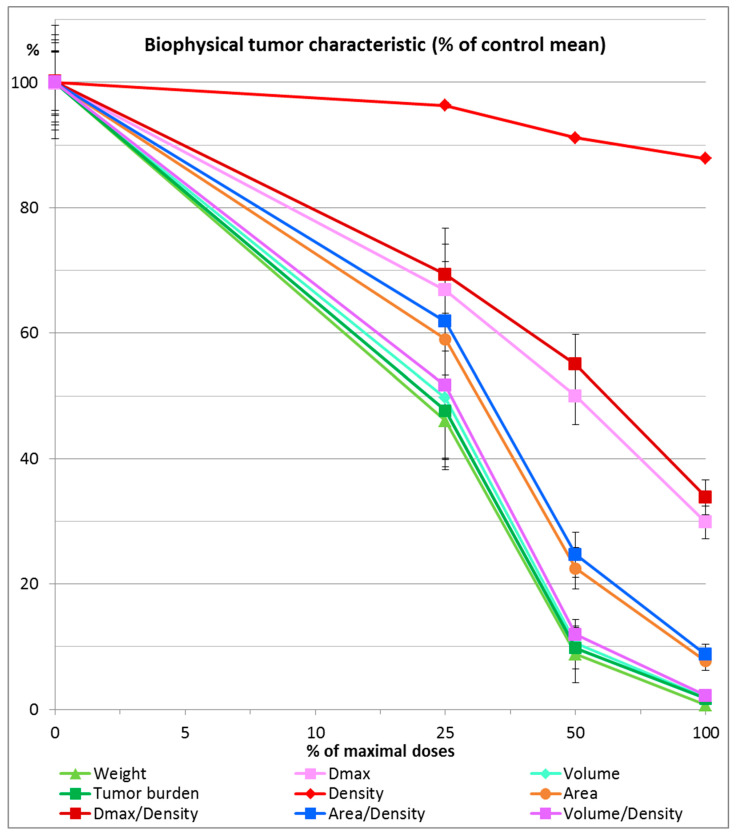
Dose–response curves for biophysical tumor characteristics: weight, maximal diameter (D_max_), volume, tumor burden, tumor density, tumor surface area, tumor D_max_/density ratio, tumor area/density ratio, tumor volume/density ratio for groups in the experiment, expressed as % of control mean (mean ± SEM): 0—control group; 25—group treated with 25% of maximal doses; 50—group treated with 50% of maximal doses; 100—group treated with 100% of maximal doses of chloroquine and ivermectin combination.

**Figure 6:**
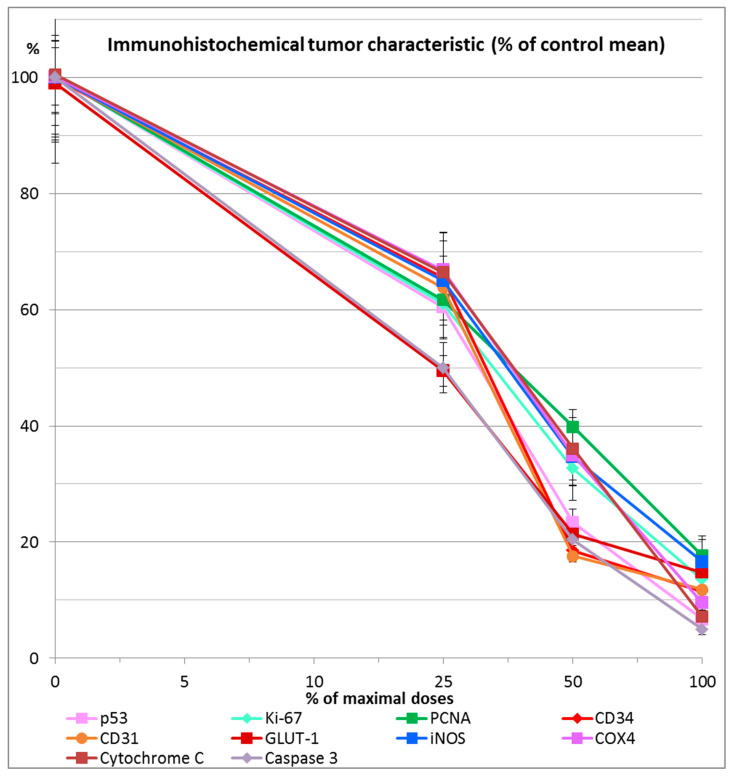
Dose–response curves for immunohistochemical tumor characteristics: p53, Ki-67, PCNA, CD34, CD31, GLUT-1, iNOS, COX4, Cytochrome C and Caspase 3 for groups in the experiment, expressed as % of control mean (mean ± SEM): 0—control group; 25—group treated with 25% of maximal doses; 50—group treated with 50% of maximal doses; 100—group treated with 100% of maximal doses of chloroquine and ivermectin combination.

All examined combinations of chloroquine and ivermectin, namely combination of chloroquine 50 mg/kg and ivermectin 5 mg/kg (CHIV); combination of chloroquine 25 mg/kg and ivermectin 2.5 mg/kg (½CHIV); and combination of chloroquine 12.5 mg/kg and ivermectin 1.25 mg/kg (¼CHIV) exhibited significant anticancer effects (*p* < 0.05) in comparison to control. Furthermore, groups treated with lower doses of chloroquine and ivermectin components in combination (½CHIV = 25 mg/kgCH + 2.5 mg/kgIV; ¼CHIV = 12.5 mg/kgCH + 1.25 mg/kgIV) in comparison to basic CHIV group treated with combination of chloroquine 50 mg/kg and ivermectin 5 mg/kg (100% maximal used dose for both drugs) and also compared with each other (½CHIV/¼CHIV) exhibited significant anticancer effects (*p* < 0.05) with respect to all analyzed biophysical (Figure 5, Table 3) and immunohistochemical (p53, Ki-67, PCNA, CD34, CD31, GLUT-1, iNOS, COX4, Cytochrome C, caspase 3) parameters (Figure 6, Table 4), without effect on general state, toxicity on main organs, blood hematological and biochemical tests. Results of our dose–response experiment show that combination of chloroquine and ivermectin exhibits anticancer effects in a dose-dependent manner (Figure 5 and Figure 6, Table 3 and Table 4).

### 2.5. Co-Treatment with Chloroquine and Ivermectin Antiproliferative Effects in Cancer Cell Lines

Co-treatment with chloroquine and ivermectin exerts antiproliferative effects in cancer cell lines. The antiproliferative effects, expressed as IC_50_, in fibrosarcoma, carcinoma, and normal cell lines for all treatments are presented in Table 5. Co-treatment with chloroquine and ivermectin showed selective cytotoxicity: selectivity index (SI) > 1 against all tested malignant cell lines (BHK-21/C13, A549, HT-29, and HeLa). This combination also demonstrated favorable (non-toxic) antiproliferative effects in normal fetal lung MRC-5 cells, suggesting that it may be both safe and effective.

## 3. Discussion

Effective anticancer therapies frequently target key signaling pathways involved in tumorigenesis, including NF-κB signaling, autophagy, and apoptosis. In line with this paradigm, the therapeutic strategy investigated in the present study combines chloroquine and ivermectin. As summarized in Table 1 and Supplemental Table S1, a substantial proportion of targets and markers modulated by chloroquine and ivermectin in the same direction, and by deoxycholic acid in the opposite direction, are functionally linked to NF-κB signaling, either upstream, downstream, or through direct regulatory interactions.

The experimental model used in this study was selected to minimize host immune rejection and thereby reduce immune-related confounding factors. Consequently, immune-mediated mechanisms within the tumor microenvironment cannot be directly assessed under these conditions.

Nevertheless, NF-κB signaling is not limited to host immune cells and may also operate intrinsically within tumor cells, where it regulates survival, proliferation, angiogenesis, and metabolic pathways. Therefore, references to NF-κB-driven tumor regulation in the present study should be interpreted primarily in the context of tumor-intrinsic signaling rather than host immune modulation.

Further investigations using immunocompetent models would be required to clarify the contribution of immune/inflammatory axes.

The regulatory interplay among targets and markers relevant to cancer development is inherently complex. Individual targets and markers may reciprocally stimulate or inhibit one another and, depending on the cellular context, exert either pro-oncogenic or anti-oncogenic effects. NF-κB and autophagy engage in reciprocal cross-talk, with NF-κB generally promoting tumorigenic processes, while autophagy exerts context-dependent tumor-suppressive or tumor-promoting functions. Similarly, bidirectional regulatory interactions exist between autophagy and apoptosis, as well as between NF-κB and p53.

Moreover, cross-regulatory networks involving NF-κB, p53, autophagy, and both caspase-dependent and caspase-independent apoptotic pathways intersect with multiple additional signaling cascades implicated in the regulation of tumorigenesis. Within this framework, the pronounced, dose-dependent anticancer synergism observed between chloroquine and ivermectin may result from the concurrent modulation of multiple, partially overlapping regulatory checkpoints. The antagonistic modulation observed following tumor rescue treatment with added NF-κB inhibitor deoxycholic acid further supports the involvement of the NF-κB signaling axis in mediating these effects.

Taken together, these findings suggest that NF-κB functions as a central molecular node integrating signals from chloroquine- and ivermectin-modulated pathways. Consistent with our experimental observations and the analysis of relevant literature (Table 1 and Supplemental Table S1) addressing the effects of these agents on shared molecular targets, NF-κB emerges as a pivotal mediator of the synergistic anticancer activity of chloroquine and ivermectin.

The potential synergistic anticancer effects of ivermectin and chloroquine arise from their ability to target shared cancer cell death pathways, including autophagy. The interplay between chloroquine- and ivermectin-mediated autophagy and apoptosis involves intricate molecular regulatory mechanisms.

The majority of experimental in vitro and in vivo studies have demonstrated that chloroquine, a drug with a well-established ability to suppress autophagy, can effectively sensitize cancer cells to cytotoxic agents [53], including the PI3K/Akt/mTOR inhibitor and antiparasitic drug salinomycin in breast cancer cells [195]. More recently, synergistic antitumor effects of ivermectin in combination with the antidiabetic agent metformin were reported in vitro in mouse and canine breast cancer cells, as well as in vivo in canine breast cancer xenografts in female mice. These effects were associated with the induction of autophagy, leading to apoptosis through PI3K/Akt/mTOR inhibition and increased reactive oxygen species (ROS) production [201].

Ivermectin stimulates anticancer autophagy by inhibiting the PAK1/Akt/mTOR signaling pathway, thereby promoting PAK1 degradation and enhancing autophagic flux [132]. In addition, ivermectin has been shown to induce autophagy through AMPK/mTOR inhibition [197] and via endoplasmic reticulum stress [198]. Elevated PAK1 expression has been identified in sarcomas [202], consistent with expression patterns observed in several other malignancies [202]. In sarcomas, PAK1 overexpression is associated with increased proliferative, metastatic, and angiogenic potential [203].

Chloroquine also exhibits anticancer activity through autophagy-related mechanisms, primarily by inhibiting autophagy via blockade of autophagosome–lysosome fusion, thereby preventing lysosomal degradation of cellular components and ultimately promoting cancer cell death [193]. Ivermectin induces apoptosis through endoplasmic reticulum stress and mitochondrial pathways, including Cytochrome C release, in conjunction with autophagy stimulation [132,197,198]. Autophagy acts as a double-edged sword in tumor biology; while it may support tumor growth by enabling adaptation to cellular stress and removal of damaged components, excessive or dysregulated autophagy can conversely promote tumor cell death [132,197,198]. Under physiological conditions, autophagy has been reported to suppress the induction of apoptosis [132,197,198].

Chloroquine derivatives inhibit palmitoyl-protein thioesterase 1 (PPT1) [193], and PPT1 inhibition results in direct suppression of mTORC1, a mechanism that partially overlaps with the pharmacological effects of autophagy inducers such as ivermectin. In this context, upstream autophagy signaling is activated, whereas downstream lysosomal function is effectively blocked. Consequently, PPT1 inhibitors, including chloroquine derivatives, may simultaneously harness the anticancer properties of both autophagy induction and autophagy inhibition. To some extent, the anticancer effects of chloroquine as a single agent [194] resemble those observed with the combined administration of an autophagy inducer (ivermectin) and an autophagy inhibitor (chloroquine) in the present study.

Ivermectin reduces PAK1 activity and promotes autophagy by enhancing autophagic flux, whereas chloroquine impairs lysosomal function, thereby inhibiting autophagic degradation and increasing intracellular accumulation of undegraded material. As a result, the combination of ivermectin, an autophagy inducer, and chloroquine, an autophagy inhibitor, may lead to a synergistic, non-protective, cytostatic form of autophagy. Consistently, combined treatment with chloroquine and ivermectin resulted in increased accumulation of yellow fluorescent autophagosomes, elevated endogenous LC3 puncta, and enhanced conversion of LC3-I to LC3-II, indicating that ivermectin promotes autophagic flux in breast cancer cell lines [196].

Chloroquine has previously been shown to enhance the anticancer efficacy of salinomycin by inhibiting salinomycin-induced autophagy [195]. Salinomycin, similarly to ivermectin, suppresses the PI3K/Akt/mTOR signaling pathway and activates autophagy, but over-activation of autophagy may hinder anticancer outcomes [195]. Chloroquine-mediated inhibition of salinomycin-induced autophagy potentiates its anticancer activity [195]. This mechanistic framework underlying the synergistic interaction between chloroquine and salinomycin may also be extended to explain the synergism between chloroquine and ivermectin observed in our experiments.

Ivermectin activates upstream signaling pathways that induce autophagy, whereas chloroquine functions as an autophagic phagolysosomal inhibitor. This upstream–downstream interference may create a paradoxical yet synergistic interaction, in which autophagy initiation is stimulated while its completion is blocked, ultimately promoting apoptotic cell death. Mechanistically, ivermectin enhances autophagic flux and upregulates key autophagy-related proteins, including the ATG8 family member LC3, through inhibition of the PAK1/Akt/mTOR pathway, thereby inducing autophagy-dependent apoptosis. In contrast, chloroquine suppresses autophagosome maturation. The resulting synergy arises from chloroquine’s ability to prevent cancer cells from utilizing ivermectin-induced autophagy as a survival mechanism, thereby amplifying ivermectin’s overall anticancer efficacy. Studies in breast cancer cell lines [195,196] have demonstrated that autophagy inhibition by chloroquine is critical for the synergistic anticancer effects of the ivermectin–chloroquine combination, supporting the therapeutic potential of this strategy.

Deoxycholic acid has been shown to induce autophagy in non-cancerous colonic epithelial cells by upregulating LC3 and the autophagy-related protein Beclin-1 [134]. As a cellular survival mechanism, autophagy may contribute to tumorigenesis under certain conditions [134], and increased autophagic activity has been documented in numerous cancer types. Finally, synergistic two-drug anticancer combinations involving chloroquine or ivermectin with other non-oncological agents have been evaluated in vitro and in animal xenograft models, with their mechanisms attributed to modulation of multiple anticancer signaling pathways, thereby supporting recommendations for further clinical investigation. However, to the best of our knowledge, the combined use of chloroquine and ivermectin has not yet been evaluated in animal cancer models.

It is found that the synergistic effects were recorded in combinations of chloroquine with tetrandrine on human hepatocellular carcinoma cells [204]; panobinostat in human-derived colorectal, breast, glioblastoma, head and neck cancer cells lines [141]; chemotherapeutic compounds (doxorubicin, paclitaxel, platinum-based compounds, gemcitabine, tyrosine kinases and PI3K/Akt/mTOR inhibitors), review [53]; doxorubicin in cervical cancer cells [205]; sunitinib in human breast, cervical, colorectal, hepatocellular, laryngeal and prostate cancer cells and in vivo on Erlich ascites carcinoma (mice models) [206]; salinomycin in liver cancer cells [207] and breast cancer cells [195]; epirubicin in lung cancer cells [208]; doxorubicin and paclitaxel in human breast cancer cell line [209]; obatoclax in pancreatic cancer cells [210]; sunitinib in renal cell carcinoma in vitro and in vivo [211]; temozolomide in melanoma cell lines [212]; lapatinib and gefitinib in human bladder cancer cells [213]; enzalutamide in bladder cancer cell lines [214]; anlotinib in non-small cell lung cancer cells [215]; artesunate in colorectal cancer [216]; 5-fluorouracil in colon cancer cells [47]; selumetinib in colorectal cancer cells [217]; panobinostat in ovarian cancer cells [218]; olaparib, niraparib, rucaparib and talazoparib in ovarian cancer cells; olaparib in xenografts and patient-derived xenograft models [219]; cisplatin in ovarian cancer cells [46]; nonhomologous end joining inhibitors and panobinostat in ovarian cancer cells [220]; conventional antitumor drugs in variety of cancers including glioblastoma, bladder, breast, and prostate carcinomas in clinical studies [158].

It is found that the synergistic effects were recorded in combinations of ivermectin with pitavastatin in ovarian cancer cells [113]; gemcitabine in pancreatic cancer cells and in vivo in mice [121]; nilotinib and dasatinib in myeloid leukemia cells in vitro and in vivo [94]; paclitaxel in human ovarian cancer cell lines [221]; docetaxel, cyclophosphamide and tamoxifen in breast, ovarian and prostate cancer cells and in tumor-bearing mice [105]; vemurafenib and dabrafenib in melanoma cells lines and in vivo in murine melanoma models [222]; convential chemotherapy drugs or targeted drugs on resistant digestive, urinary, reproductive, and respiratory system, breast, hematological, brain glioma and melanoma cancer cells [60]; modulated electro-hyperthermia in breast cancer in vivo mouse model [223]; bortezomib in vitro on multiple myeloma and in vivo in xenograft mouse model [224]; enzalutamide, erlotinib, cetuximab, trastuzumab, or docetaxel in lung, prostate, bladder and breast cancer cells and in vivo in nude mice [187]; chloroquine in vitro in estrogen receptor-positive and triple-negative breast cancer cell lines [196].

These investigations also confirmed that chloroquine and ivermectin possess strong synergistic anticancer potential when combined with other antitumor agents, as we found in our investigation.

The findings from the current study concur with our earlier reports demonstrating that combinations of two agents, direct or indirect inhibitors of NF-κB, exerted marked biometrical, histological and immunohistochemical anticancer effects in hamsters bearing subcutaneously inoculated fibrosarcoma, without observable toxicity [225,226]. Unlike our previous studies [225,226], in this work, we have included immunohistochemical staining for p53 and caspase 3, in addition to Ki-67, PCNA, CD34, CD31, GLUT1, iNOS, COX4, and Cytochrome C.

NF-κB and p53 exert opposing roles in cancer cells: NF-κB, as the master stimulator of cancer development and maintenance, stimulates proliferation, angiogenesis and autophagy and inhibits apoptosis. In contrast, p53, nuclear transcription factor, described as the “guardian of the genome” which prevents mutation, is associated with cell cycle arrest, angiogenesis and autophagy inhibition and apoptosis induction. The opposite functional cross-talk between NF-κB and p53 has a key role in the pathogenesis of most tumors. p21 is a major target of p53 and p53/p21 pathway is involved in cell cycle arrest. The tumor suppressor protein p53 is overexpressed and frequently mutated in many human cancers including sarcomas [227]. It has been found that p53 abnormalities may be an early event that contributes to the neoplastic transformation (Li-Fraumeni Syndrome). p53 overexpression may be related to progression toward more aggressive tumor forms. The mutant p53 protein exhibits a significantly prolonged half-life compared to its wild-type counterpart, leading to its accumulation in substantial amounts within the nuclei of transformed cells. In contrast, the wild-type p53 protein is typically present at levels too low to be detected by immunohistochemistry [227]. This accumulation of mutant p53 may impair its typical function of inhibiting cell proliferation [227].

p53 immunohistochemical staining is a marker used as a surrogate for mutational status. TP53 is a tumor suppressor gene that encodes the protein p53 which is involved in cell cycle arrest in DNA damaged cells (malignant), triggering apoptosis. A defect (mutation) of the TP53 gene was found in virtually all human (and animal) cancers [228] including soft tissue sarcoma in humans [229]. p53 immunohistochemical staining could reliably predict TP53 mutations, with an overall level of accuracy as high as 91–97% [230].

Caspase 3 antibody detects endogenous levels of cleaved caspase 3 and serves as a useful marker for detecting apoptotic cells. Caspases are a crucial mediator of apoptosis. Among them, caspase 3 is a frequently activated death protease. The modulation of apoptosis is a key characteristic of cancer, allowing tumor cells to resist cell death and foster unchecked proliferation. Elevated levels of cleaved caspase 3 have been detected in cancer cells, suggesting the presence of pro-apoptotic signaling that may not effectively induce cell death. Low caspase 3 activation indicates increased disease-free and better outcomes for cancer patients [231].

Recent results elucidate the mechanism underlying chloroquine-induced apoptosis in osteosarcoma, relevant to our sarcoma animal model. This mechanism decreased Bcl-2 expression by inhibiting its upstream regulator, STAT3, thereby relieving the suppression of Bax and caspase 3 activation. These alterations in the expression of proteins induce and promote apoptosis in osteosarcoma [12]. Ivermectin potentiates apoptosis and has a detectable effect on caspase 3/7 activation in cancer cells [113].

Apoptosis rates, as indicated by caspase-3 activation, are found to be elevated in cancerous tissues compared to non-malignant tissues [232]. This observation seems to challenge the commonly accepted notion that apoptosis is diminished in malignancies. Nevertheless, the ratio of proliferation to apoptosis may be greater in cancerous tissues than in their corresponding normal counterparts [232]. Tumor growth is the result of cell proliferation and cell loss by apoptosis. By comparison with non-neoplastic tissue, caspase 3 appeared to be upregulated in malignant tissue. Also, further analysis demonstrated a significant negative impact of caspase-3 expression on overall survival in patients [233]. Overexpression of caspase-3 seems to play a role in apoptotic pathways modulated by wild-type p53 and Bcl-2 proteins [233]. Elevated caspase 3 expression is associated with unfavorable prognostic indicators, including higher histological grade, increased cellular proliferation, and progression of malignancy [234,235].

Besides pharmacodynamic interactions, chloroquine and ivermectin can affect each other’s metabolism and clearance. Chloroquine, which is metabolized by cytochrome P450, was demonstrated as a inhibitor of P450 enzymes and drug transporters such as ABC transporters family, MDR, P-gp, MRP1, and OATP1A2 [236]. Ivermectin, which is metabolized by cytochrome P450, effectively inhibits P450, and is also an inhibitor of drug transporters such as ABC transporters family, MDR, P-gp, MRP1, and OATP1A2 [237].

In malaria treatment investigations, the combination of chloroquine and ivermectin did not show significant toxicological interactions in macaques. Consequently, this combination may be considered for further investigation in clinical trials involving human subjects [238]. The pharmacokinetic parameters of chloroquine (10 mg/kg) and ivermectin (0.3 mg/kg), when coadministered as single doses via nasogastric intubation to *Rhesus macaques*, were as follows: the maximum plasma concentration of chloroquine was 314 ± 83.5 ng/mL, while ivermectin reached 142 ± 9.9 ng/mL. The time to reach maximum concentration for both chloroquine and ivermectin was 7 ± 2 h. The clearance of chloroquine was 1.35 ± 0.4 L/h/kg, whereas ivermectin exhibited a clearance of 0.06 ± 0.01 L/h/kg. The half-life of chloroquine was 19.5 ± 0.5 h, and for ivermectin, it was 26.6 ± 6.57 h [238].

The results of safety and pharmacokinetic studies involving oral ivermectin (0.3, 0.6, and 1.2 mg/kg/day), both with and without chloroquine (10 mg/kg/day), administered over a 7-day period to *Rhesus macaques* infected with *Plasmodium cynomolgi*, demonstrated that the combination was well tolerated [239]. No adverse events were observed, and no significant pharmacokinetic drug–drug interactions were detected [239]. Plasma ivermectin concentrations, with or without coadministration of chloroquine (10 mg/kg/day), reached their maximum 2 to 4 h post-dose. The elimination half-life ranged from 11 to 28 h, and the clearance was between 0.1 and 0.04 L/h/kg [239].

The elimination half-life, clearance, and volume of distribution of chloroquine in mice, following a 50 mg/kg intraperitoneal dose administered either as a single treatment or repeatedly over 7 days, were found to be 46.6 h, 9.9 L/h/kg, and 667 L/kg, respectively [240].

The pharmacokinetic parameters of chloroquine in rats following oral administration of 5 mg/kg were as follows: the peak serum concentration ranged from 0.47 to 5.61 µg/mL, the absorption half-life was 4.05 ± 1.3 h, and the elimination half-life was 10.05 ± 3.01 h [241]. The serum concentrations measured in rats were approximately 20% of the average levels predicted in humans after a single oral dose. [241].

The chloroquine elimination half-life, clearance and volume of distribution in 19 healthy volunteers following a single 600 mg oral dose were 432 h, 0.77 L/h/kg, and 411 L/kg, respectively [242]. Despite interspecies pharmacokinetic differences, the biphasic pharmacokinetic profiles of chloroquine are consistent across mammals, and the scaling of specific parameters appears feasible for preclinical investigations [242].

The recommended oral dosing of chloroquine for the treatment of liver infections caused by protozoa in adults is 1000 mg once daily for two days, followed by 500 mg once daily for at least 2–3 weeks [243]. In middle-aged mice, equivalent dosing of 50 mg/kg administered orally over a period of 12 weeks did not result in significant pathological changes. Moreover, it prolonged the lifespan of the animals by modulating certain anticancer pathways [243].

Ivermectin was safely administered to mice via oral gavage on a multiple-dose regimen, with 10 mg/kg doses of the solution given every 12 or 24 h for the treatment of *Plasmodium* liver infection [244]. The half-life of ivermectin in rats was found to be 1–2 days following subcutaneous, oral, and intravenous administration [245].

The biological half-life of ivermectin in animals increases in the following order: swine (0.5 day), dog (1.8 day), cattle (2.8 day), sheep (2.8 day) [246].

The main pharmacokinetic parameters of ivermectin in humans, following doses of 150 μg/kg or higher, that are relevant for clinical trials include absorption half-life of 0.5–2.5 h, peak concentration (C_max_) of 40 ng/mL for a single 150 μg/kg dose, time to reach C_max_ of 4 h, protein binding (due to its high lipophilicity) greater than 90%, volume of distribution ranging from 3.1 to 3.5 L/kg, an elimination half-life of 18 h, with metabolism occurring in the liver and gut elimination through bile and feces [247].

The used doses of ivermectin in humans include 150–200 μg/kg perorally for onchocerciasis, strongyloidiasis and enterobiasis; 400 μg/kg perorally for lymphatic filariasis; 1.6 mg/kg s.c. for spinal damages and muscle spasms [248]. Importantly, in our experiments, antitumor activities of the ivermectin combination are achieved at doses equivalent to human doses that can be clinically reachable based on the usual human usage. Ivermectin is a lipophilic drug with a distribution volume of 46.9 L. The peak plasma concentration occurs 4–5 h following oral administration, with an oral clearance rate of 1.2 L/h, half-life of 19 h, plasma protein binding of 93%, and metabolism in liver with excretion by feces mainly unchanged and generating 10 metabolites (demethylated and hydroxylated) [248]. The data on ivermectin [248] may facilitate its expedited transition into clinical trials involving cancer patients.

Chloroquine is used to treat and prevent malaria, rheumatoid artritis, systemic lupus erythematosus, infections caused by different types of parasites, such as amoeba, and sometimes experimentally for COVID-19. Chloroquine has a known and good safety profile (rare retinopathy). Chloroquine phosphate tablets (US Pharmacopeia, 250 mg equivalent to 150 mg chloroquine base) were usually dosed at 500 mg (300 mg base) or 4–6 mg/kg base for adults and 10 mg/kg base for children daily [249,250]. A dose of 6 mg/kg multiplied by a conversion factor of 7.4 for hamsters equals a daily dose of 44.4 mg/kg, which is similar to the doses used in our experiments: 50 mg/kg, equivalent to 1/10 of the LD_50_ for hamsters, where LD_50_ is between 500 and 600 mg/kg. Chloroquine’s pharmacokinetic characteristics are complex due to the large volume of distribution and long half-life [249]. In cancer studies the prescribed chloroquine doses can reach up to 1200 mg daily without toxic adverse effects (including oftalmological) [250].

Ivermectin is a widely used antiparasitic endectocide with broad-spectrum activity, primarily administered orally for clinical treatment (US Pharmacopeia, 3 mg tablets) with 150–200 μg/kg of onchocerciasis, lymphatic filariasis, elephantiasis, ascariasis, cutaneous larva migrants, strongyloidiasis, and also higher doses (300–600 μg/kg) for reducing malaria transmission and for COVID-19 [251]. A dose of 0.6 mg/kg multiplied by a conversion factor of 7.4 for hamsters equals a daily dose of 4.44 mg/kg, which is similar to the doses used in our experiments: 5 mg/kg, equivalent to 1/10 of the LD_50_ for hamsters, where LD_50_ is between 25 and 50 mg/kg. Ivermectin exhibits a broad therapeutic index, with prior research [252] indicating that doses as high as 2000 μg/kg (10 times the FDA-approved dose of 200 μg/kg) are safe and generally well tolerated [252]. The maximum dose recommended for onchocerciasis treatment is 800 μg/kg [251]. Ivermectin doses for veterinary use (in cattle, sheep, pigs, and horses) are similar to those used in humans, typically ranging from 0.2 to 0.6 mg/kg. Following oral administration of ivermectin plasma concentrations are proportional to the dose. After 12 mg ivermectin doses in men (165 μg/kg), peak plasma concentrations were 46.6 ± 21.9 ng/mL, 4 h after dosing. A 3-day regimen of 600 μg/kg daily achieved a maximum ivermectin concentration identical to that achieved with a single 800 μg/kg dose in patients (above 16 ng/mL) [251].

Deoxycholic acid is rapidly absorbed and has oral LD_50_ for mice and rats above 1 g/kg and is used locally as an alternative to surgical excision in the treatment of lipomas [253]. In our experiments, deoxycholic acid is used in dose of 100 mg/kg, i.e., 1/10 LD_50_ for hamsters. In this study, deoxycholic acid was selected as a modulator of NF-κB signaling based on a comprehensive review of the literature (Table 1 and Supplemental Table S1).

A limitation of the present study is that blood and tissue concentrations of chloroquine and ivermectin were not directly measured. The statement regarding equivalence to human exposure is therefore based on body surface area dose conversion rather than experimental pharmacokinetical confirmation.

Although direct measurements were not performed, the selected doses are within ranges reported in rodents to produce plasma exposures broadly comparable to human therapeutic levels, providing a plausibility estimate of pharmacologically relevant exposure. Future studies should include quantitative pharmacokinetical analysis to validate these assumptions.

The dose-modified CI analysis was conducted according to the following formula: CI = C_A_/IC_A_ + C_B_/IC_B_ ≈ D_A_/ID_A_ + D_B_/ID_B,_ where IC represents the concentration (dose, ID) required to achieve the specified effect, and C denotes the concentration (dose, D) in combination that produces the same effect for drugs A and B. In our study, we simplified the CI analysis and applied it as follows: D denotes the dose in combination (A—chloroquine and B—ivermectin) that produces a statistically significant difference compared with the control with respect to the primary endpoints (weight, volume, and burden). The single agents did not demonstrate significant effects on the key primary endpoints, whereas in combination they produced highly statistically significant effects compared with the control at all administered doses. We have clarified that the CI for drug combination remained < 1 when comparing standard agents alone, indicating a synergistic effect. The specific calculation used to determine the CI for the chloroquine and ivermectin doses applied in the experiment is presented below (1).

(1) Combination of chloroquine 12.5 mg/kg and ivermectin 1.25 mg/kg

Chloroquine: D_A_ = 12.5 mg/kg/day, ID_A_ > 50 mg/kg/day

Ivermectin: D_B_ = 1.25 mg/kg/day, ID_B_ > 5 mg/kg/day

CI = C_A_/IC_A_ + C_B_/IC_B_ ≈ D_A_/ID_A_ + D_B_/ID_B_ < 12.5/50 + 1.25/5 = 0.5 < 1.

In contrast, in the rescue experiment, the desired effect of the combination of chloroquine and ivermectin, supplemented with deoxycholic acid, is a rescue effect (“tumor rescue”); that is, preservation of tumor mass, volume, and burden comparable to the control group, i.e., with no statistically significant difference compared to the control for all primary endpoints (weight, volume, and burden). The reported CI > 1 in the presence of deoxycholic acid reflects the analytical structure of the comparison, as the formula for calculating CI was altered by including the third term for deoxycholic acid, which itself has no single-agent anticancer efficacy in this model according to literature data (Table 1 and Supplemental Table S1). Therefore, a CI > 1 in this context should be interpreted mechanistically as antagonism. Addition of deoxycholic acid to the chloroquine and ivermectin combination did not cause statistically significant effects on the key primary endpoints (weight, volume, and burden) compared to control. We have used the Chou–Talalay formula:

CI = C_A_/IC_A_ + C_B_/IC_B_ + C_C_/IC_C_ ≈ D_A_/ID_A_ + D_B_/ID_B_ + D_C_/ID_C_,

where index A denotes chloroquine, B ivermectin, and index C denotes deoxycholic acid.

The specific calculation used to determine the CI for the combination of chloroquine, ivermectin, and deoxycholic acid at the doses applied in the experiment is presented below (2), assuming that deoxycholic acid does not produce anticancer effects.

(2) Combination of chloroquine 50 mg/kg, ivermectin 5 mg/kg, and deoxycholic acid 100 mg/kg

Chloroquine: D_A_ = 50 mg/kg/day, ID_A_ < 50 mg/kg/day

Ivermectin: D_B_ = 5 mg/kg/day, ID_B_ < 5 mg/kg/day

Deoxycholic acid: D_C_ = 100 mg/kg/day, ID_C_ ≤ 100 mg/kg/day

According to the Chou–Talalay formula for a three-drug combination:

$$CI=\frac{C_{A}}{IC_{A}}+\frac{C_{B}}{IC_{B}}+\frac{C_{C}}{IC_{C}}\approx \frac{D_{A}}{ID_{A}}+\frac{D_{B}}{ID_{B}}+\frac{D_{C}}{ID_{C}}=\frac{50}{\left(ID_{A}<50\right)}+\frac{5}{\left(ID_{B}<5\right)}+\frac{100}{\left(ID_{C}\leq 100\right)}>1$$

A limitation of the present CI analysis is that it was based on administered doses rather than measured plasma or tissue concentrations. The analysis assumes linear kinetics, which may not fully reflect in vivo pharmacokinetics. Potential sources of bias include nonlinearity, saturable absorption, or tissue-specific distribution.

While available pharmacokinetic literature [238,239,240,241,242,243,244,245,246,247,248,249,250,251,252,253] supports approximately dose-proportional exposure for chloroquine and ivermectin within the selected dosing range, CI interpretations in this study should be considered preliminary rather than definitive. Future studies with direct pharmacokinetic measurements would strengthen confidence in the calculated CI values.

The observed enhancement in antitumor efficacy following combination treatment may indicate a synergistic interaction. However, given the inherent limitations of in vivo modeling and the absence of detailed pharmacokinetic characterization, the synergy assessment should be considered preliminary. Several limitations should be acknowledged. First, the synergy assessment in vivo was based on tumor growth measurements without comprehensive pharmacokinetic profiling, which may influence the interpretation of drug–drug interactions. Second, the relatively small sample size limits statistical power and may affect the precision of the estimated combination effects. Finally, in vivo systems introduce biological variability and microenvironmental factors that are not fully accounted for in classical synergy models. Therefore, the present findings should be regarded as exploratory rather than definitive evidence of synergism.

In accordance with a new principle of adjunctive cancer treatment using combinations of multiple repurposed drugs [254], our data suggest that the chloroquine–ivermectin combination merits further investigation as part of multidrug adjuvant cancer therapy regimens employing multiple repurposed agents in treatment-resistant cancer patients [254].

Research using sarcoma models is critically important in cancer therapy development, given the highly aggressive behavior of these tumors, their resistance to existing treatments, and the high mortality associated with sarcomas [255]. Sarcomas impact an estimated 200,000 people globally annually and represent a higher proportion of cancer cases and deaths in children and adolescents compared to adults [256,257,258]. In addition, sarcomas comprise more than 20% of all pediatric solid malignancies [259].

Except that chloroquine or ivermectin exhibited antitumor activities in various dual-drug anticancer treatments, researchers also found that single chloroquine or ivermectin treatments exhibited anticancer effects in vitro or in animal models. For example, chloroquine exhibited antitumor effects in vitro or in vivo in melanoma [260], pancreatic adenocarcinoma [261], lymphoma [262], myeloid leukemia [263], lung and prostate cancer [264] and glioma [265]. Ivermectin exhibited antitumor effects in vitro or in vivo in breast cancer [169], ovarian cancer [184], colon cancer [266], leukemia [267,268] and multiple myeloma [224].

Extrapolating our observations in hamster fibrosarcoma to other cancer types or cell lines may be challenging due to the significant heterogeneity in therapeutic responses and mechanisms across various types of tumor. However, the in vitro data we obtained from different normal and malignant cell lines (normal fetal lung MRC-5 (CCL-171), human lung carcinoma A549 (CCL-185), colon carcinoma HT-29 (HTB-38), cervix carcinoma HeLa (CCL-2), and hamster fibrosarcoma BHK-21/C13) are nevertheless promising. Further preclinical evaluation of the chloroquine and ivermectin combination across additional cancer types, followed by clinical validation in sarcomas and other malignancies, is required before translation into oncology practice.

Repurposing approved non-oncologic drugs offers a promising strategy to accelerate anticancer therapies, address cancer drug resistance, and enhance patient outcomes in a cost-efficient manner [269]. For instance, over 92 clinical trials are currently investigating the anticancer potential of 14 anti-diabetic and antihypertensive agents across more than 15 different cancer types [270]. Some of these agents have advanced to Phase IV clinical trials, indicating their potential for official approval as anticancer therapies [270].

The absence of an a priori sample size calculation limits the formal statistical power of our study for the primary endpoints. Although the group size (n = 6) is consistent with prior exploratory investigations in comparable models and aligned with the 3R principle of reduction, this sample size may not be sufficient to detect smaller effect sizes with high statistical confidence. Therefore, the findings should be interpreted as exploratory and hypothesis-generating, and future studies with formal power calculations and larger cohorts are warranted.

To minimize concerns regarding multiplicity, in our study three primary endpoints were predefined in a hierarchical order: tumor weight, tumor volume, and tumor burden. Secondary endpoints were organized hierarchically into biophysical parameters (tumor diameters, tumor density, tumor surface area, tumor D_max_/density ratio, tumor area/density ratio, and tumor volume/density ratio) followed by IHC markers (p53, Ki-67, PCNA, CD34, CD31, GLUT1, iNOS, COX4, Cytochrome C, and caspase 3).

Importantly, all primary and secondary endpoints demonstrated consistent results, and no conflicting outcomes were observed. This concordance supports the robustness of the findings and suggests that multiplicity did not materially influence interpretation. Nevertheless, secondary endpoints remain exploratory, and confirmatory studies with larger cohorts and formal statistical adjustment are warranted.

A limitation of this study is that longitudinal tumor growth data were not analyzed using repeated-measures or mixed effects models. Terminal endpoints were prioritized for statistical inference due to consistent trends observed across groups and the small sample size. Future studies with larger cohorts may benefit from mixed-effects modeling to capture temporal dynamics more rigorously.

It is important to note that direct measurement of NF-κB activity was not conducted in the hamster tumor model; consequently, any inference regarding NF-κB modulation should be interpreted in the context of previously published evidence. Future studies incorporating direct assessment of NF-κB activity are warranted to validate these effects.

It is important to recognize that the MTT assay primarily reflects mitochondrial dehydrogenase activity and, therefore, may be influenced by alterations in cellular metabolism independent of direct cell death. This consideration is particularly relevant in the context of chloroquine and ivermectin, both of which are known to modulate mitochondrial function and autophagic pathways.

In the present study, the MTT assay was employed as an initial screening approach to assess changes in cell viability. While this method is widely used in anticancer drug evaluation, it does not discriminate between metabolic suppression, cytostatic effects, and irreversible cytotoxicity. Therefore, the observed reductions in MTT signal may partially reflect metabolic modulation rather than exclusively apoptotic or necrotic cell death.

Complementary orthogonal assays, such as Annexin V/PI apoptosis analysis, clonogenic survival assessment, or ATP-based luminescence measurements, would provide additional mechanistic resolution and should be incorporated in future studies to further substantiate these findings.

To the best of our understanding, the combination of chloroquine and ivermectin has not yet been investigated in clinical trials. Therefore, we recommend its consideration after additional preclinical validation, as both drugs are already approved and in clinical use.

Intended clinical applications of the combination, such as use as an adjuvant to standard chemotherapy, stand-alone repositioning, administration in the neoadjuvant setting, or implementation as maintenance therapy, should be defined following comprehensive additional preclinical and clinical evaluation. Preclinical investigations should include detailed pharmacokinetic characterization, confirmation of systemic and intratumoral exposure, validation across multiple in vitro and in vivo models, and determination of safety margins. Subsequent clinical studies are particularly warranted in sarcomas and other malignancies in which related signaling pathways (pending precise identification in future mechanistic investigations) are implicated in therapeutic resistance.

## 4. Materials and Methods

### 4.1. Animal Model

The animal experiments were conducted in accordance with our previous studies [225,226]. A total of 42 male Syrian golden hamsters were included in the experiment. Animals were randomly assigned to seven experimental groups (n = 6 per group) using a random number table generated before treatment allocation. The allocation sequence was prepared in advance to reduce potential selection bias. All animals were about 12 weeks of age and approximately 70 g in weight.

The animals were housed under standard laboratory conditions and treated in compliance with a protocol approved by the Animal Ethics Committee of the University of Novi Sad (Novi Sad, Serbia): No. 04-81/25-5 dated 22 July 2020, Doc. No. EK: Π-E-2020-07; No. 04-150/15 dated 14 March 2022, Doc. No. EK: I-2022-01; No. 04-150/15 dated 14 March 2022, Doc. No. EK: I-2022-02; and approved by the Ministry of Agriculture, Forestry and Water Management-Veterinary Directorate (Belgrade, Serbia): No. 323-07-09359/2020-05 dated 2 September 2020; No. 323-07-03995/2022-05 dated 28 March 2022; No. 323-07-03996/2022-05 dated 28 March 2022; No. 323-07-03997/2022-05 dated 28 March 2022. In the experiment, all animal groups (control and treated) consisted exclusively of an equal number of males, in order to eliminate the potential influence of sex on experimental variability. The influence of sex and the potential differential effects of sex hormones on tumor growth were not assessed in the present study.

No formal a priori sample size or power calculation was conducted for the primary endpoints (tumor volume or tumor burden). The group size (n = 6 hamsters per group) was determined based on prior experience with this model, comparable exploratory studies, and ethical considerations. In accordance with the principles of the 3Rs (Replacement, Reduction, and Refinement), the number of animals per group was reduced to the minimum considered appropriate to obtain biologically meaningful data while limiting unnecessary animal use, and to ensure statistical relevance (6 hamsters per group). Consequently, the study design should be considered exploratory.

BHK-21/C13 cells, cultured as described in our previous reports, e.g., [225,226] (Supplementary Data), were subcutaneously injected (1 mL, 2 × 10^6^ cells/mL) into the dorsal region of all hamsters by the same researcher.

The rationale for selecting Syrian hamsters as study subjects and the context for this choice are as follows: The BHK-21/C13 cell culture-induced sarcoma model in Syrian hamsters is highly reproducible. Tumors are consistently induced at the inoculation site of the BHK-21/C13 cell suspension; they are solitary, massive, and do not metastasize. The tumor cells closely resemble the BHK-21/C13 cells in vitro and grow without significant influence from the host’s immune mechanisms. Immunologically, Syrian hamsters do not recognize BHK-21/C13 cells as tumorigenic, which allows the tumor to grow into large masses. This tumor is locally infiltrative. BHK-21/C13 cells are tumorigenic only in hamsters (with the exception of nude mice). Only intact live cells are tumorigenic, not DNA or cell extracts; thus, the BHK tumor is essentially an in vivo culture of BHK-21/C13 cells. We consider this model ideal for pharmacological testing of antitumor agents, as it is not influenced by immune rejection, unlike other animal tumor models.

Although the specific BHK-21/C13 fibrosarcoma model is not a spontaneous human cancer counterpart, its reproducible induction, measurable growth kinetics, host–tumor interactions, influence of vasculature and transplantability in vivo dovetail with the broader rationale for using this model in translational oncology: to simulate tumor establishment and growth under controlled conditions that facilitate testing of experimental therapeutics and mechanistic hypotheses before clinical application.

Therefore, the use of the BHK-21/C13 hamster sarcoma model is justified within the context of comparative oncology and preclinical pharmacological testing, serving as one of several complementary in vivo platforms that inform mechanistic understanding and therapeutic evaluation relevant for human cancers.

The primary cultures that gave rise to the Syrian hamster fibroblast cell line BHK-21 were established in March 1961 from the kidneys of five one-day-old hamsters originating from litter No. 21. Nineteen days after an alteration in growth kinetics was initially detected, eight clonal populations were derived from single cells cultured in microdrops of medium under mineral oil. Among these, the clone designated BHK-21/13 (C13) has been extensively characterized and represents the progenitor of the majority of BHK cell stocks currently distributed to laboratories worldwide.

The rapid proliferation rate and high cloning efficiency of BHK-21/C13 cells, together with their relatively low chromosome number, render this line particularly suitable for a variety of preclinical experimental applications [271]. The BHK-21 fibroblast line derived from Syrian hamster tissue is widely utilized in studies of viral replication and neoplastic transformation in vitro [272,273,274,275]. Certain BHK-21 clones, including C13, have been shown to induce tumors in hamsters [275].

As reported previously [275,276], BHK cells retain the capacity to proliferate within hamster tissues and to initiate tumor formation in vivo. Subcutaneous inoculation of 10^7^ BHK-21 clone 13 cells resulted in the development of fibrosarcoma, leading to animal mortality within approximately 40 days [275]. These findings confirm the tumorigenic potential of the C13 clone and support its use as a reproducible experimental model for investigating mechanisms of tumor growth and therapeutic intervention.

The cytogenetic and tumorigenic characteristics of a spontaneously transformed baby hamster kidney cell line (BHK-21) have been characterized [277]. The cultured monolayers consisted of fibroblast-like, epithelial-like, and multinucleated syncytial cells, indicating morphological heterogeneity within the population. Cytogenetic analysis demonstrated a modal chromosome number of 43, accompanied by aneuploidy and structural chromosomal alterations. In vivo tumorigenicity was assessed by intradermal implantation of BHK-21 cells into hamsters, which resulted in the development of polymorphous sarcomas in 100% of animals. The resulting tumors exhibited a transplantability rate of approximately 90% upon serial passage and resumed measurable growth within 4–5 days following grafting [277].

The cytogenetic analyses described in [278] establish that BHK-21/C13 cells possess a reproducible, although aneuploid and structurally altered, karyotype. These findings support their utility as a defined and monitorable cellular system, since chromosomal composition and marker stability can be tracked across passages. In this sense, the studies [278,279] indirectly support their application in tumorigenicity assays by demonstrating that the genetic background of the line is sufficiently characterized for experimental use. Malignant transformation in BHK-21 clone 13 cells can be induced under controlled conditions and the transformed phenotype may be conditionally expressed. This indicates that the line is responsive to carcinogenic stimuli and capable of forming tumor-associated traits, a property that underlies its historical use in in vivo tumor induction models [279].

In the experiment, we applied human equivalent daily doses for hamsters (i.e., 7.4 × human dose, ref. [280,281,282]), which was ~10 times smaller than LD_50_ for all 3 examined drugs and even smaller doses were used for the dose–response experiment.

Hamster doses were calculated to be equivalent to standard human doses on a mg/kg/day basis using body surface area normalization. Specifically, the hamster equivalent dose was determined using the following equation:

Hamster equivalent dose = Standard human dose × 7.4

where the conversion factor of 7.4 reflects the interspecies body surface area scaling for hamsters relative to humans [280,281,282].

Since blood and tissue concentrations were not directly measured in this study, human exposure equivalents were estimated based on previously published animal-to-human dose conversion data [280,281,282] and supported by available pharmacokinetic literature [238,239,240,241,242,243,244,245,246,247,248,249,250,251,252,253].

Chloroquine phosphate tablets (USP; 250 mg, equivalent to 150 mg chloroquine base) are typically administered at a dose of 500 mg (300 mg base) or 4–6 mg/kg of chloroquine base per day in adults, and 10 mg/kg of chloroquine base per day in children [249,250].

Using the upper adult dose of 6 mg/kg and applying the interspecies body surface area conversion factor of 7.4 for hamsters, the calculated equivalent dose is 44.4 mg/kg/day. This value is comparable to the dose used in our experiments (50 mg/kg/day), which corresponds to approximately 1/10 of the LD_50_ in hamsters. The reported LD_50_ for hamsters ranges between 500 and 600 mg/kg.

Ivermectin is a broad-spectrum antiparasitic endectocide that is clinically administered orally (USP; 3 mg tablets) at doses of 150–200 μg/kg for the treatment of onchocerciasis, lymphatic filariasis, elephantiasis, ascariasis, cutaneous larva migrans, and strongyloidiasis. Higher doses (300–600 μg/kg) have also been used to reduce malaria transmission and have been investigated in the context of COVID-19 [251].

Applying the upper dose of 0.6 mg/kg and multiplying by the interspecies body surface area conversion factor of 7.4 for hamsters yields a calculated equivalent daily dose of 4.44 mg/kg. This value is comparable to the dose used in our experiments (5 mg/kg/day), which corresponds to approximately 1/10 of the LD_50_ in hamsters. The reported LD_50_ in hamsters ranges between 25 and 50 mg/kg.

Deoxycholic acid has been reported to lower serum cholesterol following oral administration of 750 mg/day for 3–4 weeks. The effects of this dose on bile acid kinetics were investigated in healthy volunteers [283]. The equivalent dose for hamsters, calculated based on body surface area, is approximately 100 mg/kg/day (750 mg/day = 12 mg/kg/day, 12 mg/kg/day × 7.4 ≈ 100 mg/kg/day), where 7.4 represents the interspecies body surface area conversion factor for hamsters [280,281,282]. This hamster dose (100 mg/kg/day), corresponding to the oral human dose, is well below 25% of the estimated hamster LD_50_ (oral LD_50_: mouse 1000 mg/kg, rat 1370 mg/kg). This rationale underlies the selection of 100 mg/kg/day deoxycholic acid for hamsters in our study.

The selected daily dose of deoxycholic acid (100 mg/kg body weight) in the present study is consistent with previously published rodent models in which deoxycholic acid administration elicited IL-1β induction and subsequent NF-κB activation in the absence of overt toxicity [284,285]. Animals received deoxycholic acid daily for the duration of the treatment protocol [284,285].

The treatment protocols were as follows: (1) untreated control; treated daily with (2) chloroquine 50 mg/kg (LD_50_ = 500–600 mg/kg); (3) ivermectin 5 mg/kg (LD_50_ = 25–50 mg/kg); (4) combination of chloroquine 50 mg/kg and ivermectin 5 mg/kg; (5) combination of chloroquine 50 mg/kg, ivermectin 5 mg/kg and deoxycholic acid 100 mg/kg (LD_50_ = 1000–1370 mg/kg); (6) combination of chloroquine 25 mg/kg and ivermectin 2.5 mg/kg; (7) combination of chloroquine 12.5 mg/kg and ivermectin 1.25 mg/kg.

The drugs were delivered via a gastric probe on a daily basis, following cancer cell inoculation. The drugs (chloroquine from Remedica Ltd., Limassol, Cyprus; ivermectin from Veterinary Institute Subotica Ltd., Subotica, Serbia, and deoxycholic acid from Merck KGaA, Darmstadt, Hesse, Germany) were administered orally at a volume of 0.5 mL of water per 100 g of hamster body mass. Sterile distilled water was used as the vehicle. Dosing was performed consistently at the same time each day (between 09:00 and 10:00 a.m.) to minimize potential circadian variation in drug absorption and metabolism. Animals were not fasted prior to drug administration and had ad libitum access to food and water throughout the study.

The absolute single doses were individually calculated based on body mass, maintaining a consistent relative dose (mg/kg). However, the gavage volume (~0.5 mL) was determined according to the standard daily drinking volume (10 mL per 100 g of hamster body weight), and was approximately 20 times lower than the volume the hamsters would typically consume daily.

Humane endpoints were established as previously reported [225,226] (Supplementary Data).

All hamsters were evaluated for loss of consciousness 5 min after an intraperitoneal administration of 90 mg/kg pentobarbital, based on the absence of visible respiration, lack of response to digital palpation, and absence of reaction to a toe pinch. This assessment was conducted to euthanize the hamsters 17 days post-tumor cell inoculation.

Upon confirmation of loss of consciousness, total cardiac exsanguination (3–5.5 mL) was performed for biochemical and hematological blood analysis. Following the animal’s death, vital organs (heart, lungs, stomach, intestine, liver, kidneys, and brain) were harvested for pathological, histological, and toxicological examination.

During the experiment, the body weights of the hamsters were assessed daily. Treatment was initiated immediately following cancer cell inoculation, on the same day. Tumor measurements were performed in a standardized manner starting on day 7 post-inoculation, when tumors became palpable. Tumor diameters were measured using calipers, and tumor volume was calculated at predefined time intervals using the ellipsoid volume formula.

All hamsters remained in good condition throughout the study, with no euthanasia or mortality occurring before the completion of the experiment. In the experiment, the maximum tumor diameters did not exceed 3.5 cm, and the maximum tumor burden remained below 6%, in accordance with internationally recognized standards (Supplementary Data). After tumor excision, tumors were weighed, their three diameters precisely measured, the surface area calculated using the ellipsoid formula, and tumor volumes determined by the standard water displacement method [225,226]. Tumor burden (tumor weight relative to hamster body weight), tumor density, tumor D_max_/density ratio, tumor area/density ratio, and tumor volume/density ratio were determined as previously described [225,226]. A tumor section up to 5 mm thick was taken from the region representing the widest circumference of the tumor nodule for histological analysis. Tumor slices (4 µm) were then prepared for subsequent pathohistological and immunohistochemical evaluation.

The combination effect was assessed using Combination Index (CI) analysis to determine synergism (CI < 1), an additive effect (CI = 1), or antagonism (CI > 1). The CI was calculated using the following formula: CI = C_A_/IC_A_ + C_B_/IC_B_, where IC represents the concentration (dose) required to achieve the specified effect, and C denotes the concentration (dose) in combination that produces the same effect for drugs A and B [200].

Three hierarchically organized primary endpoints were defined for this study: tumor weight (most direct measure of tumor mass), followed by tumor volume, and then tumor burden.

All other outcome measures were classified as secondary endpoints and evaluated in a hierarchical manner:

(1) Biophysical tumor parameters, including tumor diameters, tumor density, tumor surface area, tumor D_max_/density ratio, tumor area/density ratio, and tumor volume/density ratio;
(2) Immunohistochemical (IHC) parameters p53, Ki-67, PCNA, CD34, CD31, GLUT1, iNOS, COX4, Cytochrome C, and caspase 3.

Analyses were exploratory in nature; however, concordant trends were observed across all primary and secondary endpoints, indicating that multiplicity did not introduce conflicting interpretations.

Tumor volumes were measured longitudinally throughout the study. Statistical analysis focused on terminal endpoints, including tumor weight, tumor volume, and tumor burden at the end of the experiment. While repeated-measures or mixed effects modeling can be applied to longitudinal data, the present study analyzed terminal endpoints due to the consistent trends observed across all groups, the primary purpose of longitudinal measurements being monitoring of growth progression, and the limited sample size (n = 6 per group).

### 4.2. Histological Staining Procedures

Standard hematoxylin–eosin (HE) staining was performed to evaluate tumor growth, tissue infiltration, and the extent of necrosis and hemorrhagic areas. In addition to the principal HE staining, immunohistochemical p53, Ki-67, PCNA, CD34, CD31, GLUT1, iNOS, COX4, Cytochrome C, and caspase 3 staining was carried out as previously described [225,226] (Supplementary Data) to assess mutational status (p53); tumor proliferation (Ki-67 and PCNA); neoangiogenesis (CD34 and CD31); glucose metabolism (GLUT1); NO metabolism (iNOS) and apoptosis (COX4 and Cytochrome C combined with caspase 3) in animal experiments. To validate and confirm results for the key tumor growth processes, two markers were employed for proliferation and angiogenesis, and 3 markers were used for apoptosis. Altered COX4 expression suggests mitochondrial involvement and, together with cytochrome C release and caspase-3 cleavage, supports activation of the intrinsic apoptotic pathway.

Primary antibodies (Thermo Fisher Scientific, Waltham, MA, USA; Abcam, Cambridge, UK) were used, with antigen retrieval performed on tissue sections in goat serum (Sigma-Aldrich, St. Louis, MO, USA). A horseradish peroxidase-conjugated goat polyclonal rabbit immunoglobulin G secondary antibody (Abcam, Cambridge, UK) was applied, followed by visualization with chromogen (Dako; Agilent Technologies, Inc., Santa Clara, CA, USA). Staining was carried out using Mayer’s hematoxylin (cat. no. MHS16, Sigma-Aldrich Inc., St. Louis, MO, USA), and immunoexpression was evaluated using a Leica microscope with a Leica camera (Leica Microsystems GmbH, Wetzlar, Germany) and UTHSCSA Image Tool for Windows 3.0 [286], as previously described in our reports [225,226].

### 4.3. Blood Hematological Analyses and Biochemical Tests

Erythrocytes, leukocytes, lymphocytes, monocytes, granulocytes, platelets, hemoglobin, hematocrit, mean corpuscular volume, mean corpuscular hemoglobin, mean corpuscular hemoglobin concentration, glucose, serum proteins, albumins, and sedimentation rates were analyzed using the same methodology as previously described [225,226] (Supplementary Data).

### 4.4. In Vitro Antiproliferative Assay

The tested treatments (chloroquine, ivermectin, and their combination) were evaluated for their in vitro antiproliferative effects in hamster fibrosarcoma BHK-21/C13 cells (CCL-10, ATCC, Manassas, VA, USA) and in human cancer cell lines: lung carcinoma A549 (CCL-185, ATCC, Manassas, VA, USA), colon carcinoma HT-29 (HTB-38, ATCC, Manassas, VA, USA), cervical carcinoma HeLa (CCL-2, ATCC, Manassas, VA, USA), and normal human fetal lung MRC-5 (CCL-171, ATCC, Manassas, VA, USA) cells. All cell lines were obtained from the American Type Culture Collection, authenticated, and subjected to mycoplasma testing. The cell lines were cultured in DMEM with 4.5 g/L glucose, supplemented with 10% FBS and 1% penicillin-streptomycin, in an incubator at 37 °C with 5% CO_2_. Cytotoxicity was assessed using the standard MTT assay [287], after exposure to chloroquine concentrations ranging from 10 to 250 µM, ivermectin concentrations ranging from 1 to 25 µM, and their combinations for 48 h at 37 °C.

Ivermectin and chloroquine were initially dissolved in dimethyl sulfoxide (DMSO) to prepare concentrated stock solutions and subsequently diluted in complete culture medium immediately prior to treatment. The final DMSO concentration in all experimental and vehicle control wells did not exceed 0.1% (*v*/*v*), a level generally considered non-cytotoxic in tetrazolium-based viability assays. Vehicle controls contained the same DMSO concentration as treated samples.

To ensure compound stability and homogeneous exposure, each working dilution was prepared in pre-warmed medium and examined for visible turbidity or precipitation before addition to cells. Concentrations showing any evidence of insolubility were excluded and freshly prepared to maintain full solubility throughout the exposure period.

To avoid confounding effects related to osmotic imbalance, equal volumes of solvent were used across all treatment groups. Culture media were maintained under standard physiological osmolarity conditions (~300 mOsm/kg), and the addition of drug solutions at the applied concentrations did not measurably alter osmotic balance.

In summary, solvent effects were controlled by maintaining DMSO at ≤0.1% in all wells (including controls), compound dilutions were visually inspected to avoid precipitation, and osmolarity differences were mitigated by consistent medium composition and vehicle controls.

The antiproliferative effect was quantified as the half maximal inhibitory concentration (IC_50_) with confidence intervals (95%). Selectivity index (SI) was calculated as (MRC-5 IC_50_)/(cancer IC_50_).

### 4.5. Statistical Analysis

Mean ± SD or ±SEM values and correlation analysis were calculated, and one-way ANOVA followed by a Student–Newman–Keuls post hoc test was conducted using TIBCO Statistica 13.3.1 software (TIBCO Software, Inc., San Ramon, CA, USA), as described in our previous studies [225,226]. *p*-values less than 0.05 were considered statistically significant. To further assess significance, the two-sided Mann–Whitney U test (for comparing medians) was also performed in addition to the parametric tests.

## 5. Conclusions

Chloroquine and ivermectin, synergistically, significantly inhibited fibrosarcoma in hamsters, checked, confirmed and verified by rescue experiment with NF-κB stimulator deoxycholic acid, dose–response experiment in human equivalent dose range, and in vitro experiments on various malignant cell lines.

Captivating anticancer effects of chloroquine and ivermectin combination are likely due to synergistic inhibition upstream, downstream or directly of NF-κB.

The combination therapy of chloroquine and ivermectin may synergize their anticancer effects, offering potential advantages for fibrosarcoma and possibly other cancers in chemoprevention or adjuvant settings. These advantages include enhanced efficacy, reduced dosages of the individual agents (thereby minimizing adverse reactions), and possible circumvention of drug resistance. However, these results should be interpreted as preliminary preclinical evidence. Prior to clinical consideration in oncology, further preclinical studies are required, including detailed mechanistic confirmation, pharmacokinetic characterization, confirmation of systemic and intratumoral drug exposure, validation across multiple tumor models, and assessment of safety margins. Collectively, our data provide a rationale for continued preclinical investigation of this drug combination and, after that, possible clinical consideration. Prior to defining a specific clinical scenario (adjuvant, neoadjuvant, stand-alone repositioning, or maintenance setting), additional studies are required. Additional clinical studies will be crucial to definitively determine the role of chloroquine and ivermectin combination therapy in the treatment of fibrosarcoma and other cancer types.

## Figures and Tables

**Table 1:** Separate anticancer effects of chloroquine and ivermectin vs. reciprocal deoxycholic acid modulation of the same established oncological targets/markers in different studies (detailed review in Supplemental Table S1).

Anticancer Therapy Targets Tumor Promoters (P) Tumor Suppressors (S)	Chloroquine Effect: Ref. No.	Ivermectin Effect: Ref. No.	Deoxycholic Acid Effect: Ref. No.
**(1) Transcription factors**			
**NF-κB (P)**	Inhibition: [2]	Inhibition: [3,4]	Stimulation: [5,6]
**p65 (Rel A) (P)**	Inhibition: [7]	Inhibition: [8]	Stimulation: [6]
**Integrins (P)**	Inhibition: [9]	Inhibition: [10]	Stimulation: [11]
**STATs (P)**	Inhibition: [12]	Inhibition: [13,14,15]	Stimulation: [16]
**Nrf2 (S)**	Stimulation: [17]	Modulation: [18]	Modulation: [19]
**Nanog, KFL4, Sox2, Oct4 (P)**	Inhibition: [20]	Inhibition [21]	Stimulation: [22,23]
**JNKs (P)**	Inhibition: [24]	Inhibition: [25]	Stimulation: [26]
**HES1, K13, MUC2 (P)**	Modulation: [27]	Modulation: [28,29]	Modulation: [30]
**VDR (S)**	Inhibition: [31]	Inhibition: [32]	Stimulation: [33]
**Hippo\YAP (P)**	Inhibition: [34,35]	Inhibition: [36]	Stimulation: [37]
**TCFs, LEFs (P)**	Modulation: [38,39]	Inhibition: [40]	Stimulation: [41,42]
**(2) Cell cycle proteins**			
**Cyclin D_1_, CDKs (P)**	Inhibition: [43,44]	Inhibition: [40]	Modulation: [45]
**p21, p27 (S)**	Stimulation: [46,47]	Stimulation: [48,49]	Inhibition: [50] Modulation: [51]
**Ki-67 (P)**	Inhibition: [52,53]	Inhibition: [54]	Stimulation: [55]
**PCNA (P)**	Inhibition: [43]	Inhibition: [3]	Stimulation: [56]
**Akt (P)**	Inhibition: [57]	Inhibition: [18]	Stimulation: [58]
**mTOR (P)**	Inhibition: [59]	Inhibition: [60]	Stimulation: [61]
**EGFR (P)**	Inhibition: [62]	Inhibition: [18]	Stimulation: [63]
**Raf, RAS, MAPK (P)**	Inhibition: [64,65]	Inhibition: [60]	Stimulation: [26]
**PI3K (P)**	Inhibition: [57]	Inhibition: [66]	Stimulation: [67,68]
**(3) Cell signaling cascades proteins**			
**MDR (P)**	Inhibition: [69]	Inhibition: [60]	Stimulation: [70,71]
**p65: see (1)**			
**IκB (P)**	Inhibition: [2]	Inhibition: [54]	Stimulation: [6]
**Hedgehog (P)**	Inhibition: [72]	Inhibition: [15]	Stimulation: [22]
**ABC (P)**	Inhibition: [69]	Inhibition: [73,74]	Modulation: [75]
**PKC (P, S)**	Inhibition: [76]	Inhibition: [77]	Stimulation: [78]
**JAKs (P)**	Inhibition: [79,80]	Inhibition: [81]	Stimulation: [22]
**Wnt/β-catenin (P)**	Inhibition: [35,82]	Inhibition: [82]	Stimulation: [41]
**ILs (P)**	Inhibition: [83]	Inhibition: [84,85]	Stimulation: [78,86]
**IGF-1, IGF-2 (P)**	Inhibition: [87]	Inhibition: [88]	Stimulation: [89]
**PTEN (S)**	Stimulation: [80]	Stimulation: [90]	Modulation: [91] Inhibition: [92]
**Eps8 (P)**	Inhibition: [93]	Inhibition: [10,94]	Stimulation: [78] Modulation: [95]
**(4) Apoptosis and/or necroptosis associated proteins:**			
**Bcl-2 (P)**	Inhibition: [57,96]	Inhibition: [15]	Stimulation: [97]
**Bax (S)**	Stimulation: [57]	Stimulation: [15]	Inhibition: [98]
**Cytochrome C (S, P)**	Modulation: [99]	Modulation: [100]	Modulation: [45]
**PARPs (P)**	Inhibition: [101]	Inhibition: [102]	Modulation: [103]
**p53 (S)**	Modulation: [104]	Inhibition: [105]	Inhibition: [106]
**p63 (S)**	Modulation: [107]	Stimulation: [108]	Inhibition: [109]
**p73 (P, S)**	Inhibition: [83]	Inhibition: [85]	Stimulation: [110]
**caspase 3, 7, 8, 9, 10, 12 (S)**	Stimulation: [12,111]	Stimulation: [48,108,112,113,114]	Inhibition: [115]
**p38MAPK (P, S)**	Inhibition: [116,117]	Inhibition: [25]	Stimulation: [118,119]
**Mcl-1 (P)**	Inhibition: [120]	Inhibition: [121]	Stimulation: [122]
**p65: see (1)**			
**Nrf2: see (1)**			
**(5) Death receptors on the cell surface**			
**FAS (S)**	Stimulation: [123]	Stimulation: [15]	Stimulation: [26] Modulation: [115]
**TRAIL (S)**	Stimulation: [124,125,126,127]	Stimulation: [15]	Modulation: [128]
**(6) Autophagy (ATG) related proteins**			
**PI3K: see (2)**			
**ATG (S)**	Inhibition: [46,129,130,131]	Stimulation: [132] Modulation: [133]	Stimulation: [51,134,135]
**LC3 (S)**	Inhibition: [136]	Modulation: [137]	Stimulation (acute exposure) or Inhibition (chronic exposure): [138]
**Beclin1 (P, S)**	Inhibition: [139]	Modulation: [137]	Stimulation (acute exposure) or Inhibition (chronic exposure): [138]
**p62 (P)**	Inhibition: [136]	Modulation: [137]	Stimulation (acute exposure) or Inhibition (chronic exposure): [138]
**(7) Antioxidant defense proteins and other targets/markers**			
**ROS (P, S)**	Inhibition: [140] Stimulation: [141]	Stimulation: [114,142]	Stimulation: [143,144,145]
**COX-2 (P)**	Inhibition: [146,147]	Inhibition: [148,149]	Stimulation: [119,150,151,152]
**iNOS/NO (P)**	Inhibition: [153]	Inhibition: [148]	Stimulation: [154]
**TNF-α (P)**	Inhibition: [83]	Inhibition: [85]	Stimulation: [110]
**PGE2 (P)**	Inhibition: [155,156]	Inhibition: [148]	Stimulation: [157]
**(8) Angiogenesis and tumor microenvironment associated proteins**			
**CD31 (=PECAM1), CD34 (P)**	Inhibition: [158]	Inhibition: [159]	Stimulation: [160]
**HIFs (1α, 1β, 2α, 2β) (P)**	Inhibition: [161]	Inhibition: [162]	Stimulation: [163,164]
**VEGF (P)**	Inhibition: [165]	Inhibition: [166,167]	Stimulation: [160]
**MEK/ERK (P)**	Inhibition: [123,168]	Inhibition: [18]	Stimulation: [58]
**p38MAPK see: (4)**			
**CXC, CXCL12 (=SDF1), CXCR4 (P)**	Inhibition: [20]	Modulation: [169]	Inhibition: [170]
**FAK (P)**	Inhibition: [93]	Inhibition: [10]	Modulation: [95]
**(9) Cellular metabolism and metabolic reprogramming associated proteins**			
**GLUTs (P)**	Stimulation: [171]	Inhibition: [81]	Modulation: [172,173]
**TLRs (TLR1-24) (P)**	Inhibition: [174,175]	Inhibition: [3]	Stimulation: [176]
**(10) Embryonic development associated proteins**			
**TGF-β/SMAD(P)**	Inhibition: [177]	Inhibition: [178]	Modulation: [179]
**FAK: see (8)**			
**(11) Cancer stem cells (CSC) associated proteins**			
**STATs: see (1)**			
**Nanog/KFL4/Sox2/Oct4: see (1)**			
**JAK: see (3)**			
**FAK: see (8)**			
**(12) Membrane receptors**			
**PANX1 (P)**	Modulation: [180]	Modulation: [169]	Stimulation: [78] Modulation: [181]
**P2X4, P2X7 (P)**	Stimulation: [182]	Stimulation: [182] Modulation: [169]	Stimulation: [78] Modulation: [181]
**CXCR4 (P)**	Inhibition: [20]	Modulation: [169]	Inhibition: [170]
**(13) Membrane bound proteins (transcription factors) shuttling between the cytoplasm and nucleus:**			
**Notch (P)**	Inhibition: [183]	Inhibition: [184]	Inhibition: [30,185]
**EGFR: see (2)**			
**Wnt/β-catenin: see (3)**			
**IGF-1/IGF-1R: see (3)**			
**(14) Oncogen addiction targets**			
**PTKs (P)**	Inhibition: [93]	Inhibition: [10]	Modulation: [95]
**MYC (C-, N-, L-MYC) (P)**	Inhibition: [186]	Inhibition: [187]	Stimulation: [188]
**cABL (P)**	Inhibition: [93]	Inhibition: [10,94]	Modulation: [78,95]
**Raf-1/RAS: see (2)**			
**MEK/ERK: see (8)**			
**PI3K: see (2)**			
**Akt: see (2)**			
**mTOR: see (2)**			
**(15) Tumor suppressor rescue targets/markers**			
**cABL: see (14)**			
**PTEN: see: (3)**			
**(16) Some special cancer related processes (integrally evaluated)**			
**Apoptosis (S)**	Stimulation: [111,127,189]	Stimulation: [48,66]	Inhibition: [6,58]
**Necroptosis/pyroptosis/ferroptosis (S)**	Stimulation: [158]	Stimulation: [190]	Inhibition: [58]
**Neoangiogenesis (P)**	Inhibition: [191,192]	Inhibition: [159]	Stimulation: [160]
**Autophagy (S): see also (6)**	Inhibition: [193,194,195]	Stimulation: [132,196,197,198]	Stimulation: [51,134,135] Stimulation (acute exposure) or Inhibition (chronic exposure): [138]
**PAK1 (P)**	Inhibition: [199]	Inhibition: [199]	

**Table 2:** Characteristics of hamsters and biophysical characteristics of extirpated tumors.

	Hamster		Tumor								
No	Weight at Start (g)	Weight at End (g)	Tumor Weight (g)	D_max_ (cm) ^a^	Volume (cm^3^)	Tumor Burden (%)	Density (g/cm^3^)	Area (cm^2^)	D_max_/Density (cm^4^/g)	Area/Density (cm^5^/g)	Volume/Density (cm^6^/g)
Control group											
1	67	95	4.14	2.7	3.6	4.4	1.150	11.91	2.35	10.36	3.13
2	69	97	5.57	2.9	4.8	5.7	1.161	14.54	2.50	12.52	4.13
3	70	105	7.51	3.4	6.3	7.1	1.192	17.67	2.85	14.80	5.29
4	68	94	6.65	3.1	5.7	7.1	1.166	16.36	2.66	14.03	4.89
5	70	108	7.77	3.3	6.5	7.2	1.195	17.75	2.76	14.85	5.44
6	61	85	4.61	2.4	4.0	5.4	1.152	12.50	2.08	10.85	3.47
Mean			6.04	2.97	5.15	6.15	1.169	15.12	2.53	12.90	4.39
±SD			1.38	0.38	1.21	1.16	0.020	2.55	0.28	1.97	0.97
Group treated with chloroquine											
1	60	78	3.95	2.1	3.4	5.1	1.132	12.14	1.81	10.45	2.93
2	62	74	5.30	2.5	4.5	7.2	1.178	15.64	2.12	13.28	3.82
3	66	91	5.55	2.5	4.7	6.1	1.180	15.64	2.12	1325	3.98
4	71	87	4.67	2.2	4.0	5.4	1.168	13.41	1.88	11.48	3.42
5	70	90	4.42	2.1	3.8	4.9	1.164	12.99	1.80	11.16	3.26
6	64	80	1.62	1.7	1.4	2.0	1.155	7.70	1.47	6.67	1.21
Mean			4.25	2.18	3.63	5.12	1.167	12.92	1.87	11.05	3.10
±SD			1.41	0.30	1.19	1.74	0.010	2.93	0.24	2.43	1.00
Group treated with ivermectin											
1	61	82	2.83	1.9	2.5	3.4	1.130	9.06	1.68	8.02	2.21
2	66	87	6.32	2.6	5.4	7.3	1.170	15.14	2.22	12.94	4.62
3	69	91	6.80	2.5	5.8	7.5	1.173	15.64	2.13	13.33	4.94
4	71	100	5.37	2.2	4.6	5.4	1.168	13.41	1.88	11.48	3.94
5	66	92	5.62	2.3	4.8	6.1	1.170	13.84	1.97	11.83	4.10
6	68	81	1.24	1.5	1.1	1.5	1.125	5.29	1.33	4.70	0.98
Mean			4.70	2.17	4.03	5.20	1.156	12.05	1.87	10.38	3.47
±SD			1.99	0.41	1.84	2.34	0.022	4.07	0.32	3.36	1.54
Group treated with chloroquine and ivermectin											
1	70	96	0.134	1.0	0.13	0.14	1.031	1.35	0.97	1.31	0.126
2	63	85	0.051	0.8	0.05	0.06	1.022	0.89	0.78	0.87	0.049
3	65	93	0.092	1.0	0.09	0.10	1.027	1.21	0.97	1.18	0.088
4	60	87	0.031	0.8	0.03	0.04	1.020	0.80	0.78	0.78	0.029
5	74	101	0.269	1.1	0.26	0.27	1.035	2.16	1.06	2.09	0.251
6	65	90	0.031	0.6	0.03	0.03	1.021	0.59	0.59	0.58	0.029
Mean			0.101	0.883	0.098	0.107	1.026	1.193	0.86	1.135	0.0953
±SD			0.083	0.183	0.088	0.090	0.006	0.515	0.17	0.538	0.0685
Group treated with chloroquine, ivermectin and deoxycholic acid											
1	61	79	2.24	2.3	1.9	2.83	1.182	7.81	1.95	6.61	1.61
2	63	84	4.54	2.0	4.0	5.40	1.135	10.17	1.76	8.96	3.52
3	70	80	3.06	1.8	2.7	3.82	1.133	9.44	1.59	8.33	2.38
4	73	109	9.67	3.5	8.6	8.87	1.125	22.64	3.11	20.12	7.64
5	67	94	6.46	2.5	5.5	6.87	1.175	15.17	2.13	12.91	4.68
6	63	88	4.68	2.2	4.0	5.32	1.170	12.91	1.88	11.03	3.42
Mean			5.11	2.38	4.45	5.52	1.153	13.02	2.07	11.33	3.88
±SD			2.43	0.60	2.37	2.16	0.025	5.39	0.54	4.83	2.12
Group treated with 1/2 doses of chloroquine and ivermectin											
1	75	100	0.95	1.9	0.88	0.95	1.080	4.86	1.76	4.50	0.815
2	67	96	0.72	1.6	0.67	0.75	1.078	3.94	1.48	3.65	0.622
3	64	92	0.52	1.5	0.49	0.56	1.063	3.26	1.41	3.07	0.461
4	72	102	0.84	1.7	0.78	0.82	1.071	4.42	1.59	4.13	0.728
5	62	86	0.23	1.2	0.22	0.27	1.050	1.94	1.14	1.85	0.210
6	64	80	0.26	1.0	0.25	0.32	1.054	2.00	0.95	1.90	0.237
Mean			0.59	1.48	0.55	0.612	1.066	3.40	1.39	3.18	0.512
±SD			0.27	0.33	0.28	0.276	0.012	1.23	0.30	1.12	0.253
Group treated with 1/4 doses of chloroquine and ivermectin											
1	74	99	3.20	2.0	2.85	3.23	1.124	9.79	1.78	8.71	2.54
2	65	94	1.93	1.8	1.72	2.05	1.120	7.03	1.61	6.28	1.54
3	62	87	1.48	1.5	1.33	1.70	1.115	5.86	1.34	5.26	1.19
4	73	105	4.12	2.4	3.63	3.92	1.135	11.65	2.11	10.26	3.20
5	77	102	5.35	2.8	4.69	5.24	1.140	14.11	2.46	12.38	4.11
6	65	90	1.26	1.4	1.13	1.40	1.119	5.30	1.25	4.74	1.01
Mean			2.89	1.98	2.56	2.92	1.126	8.96	1.76	7.94	2.27
±SD			1.48	0.54	1.42	1.48	0.0098	3.50	0.46	3.03	2.04

^a^ Largest tumor diameter (cm).

**Table 3:** Statistical significances expressed as *p*-values for comparisons of biophysical tumor characteristics.

Groups Comparison	Weight	Length (D_max_)	Volume	Tumor Burden	Density	Surface Area	Length/Density	Surface/Density	Volume/Density
CO/CH	0.050489	0.002531 ^a^	0.052985	0.255392	0.830979	0.195459	0.001369 ^a^	0.178069	0.046721 ^a^
CO/IV	0.205111	0.005675 ^a^	0.241235	0.393862	0.309337	0.143891	0.003474 ^a^	0.144095	0.243922
CO/CHIV	0.000002 ^a^	0.000001 ^a^	0.000001 ^a^	0.000001 ^a^	0.000001 ^a^	0.000001 ^a^	0.000001 ^a^	0.000000 ^a^	0.000001 ^a^
CO/CHIVDA	0.433944	0.069235	0.533818	0.543198	0.248971	0.408515	0.093676	0.477941	0.603809
CO/½CHIV	0.000001 ^a^	0.000028 ^a^	0.000001 ^a^	0.000001 ^a^	0.000001 ^a^	0.000001 ^a^	0.000047 ^a^	0.000000 ^a^	0.000001 ^a^
CO/¼CHIV	0.003412 ^a^	0.004299 ^a^	0.006764 ^a^	0.001807 ^a^	0.000805 ^a^	0.005878 ^a^	0.005704 ^a^	0.007222 ^a^	0.044337 ^a^
CHIV/CHIVDA	0.000501 ^a^	0.000163 ^a^	0.001152 ^a^	0.000111 ^a^	0.000001 ^a^	0.000323 ^a^	0.000381 ^a^	0.000439 ^a^	0.001398 ^a^
CHIV/½CHIV	0.001715 ^a^	0.003082 ^a^	0.003650 ^a^	0.001660 ^a^	0.000026 ^a^	0.002308 ^a^	0.003692 ^a^	0.002393 ^a^	0.002989 ^a^
CHIV/¼CHIV	0.000967 ^a^	0.000825 ^a^	0.001720 ^a^	0.000912 ^a^	0.000001 ^a^	0.000311 ^a^	0.001151 ^a^	0.000294 ^a^	0.026050 ^a^
½CHIV/¼CHIV	0.003816 ^a^	0.081714	0.006752 ^a^	0.003752 ^a^	0.000001 ^a^	0.004309 ^a^	0.129894	0.004773 ^a^	0.062622
CH/CHIV	0.000029 ^a^	0.000001 ^a^	0.000028 ^a^	0.000035 ^a^	0.000001 ^a^	0.000001 ^a^	0.000001 ^a^	0.000001 ^a^	0.000025 ^a^
CH/½CHIV	0.000096 ^a^	0.003241 ^a^	0.000105 ^a^	0.000093 ^a^	0.000001 ^a^	0.000025 ^a^	0.012036 ^a^	0.000029 ^a^	0.000109 ^a^
CH/¼CHIV	0.134220	0.446129	0.187531	0.040015 ^a^	0.000030 ^a^	0.059510	0.614850	0.078267	0.391871
IV/CHIV	0.000211 ^a^	0.000036 ^a^	0.000385 ^a^	0.000367 ^a^	0.000001 ^a^	0.000071 ^a^	0.000046 ^a^	0.000001 ^a^	0.000318 ^a^
IV/½CHIV	0.000527 ^a^	0.009311 ^a^	0.001011 ^a^	0.000758 ^a^	0.000001 ^a^	0.000551 ^a^	0.023073 ^a^	0.000554 ^a^	0.000918 ^a^
IV/¼CHIV	0.104116	0.508063	0.152376	0.071325	0.012227 ^a^	0.188876	0.641004	0.215931	0.276903
CH/IV	0.660915	0.963827	0.664331	0.947747	0.290929	0.679907	1.000000	0.700565	0.632249
CHIVDA/½CHIV	0.001094 ^a^	0.009184 ^a^	0.002506 ^a^	0.000254 ^a^	0.000017 ^a^	0.001657 ^a^	0.022452 ^a^	0.002413 ^a^	0.003140 ^a^
CHIVDA/¼CHIV	0.085036	0.252713	0.124713	0.035311 ^a^	0.033504 ^a^	0.152806	0.309551	0.175412	0.209772

^a^ *p* < 0.05. CO—control group; CH—group treated with chloroquine; IV—group treated with ivermectin; CHIV—group treated with the combination of chloroquine and ivermectin; CHIVDA—group treated with the combination of chloroquine, ivermectin and deoxycholic acid; ½CHIV—group treated with 1/2 doses of chloroquine and ivermectin; ¼CHIV—group treated with 1/4 doses of chloroquine and ivermectin. All results obtained through parametric statistical methods were validated using the two-sided non-parametric Mann–Whitney U test.

**Table 4:** Statistical significances expressed as *p*-values for comparisons of immunohistochemical tumor characteristics.

Groups Comparison	p53	Ki-67	PCNA	CD34	CD31	GLUT-1	iNOS	COX4	Cytochrome C	Caspase 3
CO/CH	0.640250	0.383128	0.371571	0.868017	0.931537	0.619430	0.277847	0.860595	0.831207	0.481381
CO/IV	0.789362	0.124534	0.558146	0.960718	0.882287	0.463198	0.760268	0.734399	0.631908	0.264739
CO/CHIV	0.000000 ^a^	0.000000 ^a^	0.000000 ^a^	0.000011 ^a^	0.000001 ^a^	0.000000 ^a^	0.000278 ^a^	0.000001 ^a^	0.000000 ^a^	0.000240 ^a^
CO/CHIVDA	0.896832	0.405691	0.374129	0.987783	0.761818	0.202540	0.872385	0.568347	0.870239	0.464681
CO/½CHIV	0.000020 ^a^	0.000000 ^a^	0.000001 ^a^	0.000023 ^a^	0.000001 ^a^	0.000000 ^a^	0.001671 ^a^	0.000530 ^a^	0.000025 ^a^	0.000986 ^a^
CO/¼CHIV	0.005514 ^a^	0.001029 ^a^	0.000485 ^a^	0.013945 ^a^	0.006682 ^a^	0.000064 ^a^	0.050970	0.020983 ^a^	0.005623 ^a^	0.017715 ^a^
CHIV/CHIVDA	0.000001 ^a^	0.000000 ^a^	0.000000 ^a^	0.000001 ^a^	0.000033 ^a^	0.000000 ^a^	0.000397 ^a^	0.000052 ^a^	0.000001 ^a^	0.000001 ^a^
CHIV/½CHIV	0.000122 ^a^	0.002831 ^a^	0.000258 ^a^	0.000047 ^a^	0.000548 ^a^	0.076252	0.022123 ^a^	0.009172 ^a^	0.000409 ^a^	0.000423 ^a^
CHIV/¼CHIV	0.000000 ^a^	0.000042 ^a^	0.000001 ^a^	0.000000 ^a^	0.000000 ^a^	0.000000 ^a^	0.000176 ^a^	0.000001 ^a^	0.000001 ^a^	0.000000 ^a^
½CHIV/¼CHIV	0.000060 ^a^	0.001390 ^a^	0.002010 ^a^	0.000000 ^a^	0.000000 ^a^	0.000001 ^a^	0.005355 ^a^	0.010894 ^a^	0.006343 ^a^	0.000212 ^a^
CH/CHIV	0.000000 ^a^	0.000000 ^a^	0.000000 ^a^	0.000000 ^a^	0.000000 ^a^	0.000000 ^a^	0.000588 ^a^	0.000000 ^a^	0.000000 ^a^	0.000024 ^a^
CH/½CHIV	0.000001 ^a^	0.000016 ^a^	0.000001 ^a^	0.000000 ^a^	0.000000 ^a^	0.000001 ^a^	0.005906 ^a^	0.000067 ^a^	0.000037 ^a^	0.000184 ^a^
CH/¼CHIV	0.001931 ^a^	0.011337 ^a^	0.001181 ^a^	0.003234 ^a^	0.000844 ^a^	0.000339 ^a^	0.314943	0.003365 ^a^	0.005718 ^a^	0.013392 ^a^
IV/CHIV	0.000000 ^a^	0.000000 ^a^	0.000000 ^a^	0.000000 ^a^	0.000001 ^a^	0.000000 ^a^	0.001801 ^a^	0.000000 ^a^	0.000000 ^a^	0.000000 ^a^
IV/½CHIV	0.000019 ^a^	0.000026 ^a^	0.000021 ^a^	0.000001 ^a^	0.000016 ^a^	0.000001 ^a^	0.009362 ^a^	0.000225 ^a^	0.000300 ^a^	0.000044 ^a^
IV/¼CHIV	0.003958 ^a^	0.029560 ^a^	0.002010 ^a^	0.005092 ^a^	0.010321 ^a^	0.000600 ^a^	0.159354	0.014892 ^a^	0.014174 ^a^	0.011532 ^a^
CH/IV	0.850594	0.539873	0.806445	0.897217	0.801714	0.813520	0.512303	0.520907	0.756879	0.593333
CHIVDA/½CHIV	0.000001 ^a^	0.000000 ^a^	0.000000 ^a^	0.000018 ^a^	0.000001 ^a^	0.000001 ^a^	0.002089 ^a^	0.003024 ^a^	0.000428 ^a^	0.000060 ^a^
CHIVDA/¼CHIV	0.006862 ^a^	0.001368 ^a^	0.000450 ^a^	0.011881 ^a^	0.027496 ^a^	0.000701 ^a^	0.047976 ^a^	0.108025	0.023010 ^a^	0.006774 ^a^

^a^ *p* < 0.05. CO—control group; CH—group treated with chloroquine; IV—group treated with ivermectin; CHIV—group treated with the combination of chloroquine and ivermectin; CHIVDA—group treated with the combination of chloroquine, ivermectin and deoxycholic acid; ½CHIV—group treated with 1/2 doses of chloroquine and ivermectin; ¼CHIV—group treated with 1/4 doses of chloroquine and ivermectin.

**Table 5:** In vitro evaluated half maximal inhibitory concentrations (IC_50_), with confidence intervals and selectivity index (SI) of examined drugs on different cell cultures after 48 h exposure.

Drug	Normal Fetal Lung MRC-5	Lung Carcinoma A549		Colon Carcinoma HT-29		Cervix Carcinoma HeLa		Hamster Fibrosarcoma BHK-21/C13	
	IC_50_	IC_50_	SI	IC_50_	SI	IC_50_	SI	IC_50_	SI
Chloroquine (μM)	40 ± 2.35	23 ± 0.90	1.7	115 ± 6.76	0.3	45 ± 3.53	0.9	63 ± 3.70	0.6
Ivermectin (μM)	5.1 ± 0.20	5.6 ± 0.33	0.9	3.22 ± 0.19	1.6	6.6 ± 0.39	0.8	9.6 ± 0.47	0.5
Chloroquine + ivermectin 10:1 (μM)	39 ± 2.29	12 ± 0.82	3.3	6.3 ± 0.43	6.2	24 ± 1.65	1.6	19 ± 1.49	2.1

## Data Availability

The original contributions presented in this study are included in the article/Supplementary Materials. Further inquiries can be directed to the corresponding author.

## Supplementary Materials

The following supporting information can be downloaded at: https://www.mdpi.com/article/10.3390/ph19030407/s1. Introduction—Supplementary Table S1: Separate anticancer effects of chloroquine and ivermectin vs. reciprocal deoxycholic acid modulation of the same established oncological targets/markers in different studies (detailed review); Experimental animals; BHK-21/C13 cell culture; Histological staining procedures; Blood analyses; Results—Supplementary Table S2: Blood analysis for the first four experimental groups.
