# Supplementary material for: Captivating Synergistic, Dose-Dependent Anticancer Effects of Tumor-Regulation Modulators Chloroquine and Ivermectin Completely Abolished by an Opposing Modulator, Deoxycholic Acid, in Hamster Fibrosarcoma: In Vivo, In Vitro, and Literature Review
Source: Pharmaceuticals (Basel). 2026 Mar 1;19(3):407. doi: 10.3390/ph19030407 (PMC13028768; doi:10.3390/ph19030407)
Supplement: Supplementary file 1 [file pharmaceuticals-19-00407-s001.zip › pharmaceuticals-4158985-supplementary.pdf]

pharmaceuticals

MDPI

Article

Captivating Synergistic, Dose-Dependent Anticancer Effects of Tumor-Regulation Modulators Chloroquine and Ivermectin Completely Abolished by an Opposing Modulator, Deoxycholic Acid, in Hamster Fibrosarcoma: In Vivo, In Vitro, and Literature Review

Kosta J. Popović [1], Dušica J. Popović [2], Dejan Miljković [3], Jovan K. Popović [4,5,*], Mihalj Poša [1], Jovana Drljača Lero [1] and Zana Dolićanin [2]

[1] Department of Pharmacy, Faculty of Medicine, University of Novi Sad, Hajduk Veljkova 3, 21000 Novi Sad, Serbia
[2] Department of Biomedical Sciences, State University of Novi Pazar, Vuka Karadžića 9, 36300 Novi Pazar, Serbia
[3] Department of Histology and Embryology, Faculty of Medicine, University of Novi Sad, Hajduk Veljkova 3, 21000 Novi Sad, Serbia
[4] Department of Pharmacology, Toxicology and Clinical Pharmacology, Faculty of Medicine, University of Novi Sad, Hajduk Veljkova 3, 21000 Novi Sad, Serbia
[5] Academy of Medical Sciences of the Serbian Medical Society, 19 George Washington Str., 11000 Belgrade, Serbia
* Correspondence: jovapopmf@gmail.com

Supplementary Data:

The following are Supplementary data to this article:

 https://doi.org/10.3390/ph19030407

Introduction

Supplemental Table S1. Separate anticancer effects of chloroquine and ivermectin vs. reciprocal deoxycholic acid modulation of the same established oncological targets/markers in different studies (detailed review).

Abbreviations: tumor promoter **(P)**, tumor suppressor **(S)**. Symbols used for effects on other targets: stimulation (/), inhibition (\).

**Anticancer therapy targets/ markers, pathways and roles**	**Chloroquine**				**Ivermectin**				**Deoxycholic acid**			
	**Cells**	***In vitro/ In vivo***	**Effect**	**Ref. No.**	**Cells**	***In vitro/ In vivo***	**Effect**	**Ref. No.**	**Cells**	***In vitro/ In vivo***	**Effect**	**Ref. No.**
**1) Transcription factors:**												
**NF-κB** [a] **(P)** - tumor promoter	T-cell leukemia / lymphoma MOLT4, JURKAT, HCT1, HCT4, HCT5	*In vitro*	Inhibition	[2]	inflammatory cells	*In vivo* rats	Inhibition	[3]	HCT-116 colon epithelial cells	*In vitro*	Stimulation	[5]
					macrophages	*In vivo* mice	Inhibition	[4]	Barrett's epithelial cells BAR-T, BAR-T10	*In vitro* and *In vivo* patients	Stimulation	[6]
[a] **NF-κB**: 1. NF-κB1 (p105/p50); 2. NF-κB2 (p100/p52); 3. p65 (RelA); 4. V-Rel reticuloendotheliosis viral oncogene homolog B (RelB); 5. cRel IκB (IKK)/NF-κB/STAT3\apoptosis; EGFR/RAS/Raf/p38/MAPK/MEK/ERK/Akt/mTOR/NF-κB/P-gp (MDPR); PI3K/Akt/JNK/IκBα/NF-κB/P-gp; TGF-α/PI3K/Akt/mTOR/HIF/IκBα/NF-κB/HIF/VEGF; PI3K/PKC/NF-κB …; STAT3/PI3K/Akt /…/NF-κB …; p38/MAPK/PI3K/Akt/…/NF-κB …; p38/MAPK/JNK/ERK/Akt/IκBα/NF-κB …; TNF/IκB (IKKα, IKKβ, IKKγ = NEMO)/NF-κB …; IL1/IκB/NF-κB …; TLRs/IκB/NF-κB …; TLRs/IL1/IκB/NF-κB …; TLR4/TNF-α, IL1β/IκB/NF-κB …; TLRs/p38/MAPK/PI3K/Akt/…/NF-κB …; TLRs/p38/MAPK/ERK/Akt/IκBα/NF-κB …; PANX1/ILs/NF-κB …; P2X4, P2X7/ROS/…/NF-κB												
**p65** (Rel A) **(P)**	human: corneal epithelial and fibroblasts cells	*In vitro*	Inhibition	[7]	pulmonary inflammatory	*In vivo* rats	Inhibition	[8]	Barrett's epithelial cells BAR-T, BAR-T10	*In vitro* and *In vivo* patients	Stimulation	[6]
**Integrins (P)** Integrin β1 is NF-κB	human: melanoma WM793, 1205Lu	*In vitro*	Inhibition	[9]	human: colorectal HCT-8, HCT-116; and breast MCF-7, MDA-MB-231 cancer	*In vitro* and *In vivo* mice	Inhibition	[10]	oesophageal cells OE33	*In vitro*	Stimulation	[11]
**STATs** [b] **(P)**, 7 proteins	human: osteosarcoma 143B, U-2OS	*In vitro* and *In vivo* mice	Inhibition	[12]	virus-infected inflammation human cells	*In vitro/ In vivo (review)*	Inhibition	[13]	human gastric cells and cancer cells AGS, SGC-7901, BGC-823, MKN-45	*In vitro* and *In vivo* mice, humans	Stimulation	[16]
						In vitro/ In vivo (review)	Inhibition	[14], [15]				
[b] **STATs**: JAK / STAT / PI3K / Akt / mTOR / NF-κB; JAK / STAT / MAPK / ERK / NF-κB; KFL4, JAK2, Oct4 / STAT; STAT3 / Mcl1, Bclx, C-MYC; NF-κB / STAT3 / NF-κB; STAT3 / NMI / MYC (N-MYC, C-MYC) / NF-κB.												
**Nrf2 (S)** - tumor suppressor Nrf2 \ NF-κB	skeletal muscle	*In vivo* human muscle biopsies	Stimulation	[17]	human: colorectal HCT-8, breast MCF-7 cancer and leukemia K562	*In vitro* and *In vivo* mice	Modulation	[18]	mice: jejunal and intestinal epithelial	*In vitro* and *In vivo* mice	Modulation	[19]
**Nanog, KFL4, Sox2, Oct4 (P)** Nanog, KFL4, Sox2, Oct4 / STATs/ NF-κB; EGFR/PI3K/Akt/TGF-α/ Nanog, KFL4, Sox2, Oct4; \ IκBα / p65, p50, NF-κB / IL6, TNF-α /	human: esophageal squamous cell carcinoma EC109	*In vivo* human surgical resection	Inhibition	[20]	breast MDA‑MB‑31 cancer cell line	*In vitro*	Inhibition	[21]	human: esophageal adenocarcinoma EAC, OE33, and normal esophageal HEEC	*In vitro*	Stimulation	[22]

Abbreviations: tumor promoter **(P)**, tumor suppressor **(S)**. Symbols used for effects on other targets: stimulation (/), inhibition (\).

**Anticancer therapy targets/ markers, pathways and roles**	**Chloroquine**				**Ivermectin**				**Deoxycholic acid**			
	**Cells**	***In vitro/ In vivo***	**Effect**	**Ref. No.**	**Cells**	***In vitro/ In vivo***	**Effect**	**Ref. No.**	**Cells**	***In vitro/ In vivo***	**Effect**	**Ref. No.**
/ Nanog, KFL4, Sox2, Oct4.									mice: hepatocellular carcinoma	*In vitro* and *In vivo* mice	Stimulation	[23]
**JNKs (P)** belong to p38/MAPK<br>RAS / Raf / p38 / MAPK / MEK /<br>/ ERK / Akt / JNK / IκBα / NF-κB.	mice: macrophages	*In vitro* and *In vivo* mice	Inhibition	[24]	lung injury inflammatory cells	*In vivo* mice	Inhibition	[25]	rat: primary hepatocytes	*In vitro*	Stimulation	[26]
**HES1, K13, MUC2 (P)**<br>Notch1, Hedgehog, Wnt, VEGF /<br>/ HES1 / NF-κB;<br>K13 / NF-κB;<br>NF-κB/MUC2/Wnt/β-catenin, Integrin β1 /<br>/ FAK / ERK.	mice: psoriatic skin	*In vivo* mice	Modulation	[27]		*In vitro/ In vivo (review)*	Modulation	[28]	human esophageal: epithelial Het-1A and surgical specimens normal, metaplastic	*In vitro* and *In vivo*	Modulation	[30]
					zebrafish: larvae	*In vivo* zebrafish	Modulation	[29]				
**VDR (S)** vitamin D receptor<br>VDR \ IκB \ NF-κB	breast carcinoma	*In vitro*	Inhibition	[31]		*In vitro/ In vivo (review)*	Inhibition	[32]	mice: myelopoietic	*In vitro* and *In vivo*	Stimulation	[33]
**Hippo \ YAP** [c] **(P)**	human: umbilical vein endothelial	*In vitro*	Inhibition	[34]	LMob1DKO liver and Mob1a/1b deficient mice hepatocyte cells	*In vitro/ In vivo* Mob1a/1b deficient mice	Inhibition	[36]	mice hepatocytes	*In vitro/ In vivo* mice	Stimulation	[37]
	mice: gastric carcinoma	*In vivo* mice	Inhibition	[35]								
[c] **Hippo:** Hippo / NF-κB, HIF2α / NF-κB; YAP \ NF-κB, p65 / NF-κB; \ Hippo / YAP \ p65 / NF-κB; Hippo \ YAP / HIF2α / NF-κB.												
**TCFs, LEFs (P)**:<br>Wnt/β-catenin/TCFs,LEFs/Cyclin D_1/NF-κB;<br>Wnt / TCFs \ NF-κB.	human: nonsmall lung adeno-carcinoma A549	*In vitro*	Modulation	[38]	human: colon, lung non-small, bronchio-alveolar, glioma, melanoma cancer cells*[1] →	*In vitro**[1] CC14, CC36, Ls174T, HT29, DLD1, H358, 293T, U251, SKMel2 and *In vivo* mice	Inhibition	[40]	colon cancer SW480, LoVo	*In vitro*	Stimulation	[41]
		In vitro/ In vivo (review)	Modulation	[39]					oesophageal	*In vitro*	Stimulation	[42]
**2) Cell cycle proteins:**												
**Cyclin D_1, CDKs (P)**<br>Cyclin D_1 / CDK4, CDK6, NF-κB, VEGF;<br>NF-κB / Cyclin D_1; CDK7 / β-catenin / TCF;<br>β-catenin /TCF / Cyclin D_1 / CDKs /NF-κB<br>Cyclin D_1/CDK4/CDK2\p21,p27(Cyp/Kip-CDK); Cyclin D_1 \ Fas.	rats: hippocampus glioma	*In vivo* rats	Inhibition	[43]	human: colon, lung non-small, bronchio-alveolar, glioma, melanoma cancer cells*[2] →	*In vitro**[2] CC14, CC36, Ls174T, HT29, DLD1, H358, 293T, U251, SKMel2 and *In vivo* mice	Inhibition	[40]		*In vitro/ In vivo (review)*	Modulation	[45]
	colon cancer DLD-1	*In vitro*	Inhibition	[44]								
**p21, p27 (S)**<br>p21, p27 \ CDK;<br>CDK / NF-κB;	epithelial ovarian cancer A2780-CP20, RMG-1	*In vitro* and *In vivo* mice	Stimulation	[46]	human: urothelial carcinoma T24, RT4	*In vitro*	Stimulation	[48]	primary mouse hepatocytes	*In vitro*	Inhibition	[50]

Abbreviations: tumor promoter **(P)**, tumor suppressor **(S)**. Symbols used for effects on other targets: stimulation (/), inhibition (\).

**Anticancer therapy targets/ markers, pathways and roles**	**Chloroquine**				**Ivermectin**				**Deoxycholic acid**			
	**Cells**	***In vitro/ In vivo***	**Effect**	**Ref. No.**	**Cells**	***In vitro/ In vivo***	**Effect**	**Ref. No.**	**Cells**	***In vitro/ In vivo***	**Effect**	**Ref. No.**
p21, p27 \ NF-κB;<br>p21, p27 \ CDK / NF-κB.	colorectal cancer HT-29	*In vitro*	Stimulation	[47]	human: liver SKOV3, OVCAR, CAOV3, pancreatic breast and liver cancer cells*3→	*In vitro**3 MDA-MB231, MCF-7, HEK-293, MIA PaCa-2, Huh-7 and *In vivo* mice	Stimulation	[49]	mice hepatocytes CD95	*In vitro/*	Modulation	[51]
**Ki-67 (P)**<br>Ki-67 \ p21;<br>NF-κB / Ki-67.	glioblastoma stem cells	*In vitro* and *In vivo* mice	Inhibition	[52]	esophageal normal epithelial and squamous carcinoma cells NE-3, KYSE-70, KYSE-30	*In vitro* and *In vivo* mice	Inhibition	[54]	mice colonic epithelium and human colorectal cancer HCT-116	*In vitro and In vivo* mice	Stimulation	[55]
		In vitro/ In vivo (review)	Inhibition	[53]								
**PCNA (P)**<br>PCNA \ p21;<br>PCNA / p38MAPK / JNK / ERK / PI3K / / Akt / mTOR / IκBα / NF-κB / P-gp.	rats: hippocampus glioma	*In vivo* rats	Inhibition	[43]	inflammatory cells	*In vivo rats*	Inhibition	[3]	human: colorectal adenoma biopsy specimens	*In vivo* human colorectal adenoma biopsy specimens	Stimulation	[56]
**Akt** [d] **(P)**, Akt = PKB	human: lung A549 and kidney 293T	*In vitro*	Inhibition	[57]	human: colorectal, breast cancer and chronic myeloid leukemia cells*4→	*In vitro**4 HCT-8, MCF-7, K562 and *In vivo* mice	Inhibition	[18]	human: gastric cancer SNU601	*In vitro*	Stimulation	[58]
[d] **Akt**: JAK/STAT3/PI3K/Akt/mTOR/NF-κB; ERK/Akt/NF-κB/P-gp; PTEN\Akt; PTEN\PI3K/Akt; PTEN\JAK/STAT3; PTEN\JAK/STAT3/PI3K/Akt/mTOR/IκBα/NF-κB; PTEN\Raf/MEK/ERK/PTEN/Akt/JNK/IκBα/NF-κB; TGF-α/PI3K/Akt; Akt/iNOS, VEGF, HIF-1α, HIF-2α, mTOR, NF-κB; PI3K/Akt/mTOR/HIFs/VEGF, NF-κB; EGFR/Raf/RAS/p38 MAPK/MEK/ERK/Akt/mTOR/IκBα/NF-κB/P-gp; Akt/JNK/IκBα/NF-κB/P-gp; Akt/mTOR/HIF/IκBα/NF-κB/HIF/VEGF.												
**mTOR (P)**<br>Akt / mTOR / NF-κB;<br>\ mTOR \ NF-κB;<br>TGF-α/PI3K/Akt/mTOR/IκB/NF-κB/P-gp (MDR1-ABCB1);<br>IGF-1, IGF-2 / mTOR / IκB / NF-κB / P-gp.	human: lung A549 and cervical HeLa cancer	*In vitro*	Inhibition	[59]		*In vitro/ In vivo (review)*	Inhibition	[60]	human: hepatoma SK-Hep-1 cell line HTB-52	*In vitro*	Stimulation	[61]
**EGFR (P)**, of RTKs<br>EGFR/PI3K/Akt/mTOR/IκBα/NF-κB/P-gp (P-gp = MDR1 = ABCB1);<br>EGFR/Raf/RAS/p38 (MAPK)/MEK/ERK/ /Akt/JNK,mTOR/IκBα/NF-κB/P-gp.	human: breast cancer MDA-MB-231, MCF-7, HCC1937 and MDA-MB-453	*In vitro* and *In vivo* mice	Inhibition	[62]	human: colorectal HCT-8, breast MCF-7 cancer and myeloid leukemia K562	*In vitro* and *In vivo* mice	Inhibition	[18]		*In vitro/ In vivo (review)*	Stimulation	[63]
**Raf, RAS,** MAPK **(P)**<br>EGFR/Raf-1/RAS/p38MAPK/MEK/ERK/ /Akt/mTOR, JNK/IκBα/NF-κB/P-gp.	human: H322C, HCC4006, Calu3, H358, A549, H2009, NSCLC, HOSE	*In vitro*	Inhibition	[64]		*In vitro/ In vivo (review)*	Inhibition	[60]	rat: primary hepatocytes	*In vitro*	Stimulation	[26]

Abbreviations: tumor promoter **(P)**, tumor suppressor **(S)**. Symbols used for effects on other targets: stimulation (/), inhibition (\).

**Anticancer therapy targets/ markers, pathways and roles**	**Chloroquine**				**Ivermectin**				**Deoxycholic acid**			
	**Cells**	***In vitro/ In vivo***	**Effect**	**Ref. No.**	**Cells**	***In vitro/ In vivo***	**Effect**	**Ref. No.**	**Cells**	***In vitro/ In vivo***	**Effect**	**Ref. No.**
		In vitro/ In vivo (review)	Inhibition	[65]								
**PI3K[c] (P)**	human: lung A549 and kidney 293T	*In vitro*	Inhibition	[57]	human: neuroblastoma SH-SY5Y	*In vitro*	Inhibition	[66]	rats: hepatoma McNtcp.24	*In vitro*	Stimulation	[67]
									human: colon epithelial and carcinoma HT-29	*In vitro*	Stimulation	[68]
[c] **PI3K**: TGF-α, STAT3, p38 MAPK, MEK, ERK, EGFR, Raf, RAS / PI3K / Akt, mTOR, JNK, PKC, p38 MAPK / IκBα / NF-κB / HIF / VEGF; PTEN \ PI3K; PI3K, mTOR \ p21, p27; PI3K / autophagy.												
**3) Cell signaling cascades proteins:**												
**MDR (P)**, (MDPRs, MRP1-7, ABC transporters, ATP binding cassettes, P-gp tyrosine kinase). EGFR, Raf, RAS, p38 (MAPK), MEK, ERK, Akt, JNK, IGF, mTOR, TGF-α, STAT3, PI3K, PKC / IκBα / NF-κB / / MDR (MDPRs, ABC transporters), P-gp		*In vitro/ In vivo (review)*	Inhibition	[69]		*In vitro/ In vivo (review)*	Inhibition	[60]	human: colon adeno-carcino ma Caco2	*In vitro*	Stimulation	[70]
									mice: myelopoietic	*In vitro* and *In vivo*	Stimulation	[71]
**p65** (NF-κB subunit): see **1) Transcription factors**												
**IκB (P)** IκB = IKK = κKα + IκKβ + IκKγ (NEMO). IκB / NF-κB.	human: T-cell acute lymphoblastic leukemia / lymphoma cells*[5]→	*In vitro**[5] Su9T01, S1T, MT1, MOLT4, JURKAT, HCT1, HCT4, HCT5 and *In vivo* mice	Inhibition	[2]	esophageal normal epithelial and squamous carcinoma cells NE-3, KYSE-70, KYSE-30	*In vitro* and *In vivo* mice	Inhibition	[54]	Barrett's epithelial cells BAR-T, BAR-T10	*In vitro* and *In vivo* patients	Stimulation	[6]
**Hedgehog (P)** NF-κB/Hedgehog/NF-κB, Cyclin D1, SMO; Hedgehog \ Fas.	human: pancreatic cancer stem cells, Panc1, BxPC3, 8988 T	*In vitro* and *In vivo* mice	Inhibition	[72]		*In vitro/ In vivo (review)*	Inhibition	[15]	human: esophageal adeno-carcino ma EAC, OE33, and normal HEEC	*In vitro*	Stimulation	[22]
**ABC (P)**, eflux transporters, ATP-binding cassette. ABC members: P-gp, MRP1 are responsible for cancer resistance. ABC / NF-κB / ABC; JNK / Akt / NF-κB / P-gp.		*In vitro/ In vivo (review)*	Inhibition	[69]		*In vivo* nematode	Inhibition	[73]	human: breast cancer MCF-7, small cell lung cancer H69, $H69_{AR}$, endothelial kidney HEK293, $HEK293_{MRP1}$	*In vitro*	Modulation	[75]
					human: lung cancer A549, HL60-MRP1	*In vitro*	Inhibition	[74]				

Abbreviations: tumor promoter **(P)**, tumor suppressor **(S)**. Symbols used for effects on other targets: stimulation (/), inhibition (\).

**Anticancer therapy targets/ markers, pathways and roles**	**Chloroquine**				**Ivermectin**				**Deoxycholic acid**			
	**Cells**	***In vitro/ In vivo***	**Effect**	**Ref. No.**	**Cells**	***In vitro/ In vivo***	**Effect**	**Ref. No.**	**Cells**	***In vitro/ In vivo***	**Effect**	**Ref. No.**
**PKC [f] (P, S)**		*In vitro P. falciparum*	Inhibition	[76]	tissue extracts of the filarial worms *Brugia pahangi* and *Onchocerca gibsoni*	*In vivo* filarial worms tissue extracts	Inhibition	[77]	human: squamous oesophageal epithelial HET-1A, metaplastic and dysplastic Barrett's cells QH and GO	*In vitro*	Stimulation	[78]
[f] **PKC (P, S).** **PKC (P)**: PTK / PKC / NF-κB; PKC / Raf / MEK / ERK / Akt / IκBα / NF-κB / HIF / VEGF; PKC \ PTEN; PKC / PI3K, Cyclin D1, Wnt / β-catenin; **PKC (S)**: PKC / $p21^{Cyp1}$; PKC \ C-MYC, RAS, IL6.												
**JAKs (P)**, tyrosine kinase of NRTKs. JAK / STAT / NF-κB.	human: breast cancer stem Hs578t, MDA-MB-231, HCC1937, HCC38, SUM159PT	*In vitro* and *In vivo* mice	Inhibition	[79]	glioma U87 cells	*In vitro* and *In vivo* rats	Inhibition	[81]	human: esophageal adenocarcinoma EAC, OE33, and normal esophageal HEEC	*In vitro*	Stimulation	[22]
		In vitro/ In vivo (review)	Inhibition	[80]								
**Wnt / β-catenin (P)**: Wnt / β-catenin / TCF / Cyclin D1 / NF-κB; Wnt / β-catenin \ autophagy.		*In vitro/ In vivo (review)*	Inhibition	[82]		*In vitro/ In vivo (review)*	Inhibition	[82]	human: colon cancer SW480, LoVo	*In vitro*	Stimulation	[41]
	gastric GES-1	*In vitro*	Inhibition	[35]								
**ILs (P)**: IL1, IL1β, IL6, IL8, IL17 / NF-κB; NF-κB / IL6; IL10 \ NF-κB; TLR, p38MAPK, JNK, ERK, FAK (PTK2), TGF-β1 / ILs.	human: monocytes/ macrophages U-937, THP-1	*In vitro*	Inhibition	[83]	human: epidermal keratinocytes	*Ex vivo*	Inhibition	[84]	human: colorectal cancer HT-29	*In vitro*	Stimulation	[86]
					mice: psoriatic skin	*In vivo* mice	Inhibition	[85]	human: squamous oesophageal epithelial HET-1A, metaplastic and dysplastic Barrett's cells QH and GO	*In vitro*	Stimulation	[78]
**IGF-1, IGF-2** (IGF-1R, IGF-2R) **(P):** IGF / mTOR / IκB / NF-κB / P-gp	opossum: kidney cell line	*In vitro*	Inhibition	[87]	lambs: serum	*In vivo* lambs	Inhibition	[88]	colon cancer HCT116	*In vitro*	Stimulation	[89]
**PTEN (S)**: PTEN \ NF-κB; PTEN \ PI3K / Akt / mTOR / NF-κB; PTEN\MEK/ERK/Akt/JNK/IκBα/NF-κB; STAT3, Raf \ PTEN.		*In vitro/ In vivo (review)*	Stimulation	[80]	carps: neutrophils	*In vivo* carps	Stimulation	[90]	human: gastric normal epithelial and cancer cells*[6]→	*In vitro*[*6] GES-1, AGS, AZ-521, BGC823, SGC7901, MKN45	Modulation	[91]

Abbreviations: tumor promoter **(P)**, tumor suppressor **(S)**. Symbols used for effects on other targets: stimulation (/), inhibition (\).

**Anticancer therapy targets/ markers, pathways and roles**	**Chloroquine**				**Ivermectin**				**Deoxycholic acid**			
	**Cells**	***In vitro/ In vivo***	**Effect**	**Ref. No.**	**Cells**	***In vitro/ In vivo***	**Effect**	**Ref. No.**	**Cells**	***In vitro/ In vivo***	**Effect**	**Ref. No.**
									human: colon carcinoma HCT116, DLD1, HCT15, HT29, SW480	*In vitro*	Inhibition	[92]
**Eps8 (P)**, a part of EGFR: Eps8 / EGFR, FGFR, PDGFR, ErbB-2, ILs, PKC, RAS, MAPK, FAK (=PTK2), NF-κB; Eps8/EGFR/RAS/Raf/MAPK/MEK/ERK/... …/ NF-κB; Eps8 / EGFR / ErbB-2 / NF-κB.		*In vivo Plasmodium falciparum*	Inhibition	[93]	human: chronic myeloid leukemia K562, CD34 and normal bone marrow CD34	*In vitro* and *In vivo*	Inhibition	[94]	human: squamous oesophageal epithelial HET-1A, metaplastic and dysplastic Barrett's cells QH and GO	*In vitro*	Stimulation	[78]
					human: colorectal HCT-8, HCT-116 and breast MCF-7, MDA-MB-231 cancer	*In vitro* and *In vivo* mice	Inhibition	[10]	human: rectal carcinoma HCA-7	*In vitro*	Modulation	[95]
**4) Apoptosis and/or necroptosis associated proteins:**												
**Bcl-2 (P)**: Bcl-2 \ apoptosis, p53; STAT3, NF-κB / Bcl-2 / NF-κB.	human: lung A549 and kidney 293T	*In vitro*	Inhibition	[57]		*In vitro/ In vivo (review)*	Inhibition	[15]	human: colon tumor derived line HCT116	*In vitro*	Stimulation	[97]
	mice: telencephalic neuronal cultures	*In vitro*	Inhibition	[96]								
**Bax (S)**: Bax / apoptosis; Bax \ NF-κB / Bax.	human: lung A549 and kidney 293T	*In vitro*	Stimulation	[57]		*In vitro/ In vivo (review)*	Stimulation	[15]	human: colon cancer HCT116	*In vitro*	Inhibition	[98]
**Cytochrome C (S, P)**: Cytochrome C **(S)**: Cytochrome C / caspase / apoptosis. Cytochrome C **(P)**: Cytochrome C / NF-κB; NF-κB \ Cytochrome C.	human: pancreatic cancer Panc-1, colon cancer HCT-116	*In vitro*	Modulation	[99]	human: oesophageal epithelial and squamous cell carcinoma lines*7→	*In vitro**7 Het-1A, EC109, KYSE70, KYSE150, KYSE30,	Modulation	[100]		*In vitro/ In vivo (review)*	Modulation	[45]
**PARPs (P)**: PARP / NF-κB (p50, p65).	human: hepatoblastoma HUH6, HB-284, HB-282,HB-303, HB-243, HB-295, HB-279	*In vitro*	Inhibition	[101]	human: cervical carcinoma HeLa	*In vitro*	Inhibition	[102]		*In vitro/ In vivo (review)*	Modulation	[103]

Abbreviations: tumor promoter **(P)**, tumor suppressor **(S)**. Symbols used for effects on other targets: stimulation (/), inhibition (\).

**Anticancer therapy targets/ markers, pathways and roles**	**Chloroquine**				**Ivermectin**				**Deoxycholic acid**			
	**Cells**	***In vitro/ In vivo***	**Effect**	**Ref. No.**	**Cells**	***In vitro/ In vivo***	**Effect**	**Ref. No.**	**Cells**	***In vitro/ In vivo***	**Effect**	**Ref. No.**
**p53 (S)**:<br>TNF-α / p53 / apoptosis;<br>p53 \ NF-κB \ p53.		*In vitro/ In vivo (review)*	Modulation	[104]	human: breast cancer MDA-MB-231, MDA-MB-468, MCF-7, ovarian cancer SKOV-3	*In vitro* and *In vivo* mice	Inhibition	[105]	human: colon tumor HCT116	*In vitro*	Inhibition	[106]
**p63 (S)**:<br>p63 (isoform ΔNp63α)\p65 (RelA - NF-κB subunit), NF-κB \ p63 (ΔNp63α);<br>p53 mutant \ p63.		*In vitro/ In vivo (review)*	Modulation	[107]	glioma	*In vitro* and *In vivo*	Stimulation	[108]	human: normal and esophageal squamous carcinoma TE-1, TE-13	*In vitro*	Inhibition	[109]
**p73 (P, S)**:<br>p73 / apoptosis;<br>TNF-α/p73 (isoform), cABL / NF-κB (cRel);<br>p53 mutant \ p73;<br>p73 (isoforms) \ p53 (wild-type), apoptosis.	human: monocytes/ macrophages U-937, THP-1	*In vitro*	Inhibition	[83]	mice: psoriatic skin	*In vivo* mice	Inhibition	[85]	mice: monocyte/ macrophage raw 264.7, Kupffer cells; human: nontumor hepatic LO2	*In vitro* and *In vivo* mice	Stimulation	[110]
**caspase 3, 7, 8, 9, 10, 12 (S)**:<br>EGFR / RAS / Raf / MAPK / MEK / ERK / / PI3K / Akt / MAPK \ caspase 3, 8, 9, apoptosis;<br>p53 / caspase 3 / apoptosis;<br>Bax, ROS / caspase 3, 7 / apoptosis;<br>Bcl-2 \ caspase 3, 7, apoptosis;<br>caspase \ NF-κB;<br>caspase 8, 10 / NF-κB.	osteosarcoma	*In vitro* and *In vivo* mice	Stimulation	[12]	human: colorectal cancer SW480, SW1116	*In vitro*	Stimulation	[112]	hepatocytes human, rats and mice C57/BL6	*In vitro*	Inhibition	[115]
	human: glioma NCE-G22, G44, G62, G63, G84, G112, G120, G121, G130, G168, G260	*In vitro* and *In vivo* mice	Stimulation	[111]	human: urothelial carcinoma T24, RT4	*In vitro*	Stimulation	[48]				
					human: ovarian cancer Ovcar4, Ovsaho, Ovcar-8, Fuov-1, Cov-318, Cov-362	*In vitro*	Stimulation	[113]				
					glioma	*In vitro* and *In vivo*	Stimulation	[108]				
					human: lung and prostate cancer, colon carcinoma, breast adenocarcinoma, immortalized keratinocytes cell lines*[8]→	*In vitro* *[8] A549, PC3, HCT-116, MDA-MB-231, HaCaT	Stimulation	[114]				

Abbreviations: tumor promoter **(P)**, tumor suppressor **(S)**. Symbols used for effects on other targets: stimulation (/), inhibition (\).

**Anticancer therapy targets/ markers, pathways and roles**	**Chloroquine**				**Ivermectin**				**Deoxycholic acid**			
	**Cells**	***In vitro/ In vivo***	**Effect**	**Ref. No.**	**Cells**	***In vitro/ In vivo***	**Effect**	**Ref. No.**	**Cells**	***In vitro/ In vivo***	**Effect**	**Ref. No.**
**p38MAPK** (MAPKs, JNKs, ERKs) **(P, S)**:<br>EGFR / p38MAPK / JNK / ERK / Akt / / IκB / NF-κB / P-gp;<br>RAS/Raf/p38MAPK/MEK/ERK/NF-κB;<br>Raf / MEK 1, 2 / ERK 1, 2 \ p38MAPK;<br>p38 / JNK;<br>MEK 1-4 / MEK 3, 6 / p38;<br>MEK 1-4 / MEK 7 / JNKs;<br>Raf / MEK / ERK \ p38MAPK;<br>p38MAPK / ERK.	Kaposi's and Epstein-Barr + and - lymphoma BJAB, BCBL1, P3HR-1 and B-lymphoblastoid B95.8	*In vitro*	Inhibition	[116]	lung injury inflammatory cells	*In vivo* mice	Inhibition	[25]	human: esophageal epithelial	*In vitro*	Stimulation	[118]
	rats: liver	*In vivo* rats	Inhibition	[117]					human: esophageal tumor SKGT-4	*In vitro*	Stimulation	[119]
**Mcl-1 (P)**:<br>Mcl-1 (∊ Bcl) \ apoptosis;<br>STAT3, NF-κB / Bcl-2 / Mcl-1 / NF-κB.	human: lymphoblast K562	*In vitro*	Inhibition	[120]	human: pancreatic cancer MIA PaCa-2, PANC-1	*In vitro* and *In vivo* mice	Inhibition	[121]	human: hepatocellular carcinoma HepG2, BeL-7402, Huh7 and normal liver L02	*In vitro*	Stimulation	[122]
**p65** (NF-κB subunit):<br>see **1) Transcription factors**												
**Nrf2** (inhibits NF-κB):<br>see **1) Transcription factors**												
**5) Death receptors on the cell surface:**												
**FAS (S)**:<br>FAS (∊ TNF) / apoptosis;<br>FAS (FAF1) \ NF-κB.	human: monocyte-like THP-1, cervical carcinoma HeLa; murine: macrophage RAW264.7, AMJ2C-8	*In vitro* human and murine and *In vivo* human	Stimulation	[123]		*In vitro/ In vivo (review)*	Stimulation	[15]	rat: primary hepatocytes	*In vitro*	Stimulation	[26]
									hepatocytes human, rats and mice C57/BL6	*In vitro*	Modulation	[115]
**TRAIL (S)**:<br>TRAIL / TNF / apoptosis.	human: renal carcinoma, normal mesothelial, mice: kidney cell lines*[9]→	*In vitro**[9] human Caki MDA-MB-361, U251MG, NHMC, and mice TMCK-1	Stimulation	[124]		*In vitro/ In vivo (review)*	Stimulation	[15]	human: hepatocellular carcinoma HuH-7	*In vitro*	Modulation	[128]
	human: pancreatic cancer MiaPaCa-2, Panc-1	*In vitro* human	Stimulation	[125]								
	rats: hepatocellular carcinoma	*In vivo* rats	Stimulation	[126]								
	human: cholangio-carcinoma QBC939	*In vitro* human	Stimulation	[127]								
**6) Autophagy (ATG) related proteins:**												

Abbreviations: tumor promoter **(P)**, tumor suppressor **(S)**. Symbols used for effects on other targets: stimulation (/), inhibition (\).

**Anticancer therapy targets/ markers, pathways and roles**	**Chloroquine**				**Ivermectin**				**Deoxycholic acid**			
	**Cells**	***In vitro/ In vivo***	**Effect**	**Ref. No.**	**Cells**	***In vitro/ In vivo***	**Effect**	**Ref. No.**	**Cells**	***In vitro/ In vivo***	**Effect**	**Ref. No.**
**PI3K** (downstream are Akt, PKC, NF-κB): see **2) Cell cycle proteins**												
**ATG (S)**: ATG (ATG5) \ NF-κB, mTOR; ATG (APG), ATG5, ATG8 (LC3) / / autophagosome / autophagy; \ mTOR, TLR4, NF-κB /ATG; PI3K / ATG8 (LC3) / autophagy; IκBα / ERK / PKC / ATG / autophagy; TNF, ROS / NF-κB, HIF1α, mTOR \ \ autophagy (ATG), p53, apoptosis; autophagy \ NF-κB; autophagy / apoptosis.	human: U2OS, HeLa, HeLa-RFP-GFP-LC3, mice: MEFs	*In vitro* and *In vivo* mice	Inhibition	[129]	lung adenocarcinoma	*In vitro*	Stimulation	[132]	human: colon normal NCM-460 and cancer HCT-116RC	*In vitro*	Stimulation	[134]
	human: glioblastoma U87	*In vitro*	Inhibition	[130]	HEK-293 (ATCC CRL-1573)	*In vitro*	Modulation	[133]	mice hepatocytes CD95	*In vitro*	Stimulation	[51]
	rats: primary cortical neurons	*In vivo* rats	Inhibition	[131]						*In vitro/ In vivo (review)*	Stimulation	[135]
	epithelial ovarian cancer A2780-CP20, RMG-1	*In vitro* and *In vivo* mice	Inhibition	[46]								
**LC3** (ATG8) **(S)**: LC3 \ NF-κB (p65 = RelA); p62 /ATG (LC3) / autophagy; MAPK / ERK / LC3 / autophagy; LC3 \ (TNF-α / NF-κB).	human: hepatocellular carcinoma HepG2, cholangio-carcinoma QBC939	*In vitro*	Inhibition	[136]	human: cervical cancer HeLa	*In vitro*	Modulation	[137]	human: epithelial HET-1A, Barrett's esophagus derived CP-A and CP-C, esophageal cancer JH-EsoAd1	*In vitro/ In vivo* patients and rats	Stimulation (acute exposure) or Inhibition (chronic exposure)	[138]
**Beclin1 (P, S)**: Beclin1 / IκB, STAT3 / NF-κB; p62 / Beclin1 / ATG8 (LC3); PI3K, PKC, ATG, autophagy; Bcl-2 \ Beclin1.	human: renal adeno-carcinoma A498, RXF393, SN12C, 769P	*In vitro*	Inhibition	[139]	human: cervical cancer HeLa	*In vitro*	Modulation	[137]	human: epithelial HET-1A, Barrett's esophagus derived CP-A and CP-C, esophageal cancer JH-EsoAd1	*In vitro/ In vivo* patients and rats	Stimulation (acute exposure) or Inhibition (chronic exposure)	[138]
**p62 (P)**: p62 / Beclin1, caspase, RAS, STAT3, TRAF6, PI3K, ERK, PKC, ATG, ATG8 (LC3), IκBα, Nrf2, NF-κB, caspase, defective autophagy; p62 / RAS / IκB / NF-κB; p62 / TRAF6 / IκB / PKC / NF-κB; p62 / caspase / NF-κB.	human: hepatocellular carcinoma HepG2, cholangio-carcinoma QBC939	*In vitro*	Inhibition	[136]	human: cervical cancer HeLa	*In vitro*	Modulation	[137]	human: epithelial HET-1A, Barrett's esophagus derived CP-A and CP-C, esophageal cancer JH-EsoAd1	*In vitro/ In vivo* patients and rats	Stimulation (acute exposure) or Inhibition (chronic exposure)	[138]

Abbreviations: tumor promoter **(P)**, tumor suppressor **(S)**. Symbols used for effects on other targets: stimulation (/), inhibition (\).

Anticancer therapy targets/ markers, pathways and roles	Chloroquine				Ivermectin				Deoxycholic acid			
	Cells	*In vitro/ In vivo*	Effect	Ref. No.	Cells	*In vitro/ In vivo*	Effect	Ref. No.	Cells	*In vitro/ In vivo*	Effect	Ref. No.
**7) Antioxidant defense proteins and other targets / markers:**												
**ROS (P, S)**:<br>Nrf2 \ ROS;<br>ROS / EGFR / p38MAPK / ERK / Akt / / JNK / IκB / NF-κB / P-gp;<br>ROS / PI3K / Akt / NF-κB;<br>ROS / p38MAPK, ERK (1, 2), JNK, PI3K, Akt, TGF-β, SMAD, ROS1 receptors (RTKs=PTKs) / NF-κB / HIFα / VEGF, P-gp (MDPR);<br>P2X4, P2X7 / ROS / … / NF-κB.	mice: bone-marrow-derived macrophage	*In vivo* mice	Inhibition	[140]	human: lung and prostate cancer, colon carcinoma, breast adeno-carcinoma, immortalized keratinocytes *[10]→	*In vitro**[10] A549, PC3, HCT-116, MDA-MB-231, HaCaT	Stimulation	[114]	human: hepatoma HuH7	*In vitro*	Stimulation	[143]
	human cancer: colorectal, breast, glioblastoma, head and neck cell lines*[11]→	*In vitro**[11] HCT116, HT29, MDA-MB-231, HCC1937, A-172, LN-18, CAL-33, 32816	Stimulation	[141]	bladder cancer cells	*In vitro* and *In vivo* mice	Stimulation	[142]	human: OE33 oesophageal adenocarcinoma	*In vitro*	Stimulation	[144]
									human: pancreatic adenocarcinoma Capan-2, BxPC-3	*In vitro*	Stimulation	[145]
**COX-2** (PTG S2) **(P)**:<br>ERK, TNF-α, p38MAPK / NF-κB / COX-2 / / PGE2, NF-κB (PGE2 / NF-κB).	human: gastric cancer MGC803	*In vitro*	Inhibition	[146]	mice: macrophages RAW 264.7	*In vitro*	Inhibition	[148]	human: cholangiocarcinoma	*In vitro*	Stimulation	[150]
	human: breast cancer MCF-7, 4T1, MDA-MB-231; mice: breast cancer D2A1, MC7-L1	*In vitro* and *In vivo* mice	Inhibition	[147]	rats: colitis colon samples	*In vivo* rats	Inhibition	[149]	human: esophageal tumor SKGT-4	*In vitro*	Stimulation	[119]
									human: colorectal epithelial cancer cells*[12]→	*In vitro**[12] HT29, Caco2, HCA7, HCT116	Stimulation	[151]
									colorectal cancer	*In vitro/ In vivo (review)*	Stimulation	[152]
**iNOS / NO (P)**:<br>Wnt / β-catenin, NF-κB / iNOS / NF-κB, iNOS / NO / Akt (= PKB);<br>iNOS / mTOR.	murine: peritoneal macrophages	*In vitro*	Inhibition	[153]	mice: macrophages RAW 264.7	*In vitro*	Inhibition	[148]	esophageal adenocarcinoma	*In vitro*	Stimulation	[154]
**TNF-α (P)**:<br>TNF-α / TRAF6 / IκB / PKC / NF-κB / / PANX1 / IL1β;<br>TNF-α / NIK (NF-κB inducing kinase) / / cABL / STAT3 / NF-κB;<br>TNF-α / MAPK / MEK / ERK …;<br>TNF-α / NEMO / IKK / NF-κB.	human: monocytes/ macrophages U-937, THP-1	*In vitro*	Inhibition	[83]	mice: psoriatic skin	*In vivo* mice	Inhibition	[85]	mice: monocyte/ macrophage raw 264.7, Kupffer cells;<br>human: nontumor hepatic LO2	*In vitro* and *In vivo* mice	Stimulation	[110]
**PGE2 (P)**:	mice: Kupffer cells	*In vivo* mice	Inhibition	[155]	mice: macrophages	*In vitro*	Inhibition	[148]	human: colonic fibroblast	*In vitro* and *In vivo*	Stimulation	[157]

Abbreviations: tumor promoter **(P)**, tumor suppressor **(S)**. Symbols used for effects on other targets: stimulation (/), inhibition (\).

**Anticancer therapy targets/ markers, pathways and roles**	**Chloroquine**				**Ivermectin**				**Deoxycholic acid**			
	**Cells**	***In vitro/ In vivo***	**Effect**	**Ref. No.**	**Cells**	***In vitro/ In vivo***	**Effect**	**Ref. No.**	**Cells**	***In vitro/ In vivo***	**Effect**	**Ref. No.**
COX-2 / PGE2 / NF-κB / COX-2.	rats: mesenteric vascular	*In vivo* rats	Inhibition	[156]	RAW 264.7				CCD-18Co and colonic biopsies normal and adenocarcinoma	human biopsies of normal colon and colonic adenocarcinoma		
**8) Angiogenesis and tumor microenvironment associated proteins:**												
**CD31 [g] (= PECAM1), CD34 (P)**		*In vitro/ In vivo (review)*	Inhibition	[158]	human: brain U87, T98G, HBMEC	*In vitro* and *In vivo* mice	Inhibition	[159]	human: colon cancer HCT-116 and colonic biopsies	*In vitro* and *In vivo* mice and human colonic biopsies	Stimulation	[160]
[g] **CD31 (= PECAM1), CD34 (P)**: CD31 / Wnt / β-catenin / TCF / Cyclin D_1 / NF-κB …; CD31 / Akt / JNK / IκBα / NF-κB …; CD31 / Akt / mTOR / HIF / IκBα / NF-κB / HIF / VEGF; CD31 / Akt / iNOS / …; CD31 / Integrin, PI3K, STATs, Notch, HES1; CD34 / MYCIN; CD34 / Notch / JNK / IκBα / NF-κB / …; CD34 / CD31 / Notch / HES1 / NF-κB / …; CD34 / Integrin; CD31 / CD34 / NF-κB / VEGF, ILs.												
**HIFs** (1α, 1β, 2α, 2β) **(P)**: HIFs / IκBα /NF-κB / HIFs; HIF1 / VEGF; TNF-α / NF-κB / HIF-1α; ROS / NF-κB / HIF-1α.	human: colon carcinoma HCT116	*In vitro*	Inhibition	[161]	human: sarcoma U2OS, neuroblastoma Kelly cells	*In vitro*	Inhibition	[162]	human: colonic epithelial Caco-2	*In vitro* and *In vivo* mice	Stimulation	[163]
									mice: osteoblast	*In vitro* and *In vivo* mice	Stimulation	[164]
**VEGF** / VEGFR [h] (∈ RTKs) **(P)**	human: lupus erythematosus patients skin biopsy	*In vivo* human: lupus erythematosus patients skin biopsy	Inhibition	[165]		*In vitro/ In vivo (review)*	Inhibition	[166]	human: colon cancer HCT-116 and colonic biopsies	*In vitro* and *In vivo* mice and human colonic biopsies	Stimulation	[160]
						molecular docking and dynamics simulation	Inhibition	[167]				
[h] **VEGF** / VEGFR (∈ RTKs) **(P)**: VEGF / VEGFR / NF-κB / VEGF. VEGF / VEGFR / PKC / RAS / Raf1 / MEK (MAP2 / ERK kinase) / ERK(1, 2) / Akt / mTOR / IκBα / NF-κB / …; VEGF / VEGFR / Akt / JNK / IκBα / NF-κB; VEGF / VEGFR / Akt / mTOR / HIF / IκBα / NF-κB / HIF / VEGF; VEGF / VEGFR / PKC / iNOS / NO; VEGF / VEGFR / PKC / Wnt / β-catenin, PI3K, Cyclin D_1; VEGF / VEGFR / FAK (PTK2) / PI3K / Akt (or FAK / MEK / ERK / Akt) / … / NF-κB / …; VEGF / VEGFR / PI3K / … / NF-κB / ….												
**MEK / ERK** (= MAPK / ERK = RAS/Raf/MEK/ERK) **(P)** MEK / ERK / NF-κB; EGFR / MEK / ERK / Akt / NF-κB / P-gp; PKC/RAS/Raf/MEK/ERK/TNF/NF-κB; MEK / ERK / Akt / mTOR, JNK, HIF, iNOS, VEGF / IκBα / NF-κB / …; PI3K / PKC / … ; p62 / … / PI3K / … ; p62 / Beclin-1 / PKC / … ; Raf / MEK / ERK \ p38MAPK; p38MAPK / ERK.	human: monocyte-like THP-1, cervical carcinoma HeLa; murine: macrophage RAW264.7, AMJ2C-8	*In vitro* human and murine and *In vivo* human	Inhibition	[123]	human: colorectal HCT-8, breast MCF-7 cancer and chronic myeloid leukemia K562	*In vitro* and *In vivo* mice	Inhibition	[18]	human: gastric cancer SNU601	*In vitro*	Stimulation	[58]
		In vivo rats	Inhibition	[168]								
**p38MAPK** see: **4) Apoptosis and/or necroptosis associated proteins**												
**CXC, CXCL12 (= SDF1), CXCR4 [i] (P)**	human: esophageal squamous cell carcinoma EC109	*In vivo* human surgical resection	Inhibition	[20]	mice: breast cancer	*In vivo* mice	Modulation	[169]	human: monocytes and neutrophils	*In vitro*	Inhibition	[170]

Abbreviations: tumor promoter **(P)**, tumor suppressor **(S)**. Symbols used for effects on other targets: stimulation (/), inhibition (\).

**Anticancer therapy targets/ markers, pathways and roles**	**Chloroquine**				**Ivermectin**				**Deoxycholic acid**			
	**Cells**	***In vitro/ In vivo***	**Effect**	**Ref. No.**	**Cells**	***In vitro/ In vivo***	**Effect**	**Ref. No.**	**Cells**	***In vitro/ In vivo***	**Effect**	**Ref. No.**
[i] **CXC, CXCL12 (= SDF1), CXCR4 (P)**: CXC (∍ CXCL12= SDF1) / receptor CXCR4; CXCL12 / STAT3, NF-κB, HIF-1α, VEGF; CXCL12 / CXCR4 / RAS, JAK, STAT, PI3K, FAK, JNK, Akt, p38MAPK, ERK, NF-κB.												
**FAK**[j] (= PTK2 ∈ NRTK) **(P)**		*In vivo Plasmodium falciparum*	Inhibition	[93]	human: colorectal HCT-8, HCT-116 and breast MCF-7, MDA-MB-231 cancer	*In vitro* and *In vivo* mice	Inhibition	[10]	human: rectal carcinoma HCA-7	*In vitro*	Modulation	[95]
[j] **FAK** (= PTK2 ∈ NRTK) **(P):** FAK / ILs, PKC, p38MAPK, ERK (1, 2), EGFR, Wnt, β-catenin, Integrin β1, FAK, PI3K, Akt, JNK, IκBα, NF-κB, TCF, Cyclin D1; FAK \ P53 (wild-type).												
**9) Cellular metabolism and metabolic reprogramming associated proteins:**												
**GLUTs** (GLUT1-13) **(P)** NF-κB / GLUT; GLUT / PI3K, Akt, IκB, NF-κB; (IκB = IKKi = IKKα + IKKβ + IKKγ ; IKKγ = NEMO) p53 (wild-type) \ GLUT.	rats: skeletal muscle L6	*In vitro*	Stimulation	[171]	glioma U87 cells	*In vitro* and *In vivo* rats	Inhibition	[81]	human: normal squamous epithelium HET-1A, Barrett's metaplastic epithelium (QH)	*In vitro*	Modulation	[172]
									mice: serum and caecum, human: plasma and faeces	*In vivo* mice	Modulation	[173]
**TLRs**[k] (TLR1-24)**(P)**	human: embryonic kidney HEK293, HEK293T	*In vitro*	Inhibition	[174]	inflammatory cells	*In vivo* rats	Inhibition	[3]	human: esophageal Barrett's cells and esophageal adenocarcinoma OE33	*In vitro*	Stimulation	[176]
	mice: dendritic cells	*In vivo* mice	Inhibition	[175]								
[k] **TLRs** (TLR1-24) **(P)**: TLRs / IκB / NF-κB; TLRs / p38MAPK / PI3K / Akt / IκBα / NF-κB; TLRs / p38MAPK / JNK / ERK / Akt / IκBα / NF-κB /…; TLRs / IL1β, IL18 / IκB / NF-κB /…; TLR4 / TNF-α, IL1β / IκB / NF-κB /…; TLR2 / Caspase / PANAX1 / ILs / NF-κB / …												
**10) Embryonic development associated proteins:**												
**TGF-β / SMAD**[l] **(P)**		*In vitro/ In vivo (review)*	Inhibition	[177]	human: hypertrophic scar fibroblasts	*In vivo* patients hypertrophic scar biopsies	Inhibition	[178]		*In vivo* Crohn's disease patients	Modulation	[179]
[l] **TGF-β / SMAD (P)**: TGF-β / MAP3K7 (TAK) / MEK / Akt / NF-κB, SMAD; SMAD / Wnt, β-catenin, Notch, Hippo, Hedgehog, MAPK, PI3K, Akt, NF-κB, JAK, STAT, MUC2; TNF-α \ TGF-β; ROS, Integrins / TGF-β; TGF-β / SMAD / p38MAPK, ERK(1, 2), JNKs, EGFR, IκBα, NF-κB; TGF-β \ C-MYC, CDKs.												
**FAK:** see **8) Angiogenesis and tumor microenvironment associated proteins**												
**11) Cancer stem cells (CSC) associated proteins:**												
**STATs**: see **1) Transcription factors**												
**Nanog / KFL4 / Sox2 / Oct4** see **1) Transcription factors**												
**JAK** see **3) Cell signaling cascades proteins**												

Abbreviations: tumor promoter **(P)**, tumor suppressor **(S)**. Symbols used for effects on other targets: stimulation (/), inhibition (\).

**Anticancer therapy targets/ markers, pathways and roles**	**Chloroquine**				**Ivermectin**				**Deoxycholic acid**			
	**Cells**	***In vitro/ In vivo***	**Effect**	**Ref. No.**	**Cells**	***In vitro/ In vivo***	**Effect**	**Ref. No.**	**Cells**	***In vitro/ In vivo***	**Effect**	**Ref. No.**
**FAK:** see **8) Angiogenesis and tumor microenvironment associated proteins**												
**12) Membrane receptors:**												
**PANX1** [m] (pannexin1) **(P)**		*In vitro/ In vivo (review)*	Modulation	[180]	mice: breast cancer	*In vivo* mice	Modulation	[169]	human: squamous oesophageal epithelial HET-1A, metaplastic and dysplastic Barrett's cells QH and GO	*In vitro*	Stimulation	[78]
									mice: macrophage J774	*In vitro*	Modulation	[181]
[m] **PANX1** (pannexin1) **(P)**: TNF-α / TRAF6 / IκB / PKC / NF-κB / PANX1 / IL1β, NF-κB; P2X7 / TLR2 / Caspase / PANX1 / ROS, ILs (IL1β, IL18) / NF-κB / PANX1.												
**P2X4, P2X7 (P)**: P2X4, P2X7 / NF-κB, ROS; P2X7 / TLR2 / Caspase / PANX1 / ROS, ILs / NF-κB / … .	rats: NR8383 alveolar macrophage ATCC; mice: J774, RAW264; human: alveolar macrophages THP-1	*In vitro*	Stimulation	[182]	rats: NR8383 alveolar macrophage ATCC; mice: J774, RAW264; human: alveolar macrophages THP-1	*In vitro*	Stimulation	[182]	human: squamous oesophageal epithelial HET-1A, metaplastic and dysplastic Barrett's cells QH and GO	*In vitro*	Stimulation	[78]
					mice: breast cancer	*In vivo* mice	Modulation	[169]	mice: macrophage J774	*In vitro*	Modulation	[181]
**CXCR4** [n] **(P)**	human: esophageal squamous cell carcinoma EC109	*In vivo* human surgical resection	Inhibition	[20]	mice: breast cancer	*In vivo* mice	Modulation	[169]	human: monocytes and neutrophils	*In vitro*	Inhibition	[170]
[n] **CXCR4 (P)**: CXCL12 (= SDF1) / CXCR4 / PKC, PI3K, MAPK, RAS, ERK (1, 2), MEK, Akt, JAK, STAT, NF-κB; CXCR4 \ Bcl-2.												
**13) Membrane bound proteins (transcription factors) shuttling between the cytoplasm and nucleus:**												
**Notch (P)**: Notch / HES1, JNK, NF-κB, VEGF, Cyclin D_1, CDKs (4, 6), C-MYC.	human: T-cell acute lymphoblastic leukemia	*In vivo* leukemia patients	Inhibition	[183]	human: ovarian cancer TOV-21G, A2780	*In vitro*	Inhibition	[184]	human: esophageal adenocarcinoma OE19, OE33	*In vitro*	Inhibition	[185]
									human esophageal: epithelial Het-1A cells and surgical specimens normal, metaplastic	*In vitro* and *In vivo*	Inhibition	[30]

Abbreviations: tumor promoter **(P)**, tumor suppressor **(S)**. Symbols used for effects on other targets: stimulation (/), inhibition (\).

**Anticancer therapy targets/ markers, pathways and roles**	**Chloroquine**				**Ivermectin**				**Deoxycholic acid**			
	**Cells**	***In vitro/ In vivo***	**Effect**	**Ref. No.**	**Cells**	***In vitro/ In vivo***	**Effect**	**Ref. No.**	**Cells**	***In vitro/ In vivo***	**Effect**	**Ref. No.**
**EGFR**: see **2) Cell cycle proteins**												
**Wnt / β-catenin**: see **3) Cell signaling cascades proteins**												
**IGF-1 / IGF-1R**: see **3) Cell signaling cascades proteins**												
**14) Oncogen addiction targets:**												
**PTKs** ° (RTKs + NRTKs) **(P)**		*In vivo Plasmodium falciparum*	Inhibition	[93]	human: colorectal HCT-8, HCT-116 and breast MCF-7, MDA-MB-231 cancer	*In vitro* and *In vivo* mice	Inhibition	[10]	human: rectal carcinoma HCA-7	*In vitro*	Modulation	[95]
° **PTK** (RTKs + NRTKs) **(P)**.<br>RTKs: IGFR, EGFR, PDGFR, FGFR, VEGFR, PTKs, ROS1s. RTKs / PI3K, Akt, ERK, STAT, ILs.<br>NRTKs: PTK2 (FAK), ABL, JAK. Wnt / β-catenin / Integrin β1 / PTK2 (=FAK ∊ NRTKs) / ILs, PKC, MAPK, EGFR, Wnt, β-catenin, STAT, caspase.												
**MYC** [p] (C-, N-, L-MYC) **(P)**	mice: MYC induced limfoma	*In vivo* mice	Inhibition	[186]	human: pancreatic cancer MIA PaCa-2, PANC-1	*In vitro* and *In vivo* mice	Inhibition	[224]]	human: Barrett's metaplasia and oesophageal adenocarcinoma OE21, OE33, TE-7, colorectal cancer SW-480	*In vivo* patient samples and *In vitro*	Stimulation	[187]
[p] **MYC** (C-, N-, L-MYC) **(P)**: MYC / NF-κB / p50 / C-MYC; RAS / Raf / MAPK / MEK / ERK / NF-κB / MYC / NF-κB; MYC / Cyclins, mTOR, Akt, Oct4, Sox2, Klf4, NRF1, HIFs, CDK4, STATs.<br>MYC \ p21, Bcl-2, p53, PTEN, IGFR, β-catenin, APC, TCF; Wnt, β-catenin, EGF, MAPK , ERK, PI3K, RAS, STATs / MYC; TGF-β, SMADs, HIFs \ MYC.												
**cABL** (ABL, NRTK) **(P)**:<br>PDGF, TGF-β1, TNF (TNFR2) / cABL / / STAT3, NF-κB, CDKs, ROS;<br>TNF (TNFR) / NF-κB, NIK / cABL / / STAT3 / NF-κB.		*In vivo Plasmodium falciparum*	Inhibition	[93]	human: colorectal and breast cancer cells*[13]→	*In vitro**[13] HCT-8, HCT-116, MCF-7, MDA-MB-231 and *In vivo* mice	Inhibition	[10]	human: rectal carcinoma HCA-7	*In vitro*	Modulation	[95]
					human: chronic myeloid leukemia K562, CD34 and normal bone marrow CD34	*In vitro* and *In vivo*	Inhibition	[94]	human: squamous oesophageal epithelial HET-1A, metaplastic and dysplastic Barrett’s cells QH and GO	*In vitro*	Stimulation	[78]
**Raf-1 / RAS**: see **2) Cell cycle proteins**												
**MEK / ERK**: see **8) Angiogenesis and tumor microenvironment associated proteins**												
**PI3K** (downstream are Akt, PKC, NF-κB): see **2) Cell cycle proteins**												

Abbreviations: tumor promoter **(P)**, tumor suppressor **(S)**. Symbols used for effects on other targets: stimulation (/), inhibition (\).

**Anticancer therapy targets/ markers, pathways and roles**	**Chloroquine**				**Ivermectin**				**Deoxycholic acid**			
	**Cells**	***In vitro/ In vivo***	**Effect**	**Ref. No.**	**Cells**	***In vitro/ In vivo***	**Effect**	**Ref. No.**	**Cells**	***In vitro/ In vivo***	**Effect**	**Ref. No.**
**Akt**:<br>see **2) Cell cycle proteins**												
**mTOR**:<br>see **2) Cell cycle proteins**												
**15) Tumor suppressor rescue targets / markers:**												
**cABL**:<br>see **14) Oncogen addiction targets**												
**PTEN**:<br>see: **3) Cell signaling cascades proteins**												
**16) Some special cancer related processes (integrally evaluated):**												
**Apoptosis (S)**:<br>PI3K/Akt\p53; NF-κB\caspase 8/caspase 3;<br>TNFα/IL-1β/TLRs/IKK/NF-κB/Bcl-2;<br>IKK\IκB; RTKs/PI3K/Akt /mTOR;<br>PTEN\PI3K/Akt/BAX/caspase 9;<br>ROS /JNK / BAX, p38 MAPK / caspase 3;<br>ERK(1, 2) / Bcl-2/MCL-1 \ caspase 9;<br>YAP/ Bcl-2\caspase3; β-catenin\caspase 3;<br>Wnt/β-catenin/c-MYC/Bcl-2; NOTCH/HES1;<br>NOTCH/Bcl-2\caspase 3;<br>TGF-β / TGFβR / SMAD (2,3) / FAS;<br>TGF-β / apoptosis (early-stage cancer);<br>TGF-β \ apoptosis (late-stage cancer).	human: glioma NCE-G22, G44, G62, G63, G84, G112, G120, G121, G130, G168, G260	*In vitro* and *In vivo* mice	Stimulation	[111]	human: urothelial carcinoma T24, RT4	*In vitro*	Stimulation	[48]	Barrett's epithelial cells BAR-T, BAR-T10	*In vitro* and *In vivo* patients	Inhibition	[6]
	human: cholangiocarcinoma QBC939	*In vitro* human	Stimulation	[127]	human: neuroblastoma SH-SY5Y	*In vitro*	Stimulation	[66]	human: gastric cancer SNU601	*In vitro*	Inhibition	[58]
	human: osteosarcoma MG-63	*In vitro* human	Stimulation	[188]								
**Necroptosis / pyroptosis / ferroptosis**		*In vitro/ In vivo (review)*	Stimulation	[158]		*In vitro/ In vivo (review)*	Stimulation	[189]	human: gastric cancer SNU601	*In vitro*	Inhibition	[58]
**Neoangiogenesis (P)**:<br>HIF-1α/VEGF-A/VEGFR2/PKC/MAPK;<br>VEGF-A/VEGFR2/PI3K/AKT/eNOS;<br>VEGFR1\VEGFR2; HIF-1α/VEGF/PDGF;<br>VEGF/FGF/PDGF/RTKs/ PI3K/AKT/mTOR;<br>mTOR/HIF-1α;AKT/eNOS/NO;<br>PTEN\PI3K/AKT; JNK/VEGF; IκB\NF-κB;<br>VEGF/FGF/RAS/RAF/MEK/ERK(1,2);<br>TNFα/IL-1β/IKK/NF-κB /VEGF/IL-8;<br>TGF-β/TGFβR/SMAD (2,3)/VEGF;<br>SMAD7\TGF-β; Wnt/β-catenin /VEGF;<br>VEGF/NOTCH1/HES1; Integrins/FAK/PI3K.	human: umbilical vein endothelial cells	*In vitro* human<br>*In vivo* chicken: chorioallantoic membrane model and mice: malignant pleural effusion model	Inhibition	[190]	human: brain U87, T98G, HBMEC	*In vitro* and *In vivo* mice	Inhibition	[159]	human: colon cancer HCT-116 and colonic biopsies	*In vitro* and *In vivo* mice and human colonic biopsies	Stimulation	[160]
	human: umbilical vein endothelial cells	*In vitro* human<br>*In vivo* chicken: embryo chorioallantoic membrane model	Inhibition	[191]								
**Autophagy (S)**: see also<br>**6) Autophagy (ATG) related proteins**<br>Autophagy \ NF-κB; autophagy / apoptosis;		*In vitro/ In vivo (review)*	Inhibition	[192]	lung adenocarcinoma	*In vitro*	Stimulation	[132]	human: colon NCM-460, colon cancer HCT-116RC	*In vitro*	Stimulation	[134]

Abbreviations: tumor promoter **(P)**, tumor suppressor **(S)**. Symbols used for effects on other targets: stimulation (/), inhibition (\).

**Anticancer therapy targets/ markers, pathways and roles**	**Chloroquine**				**Ivermectin**				**Deoxycholic acid**			
	**Cells**	***In vitro/ In vivo***	**Effect**	**Ref. No.**	**Cells**	***In vitro/ In vivo***	**Effect**	**Ref. No.**	**Cells**	***In vitro/ In vivo***	**Effect**	**Ref. No.**
NF-κB / apoptosis; NF-κB \ apoptosis;<br>TNF, ROS / (NF-κB/HIF-1α), (NF-κB\p53),<br>mTOR \ autophagy;<br>NF-κB / p65 / Beclin1 / autophagy;<br>TLR \ (NF-κB / autophagy);<br>Beclin1 / Bcl-2 \ autophagy;<br>ATG6 / Beclin1;<br>PI3K, PKB, Akt, IGF, NF-κB / mTOR \ \ autophagy;<br>Bcl-2, Bcl-x1 / (Beclin1 / autophagy);<br>PTEN, p53 / autophagy;<br>Bcl, Akt, PI3K, NF-κB / autophagy;<br>TRAIL, TNF, JNK / autophagy;<br>p53 \ autophagy;<br>Beclin1, ATG3, ATG4D, ATG5 / mTOR \ \ autophagy;<br>NF-κB / Beclin1, TLR / autophagy;<br>IKK / AMPK \ mTOR / JNK, p53, Bcl-2, Beclin1, PAK1 / autophagy.		*In vitro/<br>In vivo<br>(review)*	Inhibition	[193]	human:<br>breast cancer<br>MDA-MB-435,<br>HS578T, 4T1,<br>HEK 293T,<br>MCF-7,<br>MDA-MB-231,<br>MDA-MB-468,<br>MDA-MB-361	*In vitro* and<br>*In vivo* mice<br>and breast<br>cancer<br>patients	Stimulation	[195]	human:<br>epithelial<br>HET-1A,<br>Barrett's<br>esophagus<br>derived CP-A<br>and CP-C,<br>esophageal<br>cancer<br>JH-EsoAd1	*In vitro/<br>In vivo*<br>patients and<br>rats	Stimulation<br>(acute<br>exposure) or<br>Inhibition<br>(chronic<br>exposure)	[138]
	human:<br>breast cancer<br>MCF-7	*In vitro*	Inhibition	[194]	mice:<br>RAW264.7<br>macrophages<br>ATCC, TIB-71	*In vitro*	Stimulation	[196]	mice<br>hepatocytes<br>CD95	*In vitro/*	Stimulation	[51]
					human<br>esophageal: normal<br>Het-1A and<br>squamous cell<br>carcinoma<br>KYSE30, KYSE70	*In vitro* and<br>*In vivo*<br>mice	Stimulation	[197]		*In vitro/<br>In vivo<br>(review)*	Stimulation	[135]
**PAK1 (P):**<br>EGF, VEGF, PI3K, Akt, JAK2, ERK, mTOR / PAK1 / Raf, MEK1, Cyclin D$_1$, Cyclin B$_1$, RAS, MAPK, HIFs, p38, PI3K, Akt, Beclin-1, mTOR, ROS, Wnt/β-catenin, JNK, TLR4, NF-κB, ATG5, ATG12, ATG16L, TGF-β, STAT5,		*In vitro/<br>In vivo<br>(review)*	Inhibition	[198]		*In vitro/<br>In vivo<br>(review)*	Inhibition	[198]				

Experimental animals.

The research performed on hamsters followed internationally recognized guidelines on animal welfare, as well as local and national regulations. Hamster usage in our experiment conformed to the recommendation of the Helsinki Declaration. Injections were given painlessly. The experiments performed for this research were carried out in accordance with the legal provisions on animal protection and welfare, particularly the Directive 2010/63/EU dated 22nd September 2010 on the protection of animals used for scientific purposes and the "Guide for the Care and Use of Laboratory Animals" 8th Ed. (USA National Academy of Science 2011), as well as the Law on animal welfare of the Republic of Serbia dated 10 June, 2009 and the University of Novi Sad Rules for work with experimental animals, dated 11th June, 2020. Experiments on animals were performed in accordance with the U.K. Animals (Scientific Procedures) Act and associated guidelines, the National Institutes of Health Guide for The Care and Use of Laboratory Animals, the ARRIVE guidelines and the AVMA euthanasia guidelines 2020 Ed. The tumor burden did not exceed the recommendations of the "Standard Operating Procedure - Tumor Burdon Guidelines: Neoplasia Proposals in Rodents" University of Pittsburgh Institutional Animal Care and Use Committee guidelines 16th July 2018 (max. dimension 3.5 cm in any direction, 10% of animal body weight). According to the rationale of the research program, the principles of work safety were provided.

Humane endpoints were established: significant body weight loss (20%), decreased activity/responsiveness with loss of body weight, impaired posture, inability to eat, urinate or defecate, tumor diameter >3.5 cm, tumor burden >10% body weight, or tumor ulceration. The following aspects were monitored: behavior, general condition, body weight (measured daily), general clinical signs (diarrhea, neurological signs, breathing disorders), tumor diameter, anatomical location, incidence of multiple tumors and tumor ulceration.

BHK-21/C13 cell culture.

BHK-21/C13 cells were cultured in DMEM media with 4.5 g/L glucose (Capricorn Scientific, GmbH, Ebsdorfergrund, Hesse, Germany), supplemented with 10% fetal bovine serum (FBS; Capricorn Scientific, GmbH, Ebsdorfergrund, Hesse, Germany), 2 mM glutamine (Capricorn Scientific, GmbH, Ebsdorfergrund, Hesse, Germany) and 1% penicillin/streptomycin (Capricorn Scientific, GmbH, Ebsdorfergrund, Hesse, Germany), at 37˚C, in a 5% CO_2 humidified atmosphere. The cells were subcultured twice a week once they reached a confluency of 70-80%.

Histological staining procedures.

Tissue sections were stained at room temperature with hematoxylin (Merck KGaA, Darmstadt, Hesse, Germany) for 5 min and with Eosin G for 30 sec. In immunohistochemical staining, the following primary antibodies were used: p53 (Thermo Fisher Scientific Inc., Waltham, MA, USA; cat. no. 7157-MSM18-P0; clone rBP53-12; host specie: mouse; dilution 1:100; positive control: MDA-MB-231 cells, HeLa cell lysate, breast or colon carcinoma; negative control: omitting the primary antibody), Ki-67 (Thermo Fisher Scientific, Inc., Waltham, MA, USA; cat. no. MA5-14520; clone SP6; host specie: rabbit; dilution 1:200; positive control: human tonsil and testis tissues, rat esophagus, mouse embryonic skin tissue; negative control: omitting the primary antibody), PCNA (Thermo Fisher Scientific, Inc., Waltham, MA, USA; cat. no. PA5-27214; class: polyclonal; host specie: rabbit; dilution 1:100; positive control: 293T, A431, HeLa, HepG2, NIH-3T3, PC-12, A549, SiHa; negative control: omitting the primary antibody), CD34 (Abcam, Cambridge, UK; cat. no. ab81289; clone EP373Y; host specie: rabbit; dilution 1:200; positive control: human tonsil or human small intestine tissue; negative

control: omitting the primary antibody), CD31 (Abcam, Cambridge, UK; cat. no. ab28364; clone: polyclonal; host specie: rabbit; dilution 1:50; positive control: human tonsil tissue; negative control: omitting the primary antibody), COX4 (Abcam, Cambridge, UK; cat. no. ab16056; clone: polyclonal; host specie: rabbit; dilution 1:1,000; positive control: human skeletal muscle, heart and liver tissues, human HeLa cells; negative control: omitting the primary antibody), Cytochrome C (Abcam, Cambridge, UK; cat. no. ab133504; clone: EPR1327; host specie: rabbit; dilution 1:500; positive control: HeLa cells, SH-SY5Y cells, human fetal kidney/heart lysates; negative control: omitting the primary antibody), GLUT1 (Thermo Fisher Scientific, Inc., Waltham, MA, USA; cat. no. RB-9052-P0; clone: polyclonal; host specie: rabbit; dilution 1:200; positive control: rat brain lysate, breast carcinoma, squamous cell carcinoma, non-small-cell lung carcinoma, esophageal carcinoma; negative control: omitting the primary antibody), iNOS (Thermo Fisher Scientific, Inc., Waltham, MA, USA; cat. no. PA1-036; clone: polyclonal; host specie: rabbit; dilution 1:20; positive control: LPS-stimulated RAW 264.7 cells, NIH-3T3 cells, A549 cells; negative control: omitting the primary antibody), and caspase 3 (cleaved Asp175) (Abcam, Cambridge, UK; cat. no. ab179517; clone: EPR16888; host species: rabbit; dilution: 1:2,000; positive control: apoptotic tumor or staurosporine-treated rodent tissue; negative control: omission of primary antibody). Briefly, sections (4 μm) were deparaffinized in xylene (100%) (Merck KGaA, Darmstadt, Hesse, Germany) and rehydrated in descending ethanol series (100% twice for 3 min; 95% for 3 min and 70% for 3 min). For antigen retrieval, the sections were microwaved (850W; ~98˚C) for 20 min in Tris-EDTA buffer [10 mM Tris Base (Merck KGaA, Darmstadt, Hesse, Germany), 1 mM EDTA solution (Merck KGaA, Darmstadt, Hesse, Germany), 0.05% Tween 20 (pH 9.0) (Merck KGaA, Darmstadt, Hesse, Germany)], washed twice for 5 min with TBS (Merck KGaA, Darmstadt, Hesse, Germany) plus 0.025% Triton X-100 (Merck KGaA, Darmstadt, Hesse, Germany) (with agitation) and blocked by immersion in 10% goat serum (cat. no. G6767; Sigma-Aldrich, St. Louis, MO, USA) in TBS with 1% BSA (cat. no. T6789; Sigma-Aldrich; Merck KGaA, Darmstadt, Hesse, Germany) for 2 h at room temperature. Primary antibodies dissolved in TBS with 1% BSA were incubated at 4˚C overnight. The sections were washed twice for 5 min with TBS plus 0.025% Triton X-100 (with agitation) and incubated with 0.3% H_2O_2 in TBS for 15 min at room temperature. Horseradish peroxidase-conjugated goat polyclonal rabbit immunoglobulin G secondary antibody (cat. no. ab6721; Abcam, Cambridge, UK) dissolved in TBS with 1% BSA was added to the sections for 2 h at room temperature. The sections were washed three times for 5 min with TBS. For visualization, the chromogen 3,3-diaminobenzidine tetrahydrochloride (cat. no. K3468; Liquid DAB + SubstratChromogen System; Dako; Agilent Technologies, Inc., Santa Clara, CA, USA) was added and incubated for 10 min at room temperature. The sections were washed with water for 5 min and were stained with Mayer's hematoxylin (cat. no. MHS16, Sigma-Aldrich Inc., St. Louis, MO, USA) for 5 min at room temperature. The stained tumor slices were assessed using Leica DMLB 100T (Leica Microsystems GmbH) microscope at x400 magnification. Images were captured using a Leica MC190 HD camera (Leica Microsystems GmbH). Ki-67 and PCNA proteins are cellular markers for proliferation. The images of Ki-67 and PCNA staining were processed using the UTHSCSA Image Tool for Windows version 3.0. In each sample image, individual Ki-67 or PCNA-positive cells were counted. The mean numbers of Ki-67 and PCNA-positive cells in 10 tumor images from each animal were compared among the groups. p53 is a marker of mutational status. CD34 is a marker of endothelial cells and vascular differentiation. CD31 is a useful specific adjunctive marker for tumor vasculature endothelial differentiation. For some tumors, CD34 is less specific and sensitive than CD31. GLUT1, overexpressed in cancer, is associated with glucose transport across membranes. Increased GLUT1 has been identified in various tumors

and serves as a potential diagnostic and prognostic marker. Tumor progression, increased proliferation and angiogenesis are denoted by increased iNOS immunostaining in the cytoplasm. Overexpression of iNOS has been demonstrated in various neoplastic processes, being inversely correlated to differentiation of tumor tissue. COX4 is a mitochondrial Cytochrome C oxidase marker of apoptosis, overexpressed in cancer cells. Cytochrome C is a useful marker of mitochondrial and cellular damage and apoptosis (combined with caspase 3) in tumors. Cleaved caspase 3 is a marker of apoptosis. Immunoexpression for p53, CD34, CD31, GLUT1, iNOS, COX4, Cytochrome C, and caspase 3 was evaluated based on the measured portion of stained surface area (stained surface/whole surface) in the tumor sections (mean of 10 measurements) using UTHSCSA Image Tools for Windows version 3.00.

Blood analyses.

Full blood cell counts were obtained using an auto hematology analyzer (Abacus Junior Vet, Diatron International GmbH, Wiener Neudorf, Niederösterreich, Austria). The serum, extracted by centrifugation at 2,000 x g for 10 min from the blood samples, was analyzed using an auto chemistry analyzer (Rayto Life and Analytical Sciences Co., Ltd., Shenzhen, Guangdong, China) and by commercial tests for concentration determination of glucose (cat. no. 21503, BioSystems S.A., Barcelona, Catalonia, Spain), serum proteins (cat. no. 11500, BioSystems S.A., Barcelona, Catalonia, Spain) and albumins (cat. no. 11547; BioSystems S.A., Barcelona, Catalonia, Spain).

Results.

Supplemental Table S2. Blood analysis for the first four groups in the experiment.

Hamster No	Leuco-cytes (10^9/l)	Lympho-cytes (%)	Mono-cytes (%)	Granulo-cytes (%)	Erythro-cytes (10^{12}/l)	Hemo-globin (g/l)	Hematocrit (%)	MCV	MCH	MCHC (g/l)	Platelets (10^9/l)	Sedimen-tation	Glucose (mM/l)	Serum proteins (g/l)	Albumins (g/l)
Control group															
1	10.51	73.89	2.7	19.4	4.71	147	40.31	67	22.89	345	341	1/2	6.68	56.03	23.39
2	10.08	78.99	0.5	15.2	5.14	135	36.08	72	20.19	331	273	1/2	8.44	60.69	31.30
3	11.38	71.99	3.4	17.6	4.02	152	39.10	74	26.6	349	452	1/2	5.08	50.06	25.68
4	6.04	79.53	2.2	23.3	4.62	129	38.88	67	25.83	361	285	1/2	6.04	72.16	27.27
5	9.11	80.73	1.3	18.0	6.59	138	36.02	66	22.73	340	415	1/2	6.98	49.57	40.94
6	8.63	76.03	3.5	17.8	6.93	152	43.10	69	20.33	352	383	1/2	7.07	54.34	21.75
Mean	9.29	76.86	2.28	18.57	5.34	142.17	38.92	69.17	23.10	346.33	358.17		6.72	57.14	28.39
±SD	1.87	3.46	1.18	2.70	1.17	9.58	2.68	3.19	2.68	10.31	71.49		1.12	8.42	6.98
Group treated with chloroquine															
1	10.12	80.92	2.6	20.6	4.38	147	36.80	69	21.47	372	430	1/2	8.86	49.84	36.77
2	8.77	82.68	5.3	12.6	5.32	108	40.64	65	22.40	346	412	1/2	6.01	65.42	24.17
3	13.81	74.71	0.9	16.0	5.68	131	40.74	71	23.01	355	375	1/2	6.09	57.28	32.12
4	6.57	71.03	1.8	22.4	5.05	137	39.63	74	23.63	371	297	1/2	8.63	50.79	22.35
5	7.35	78.43	2.0	19.5	4.83	127	41.35	68	24.03	339	294	1/2	5.15	46.30	24.96
6	10.34	81.65	3.7	18.8	6.91	139	38.75	69	23.85	331	337	1/2	5.96	67.55	27.85
Mean	9.49	78.24	2.73	18.32	5.36	131.50	39.65	69.33	23.07	352.33	357.50		6.78	56.20	28.04
±SD	2.58	4.54	1.56	3.51	0.88	13.41	1.67	3.01	0.99	16.82	57.70		1.56	8.75	5.47

Group treated with ivermectin															
1	9.84	78.63	2.8	18.8	6.77	141	38.08	68	23.27	342	338	1/2	5.14	58.78	28.28
2	8.35	77.2	2.8	19.8	6.02	145	40.07	69	24.80	323	268	1/2	6.52	63.59	25.25
3	5.03	82.89	1.4	18.2	5.73	142	41.21	66	22.81	347	353	1/2	7.99	47.71	31.59
4	13.00	78.23	2.6	16.1	3.36	135	37.77	76	23.13	334	395	1/2	5.93	72.28	26.54
5	10.03	71.93	3.2	20.3	6.28	129	40.12	72	27.33	371	347	1/2	6.53	54.75	32.15
6	9.10	81.95	1.8	19.6	6.39	143	42.42	70	24.95	343	417	1/2	7.30	53.96	24.66
Mean	9.23	78.47	2.41	18.78	5.76	139.17	39.95	70.17	24.38	343.33	353.00		6.57	58.51	28.08
±SD	2.59	3.90	0.69	1.52	1.23	6.01	1.79	3.49	1.70	16.01	51.66		1.00	8.57	3.19
Group treated with the combination of chloroquine and ivermectin															
1	14.82	78.97	0.8	17.3	6.89	138	42.35	67	23.03	354	423	1/2	6.30	61.80	39.75
2	11.47	81.21	2.0	20.8	6.82	141	38.21	68	22.69	335	338	1/2	7.52	48.97	22.83
3	5.76	78.29	0.8	17.3	5.81	145	37.51	66	23.11	419	329	1/2	6.06	58.33	28.55
4	8.03	74.63	2.9	22.0	5.6	148	37.41	73	23.03	353	298	1/2	7.40	73.80	24.00
5	9.15	82.97	2.2	17.3	6.69	149	41.89	71	21.21	361	397	1/2	8.51	47.56	26.82
6	11.46	74.15	1.9	17.1	5.97	139	38.17	74	25.75	349	349	1/2	8.25	52.12	27.32
Mean	10.12	78.37	1.75	18.62	6.30	143.33	39.26	69.83	23.14	361.83	355.67		7.34	57.10	28.21
±SD	3.16	3.50	0.81	2.20	0.57	4.68	2.25	3.31	1.47	29.30	46.12		1.00	9.84	6.04

Normal ranges: leucocytes 3-16·10^9/l; lymphocytes 50-81%; monocytes 0-3%; granulocytes 17-30%; erythrocytes 4-7·10^{12}/l; hemoglobin 100-180 g/l; hematocrit 35-50%; MCV 67-77; MCH 23-27; MCHC 200-360 g/l; platelets 200-500·10^9/l; sedimentation 1/2; glucose 3-11 mM/l; serum proteins 45-75 g/l; albumins 23-43 g/l.

References

1. Lashgari, N.A.; Roudsari, N.M.; Ahmadvand, D.; Amirlou, D.; Darban Khales, S.; Ahmadian, D.; Momtaz, S.; Abdolghaffari, A.H.; Jamialahmadi, T.; Sahebkar, A. Chapter 9—Repurposing antiparasitic drugs for the treatment of other diseases. In *Advances in Antiparasitic Therapies and Drug Delivery*; Kesharwani, P., Gupta, N., Eds.; Academic Press: Cambridge, MA, USA, 2024; pp. 161–261. https://doi.org/10.1016/B978-0-443-15178-1.00005-5.
2. Fauzi, Y.R.; Nakahata, S.; Chilmi, S.; Ichikawa, T.; Nueangphuet, P.; Yamaguchi, R.; Nakamura, T.; Shimoda, K.; Morishita, K. Antitumor effects of chloroquine/hydroxychloroquine mediated by inhibition of the NF-κB signaling pathway through abrogation of autophagic p47 degradation in adult T-cell leukemia/lymphoma cells. *PLoS ONE* **2021**, *16*, e0256320. https://doi.org/10.1371/journal.pone.0256320.
3. Khan, M.U.A.; Akhtar, T.; Naseem, N.; Aftab, U.; Hussain, S.; Shahzad, M. Evaluation of therapeutic potential of ivermectin against complete Freund's adjuvant-induced arthritis in rats: Involvement of inflammatory mediators. *Fundam. Clin. Pharmacol.* **2023**, *37*, 971–982. https://doi.org/10.1111/fcp.12902.
4. Gao, X.; Xuan, Y.; Zhou, Z.; Chen, C.; Wen Wang, D.; Wen, Z. Ivermectin ameliorates acute myocarditis via the inhibition of importin-mediated nuclear translocation of NF-κB/p65. *Int. Immunopharmacol*. **2024**, *133*, 112073. https://doi.org/10.1016/j.intimp.2024.112073.
5. Bernstein, H.; Bernstein, C.; Holubec, H.; Dvorakova, B.; Garewal, H. Deoxycholate induces mitochondrial oxidative stress and activates NF-kappaB through multiple mechanisms in HCT-116 colon epithelial cells. *Carcinogenesis* **2007**, *28*, 215–222. https://doi.org/10.1093/carcin/bgl139.
6. Huo, X.; Juergens, S.; Zhang, X.; Rezaei, D.; Yu, C.; Strauch, E.D.; Wang, J.Y.; Cheng, E.; Meyer, F.; Wang, D.H.; et al. Deoxycholic acid causes DNA damage while inducing apoptotic resistance through NF-κB activation in benign Barrett's epithelial cells. *Am. J. Physiol. Gastrointest. Liver Physiol.* **2011**, *301*, G278–G286. https://doi.org/10.1152/ajpgi.00092.2011.
7. Shivakumar, S.; Panigrahi, T.; Shetty, R.; Subramani, M.; Ghosh, A.; Jeyabalan, N. Chloroquine Protects Human Corneal Epithelial Cells from Desiccation Stress Induced Inflammation without Altering the Autophagy Flux. *Biomed. Res. Int.* **2018**, *2018*, 7627329. https://doi.org/10.1155/2018/7627329.

8. Mai, A.; Heba, G.; Enas, G.; Kawkab, A.; Maha, A. Suppression of NLRP3 inflammasome by ivermectin ameliorates bleomycin-induced pulmonary fibrosis. *J. Zhejiang Univ.-Sci. B* **2023**, *24*, 723–733. https://doi.org/10.1631/jzus.B2200385.
9. Gil, D.; Laidler, P.; Zarzycka, M.; Dulińska-Litewka, J. Inhibition Effect of Chloroquine and Integrin-Linked Kinase Knockdown on Translation in Melanoma Cells. *Int. J. Mol. Sci.* **2021**, *22*, 3682. https://doi.org/10.3390/ijms22073682.
10. Jiang, L.; Sun, Y.J.; Song, X.H.; Sun, Y.Y.; Yang, W.Y.; Li, J.; Wu, Y.J. Ivermectin inhibits tumor metastasis by regulating the Wnt/β-catenin/integrin β1/FAK signaling pathway. *Am. J. Cancer Res.* **2022**, *12*, 4502–4519. PMID: 36381328; PMCID: PMC9641399.
11. Jenkins, G.J.; Harries, K.; Doak, S.H.; Wilmes, A.; Griffiths, A.P.; Baxter, J.N.; Parry, J.M. The bile acid deoxycholic acid (DCA) at neutral pH activates NF-kappaB and induces IL-8 expression in oesophageal cells in vitro. *Carcinogenesis* **2004**, *25*, 317–323. https://doi.org/10.1093/carcin/bgh032.
12. Chen, C.; Zhang, H.; Yu, Y.; Huang, Q.; Wang, W.; Niu, J.; Lou, J.; Ren, T.; Huang, Y.; Guo, W. Chloroquine suppresses proliferation and invasion and induces apoptosis of osteosarcoma cells associated with inhibition of phosphorylation of STAT3. *Aging* **2021**, *13*, 17901–17913. https://doi.org/10.18632/aging.203196.
13. Zaidi, A.K.; Dehgani-Mobaraki, P. The mechanisms of action of ivermectin against SARS-CoV-2—An extensive review. *J. Antibiot.* **2022**, *75*, 60–71. https://doi.org/10.1038/s41429-021-00491-6.
14. Kaur, B.; Blavo, C.; Parmar, M.S. Ivermectin: A Multifaceted Drug With a Potential Beyond Anti-parasitic Therapy. *Cureus* **2024**, *16*, e56025. https://doi.org/10.7759/cureus.56025.
15. Lotfalizadeh, N.; Gharib, A.; Hajjafari, A.; Borji, H.; Bayat, Z. The Anticancer Potential of Ivermectin: Mechanisms of Action and Therapeutic Implications. *J. Lab. Anim. Res.* **2022**, *1*, 52–59. https://doi.org/10.58803/jlar.v1i1.11.
16. Jin, D.; Huang, K.; Xu, M.; Hua, H.; Ye, F.; Yan, J.; Zhang, G.; Wang, Y. Deoxycholic acid induces gastric intestinal metaplasia by activating STAT3 signaling and disturbing gastric bile acids metabolism and microbiota. *Gut Microbes.* **2022**, *14*, 2120744. https://doi.org/10.1080/19490976.2022.2120744.
17. Duleh, S.; Wang, X.; Komirenko, A.; Margeta, M. Activation of the Keap1/Nrf2 stress response pathway in autophagic vacuolar myopathies. *Acta Neuropathol. Commun.* **2016**, *4*, 115. https://doi.org/10.1186/s40478-016-0384-6.
18. Jiang, L.; Wang, P.; Sun, Y.J.; Wu, Y.J. Ivermectin reverses the drug resistance in cancer cells through EGFR/ERK/Akt/NF-κB pathway. *J. Exp. Clin. Cancer Res.* **2019**, *38*, 265. https://doi.org/10.1186/s13046-019-1251-7.
19. Liu, J.; Zhang, Y.; Song, M.; Guo, X.; Fan, J.; Tao, S. Taurodeoxycholic acid alleviates diquat-induced intestinal barrier function injury in mice through the upregulation of Nrf2-mediated signaling pathway. *Anim. Dis.* **2024**, *4*, 34. https://doi.org/10.1186/s44149-024-00139-6.
20. Yue, D.; Zhang, D.; Shi, X.; Liu, S.; Li, A.; Wang, D.; Qin, G.; Ping, Y.; Qiao, Y.; Chen, X.; et al. Chloroquine Inhibits Stemness of Esophageal Squamous Cell Carcinoma Cells Through Targeting CXCR4-STAT3 Pathway. *Front. Oncol.* **2020**, *10*, 311. https://doi.org/10.3389/fonc.2020.00311.
21. Dominguez-Gomez, G.; Chavez-Blanco, A.; Medina-Franco, J.L.; Saldivar-Gonzalez, F.; Flores-Torrontegui, Y.; Juarez, M.; Díaz-Chávez, J.; Gonzalez-Fierro, A.; Dueñas-González, A. Ivermectin as an inhibitor of cancer stem-like cells. *Mol. Med. Rep.* **2018**, *17*, 3397–3403. https://doi.org/10.3892/mmr.2017.8231.
22. Chen, M.; Ye, A.; Wei, J.; Wang, R.; Poon, K. Deoxycholic Acid Upregulates the Reprogramming Factors KFL4 and OCT4 Through the IL-6/STAT3 Pathway in Esophageal Adenocarcinoma Cells. *Technol. Cancer Res. Treat.* **2020**, *19*, 1533033820945302. https://doi.org/10.1177/1533033820945302.
23. Sun, L.; Beggs, K.; Borude, P.; Edwards, G.; Bhushan, B.; Walesky, C.; Roy, N.; Manley, M.W., Jr.; Gunewardena, S.; O'Neil, M.; et al. Bile acids promote diethylnitrosamine-induced hepatocellular carcinoma via increased inflammatory signaling. *Am. J. Physiol. Gastrointest. Liver Physiol.* **2016**, *311*, G91–G104. https://doi.org/10.1152/ajpgi.00027.2015.
24. Razani, B.; Feng, C.; Semenkovich, C.F. p53 is required for chloroquine-induced atheroprotection but not insulin sensitization. *J. Lipid Res.* **2010**, *51*, 1738–1746. https://doi.org/10.1194/jlr.M003681.
25. Ma, Y.; Xu, X.; Wu, H.; Li, C.; Zhong, P.; Liu, Z.; Ma, C.; Liu, W.; Wang, C.; Zhang, Y.; et al. Ivermectin contributes to attenuating the severity of acute lung injury in mice. *Biomed. Pharmacother.* **2022**, *155*, 113706. https://doi.org/10.1016/j.biopha.2022.113706.
26. Gupta, S.; Natarajan, R.; Payne, S.G.; Studer, E.J.; Spiegel, S.; Dent, P.; Hylemon, P.B. Deoxycholic acid activates the c-Jun N-terminal kinase pathway via FAS receptor activation in primary hepatocytes. Role of acidic sphingomyelinase-mediated ceramide generation in FAS receptor activation. *J. Biol. Chem.* **2004**, *279*, 5821–5828. https://doi.org/10.1074/jbc.M310979200.
27. Lin, Y.; Zhu, X.; Li, Y.; Dou, Y.; Wang, J.; Qi, R.; Ma, L. LY294002 ameliorates psoriatic skin inflammation in mice via blocking the Notch1/Hes1-PTEN/AKT/IL-17A feedback loop. *Clin. Exp. Immunol.* **2023**, *213*, 114–124. https://doi.org/10.1093/cei/uxad025.

28. Rani, A.; Greenlaw, R.; Smith, R.A.; Galustian, C. HES1 in immunity and cancer. *Cytokine Growth Factor Rev.* **2016**, *30*, 113–117. https://doi.org/10.1016/j.cytogfr.2016.03.010.
29. Wang, W.G.; Jiang, X.F.; Zhang, C.; Zhan, X.P.; Cheng, J.G.; Tao, L.M.; Xu, W.P.; Li, Z.; Zhang, Y. Avermectin induced vascular damage in zebrafish larvae: Association with mitochondria-mediated apoptosis and VEGF/Notch signaling pathway. *J. Hazard. Mater.* **2024**, *477*, 135376. https://doi.org/10.1016/j.jhazmat.2024.135376.
30. Wang, Y.C.; Wang, Z.Q.; Yuan, Y.; Ren, T.; Ni, P.Z.; Chen, L.Q. Notch Signaling Pathway Is Inhibited in the Development of Barrett's Esophagus: An In Vivo and In Vitro Study. *Can. J. Gastroenterol. Hepatol.* **2018**, *2018*, 4149317. https://doi.org/10.1155/2018/4149317.
31. Espina, V.A.; Yeon, S.; VanHouten, J.N.; Wysolmerski, J.; Liotta, L.A. Chloroquine and vitamin D3 modulate proliferation in early stage breast cancer models. [abstract]. In *Proceedings of the 107th Annual Meeting of the American Association for Cancer Research, New Orleans, LA, USA, 16–20 April 2016*; AACR: Philadelphia, PA, USA, 2016; *Cancer Res.* **2016**, *76 (14_Supplement)*, 2859. https://doi.org/10.1158/1538-7445.AM2016-2859.
32. Mathachan, S.R.; Sardana, K.; Khurana, A. Current Use of Ivermectin in Dermatology, Tropical Medicine, and COVID-19: An Update on Pharmacology, Uses, Proven and Varied Proposed Mechanistic Action. *Indian Dermatol. Online J.* **2021**, *12*, 500–514. https://doi.org/10.4103/idoj.idoj_298_21.
33. Thompson, B.; Lu, S.; Revilla, J.; Uddin, M.J.; Oakland, D.N.; Brovero, S.; Keles, S.; Bresnick, E.H.; Petri, W.A.; Burgess, S.L. Secondary bile acids function through the vitamin D receptor in myeloid progenitors to promote myelopoiesis. *Blood Adv.* **2023**, *7*, 4970–4982. https://doi.org/10.1182/bloodadvances.2022009618.
34. Yuan, P.; Hu, Q.; He, X.; Long, Y.; Song, X.; Wu, F.; He, Y.; Zhou, X. Laminar flow inhibits the Hippo/YAP pathway via autophagy and SIRT1-mediated deacetylation against atherosclerosis. *Cell Death Dis.* **2020**, *11*, 141. https://doi.org/10.1038/s41419-020-2343-1.
35. Li, B.; Du, Y.; He, J.; Lv, X.; Liu, S.; Zhang, X.; Zhang, Y. Chloroquine inhibited Helicobacter pylori-related gastric carcinogenesis by YAP-β-catenin-autophagy axis. *Microb. Pathog.* **2023**, *184*, 106388. https://doi.org/10.1016/j.micpath.2023.106388.
36. Nishio, M.; Sugimachi, K.; Goto, H.; Wang, J.; Morikawa, T.; Miyachi, Y.; Takano, Y.; Hikasa, H.; Itoh, T.; Suzuki, S.O.; et al. Dysregulated YAP1/TAZ and TGF-β signaling mediate hepatocarcinogenesis in Mob1a/1b-deficient mice. *PNAS Med. Sci.* **2015**, *113*, E71–E80. https://doi.org/10.1073/pnas.1517188113.
37. Anakk, S.; Bhosale, M.; Schmidt, V.A.; Johnson, R.L.; Finegold, M.J.; Moore, D.D. Bile acids activate YAP to promote liver carcinogenesis. *Cell Rep.* **2013**, *5*, 1060–1069. https://doi.org/10.1016/j.celrep.2013.10.030.
38. Datta, S.; Choudhury, D.; Das, A.; Mukherjee, D.D.; Dasgupta, M.; Bandopadhyay, S.; Chakrabarti, G. Autophagy inhibition with chloroquine reverts paclitaxel resistance and attenuates metastatic potential in human nonsmall lung adenocarcinoma A549 cells via ROS mediated modulation of β-catenin pathway. *Apoptosis* **2019**, *24*, 434. https://doi.org/10.1007/s10495-019-01534-y.
39. Song, P.; Gao, Z.; Bao, Y.; Chen, L.; Huang, Y.; Liu, Y.; Dong, Q.; Wei, X. Wnt/β-catenin signaling pathway in carcinogenesis and cancer therapy. *J. Hematol. Oncol.* **2024**, *17*, 46. https://doi.org/10.1186/s13045-024-01563-4.
40. Melotti, A.; Mas, C.; Kuciak, M.; Lorente-Trigos, A.; Borges, I.; Ruiz i Altaba, A. The river blindness drug Ivermectin and related macrocyclic lactones inhibit WNT-TCF pathway responses in human cancer. *EMBO Mol. Med.* **2014**, *6*, 1263–1278. https://doi.org/10.15252/emmm.201404084.
41. Pai, R.; Tarnawski, A.S.; Tran, T. Deoxycholic acid activates β-catenin signaling pathway and increases colon cell cancer growth and invasiveness. *Mol. Biol. Cell* **2004**, *15*, 2156–2163. https://doi.org/10.1091/mbc.e03-12-0894.
42. Dettmar, P.W.; Strugala, V.; Tselepis, C. The effect of alginates on deoxycholic-acid-induced changes in oesophageal mucosal biology at pH 4. *J. Biomater. Sci. Polym. Ed.* **2007**, *18*, 317–333. https://doi.org/10.1163/156856207779996922.
43. Zhang, S.; Zhu, C.; Liu, Q.; Wang, W. Effects of chloroquine on GFAP, PCNA and cyclin D1 in hippocampus and cerebral cortex of rats with seizures induced by pentylenetetrazole. *J. Huazhong Univ. Sci. Technol. Med. Sci.* **2005**, *25*, 625–628. https://doi.org/10.1007/BF02896153.
44. Choi, J.-H.; Yoon, J.S.; Won, Y.W.; Park, B.B.; Lee, Y.Y. Chloroquine enhances the chemotherapeutic activity of 5-fluorouracil in a colon cancer cell line via cell cycle alteration. *APMIS* **2012**, *120*, 597–604. https://doi.org/10.1111/j.1600-0463.2012.02876.x.
45. Jang, J.Y.; Im, E.; Choi, Y.H.; Kim, N.D. Mechanism of Bile Acid-Induced Programmed Cell Death and Drug Discovery against Cancer: A Review. *Int. J. Mol. Sci.* **2022**, *23*, 7184. https://doi.org/10.3390/ijms23137184.
46. Hwang, J.R.; Kim, W.Y.; Cho, Y.J.; Ryu, J.Y.; Choi, J.J.; Jeong, S.Y.; Kim, M.S.; Kim, J.H.; Paik, E.S.; Lee, Y.Y.; et al. Chloroquine reverses chemoresistance via upregulation of p21WAF1/CIP1 and autophagy inhibition in ovarian cancer. *Cell Death Dis.* **2020**, *11*, 1034. https://doi.org/10.1038/s41419-020-03242-x.

47. Sasaki, K.; Tsuno, N.H.; Sunami, E.; Tsurita, G.; Kawai, K.; Okaji, Y.; Nishikawa, T.; Shuno, Y.; Hongo, K.; Hiyoshi, M.; et al. Chloroquine potentiates the anti-cancer effect of 5-fluorouracil on colon cancer cells. *BMC Cancer* **2010**, *10*, 370. https://doi.org/10.1186/1471-2407-10-370.
48. Tung, C.L.; Chao, W.Y.; Li, Y.Z.; Shen, C.H.; Zhao, P.W.; Chen, S.H.; Wu, T.Y.; Lee, Y.R. Ivermectin induces cell cycle arrest and caspase-dependent apoptosis in human urothelial carcinoma cells. *Int. J. Med. Sci.* **2022**, *19*, 1567–1575. https://doi.org/10.7150/ijms.76623.
49. Kodama, M.; Kodama, T.; Newberg, J.Y.; Katayama, H.; Kobayashi, M.; Hanash, S.M.; Yoshihara, K.; Wei, Z.; Tien, J.C.; Rangel, R.; et al. In vivo loss-of-function screens identify KPNB1 as a new druggable oncogene in epithelial ovarian cancer. *PNAS Biol. Sci.* **2017**, *114*, E7301–E7310. https://doi.org/10.1073/pnas.1705441114.
50. Qiao, L.; McKinstry, R.; Gupta, S.; Gilfor, D.; Windle, J.J.; Hylemon, P.B.; Grant, S.; Fisher, P.B.; Dent, P. Cyclin kinase inhibitor p21 potentiates bile acid-induced apoptosis in hepatocytes that is dependent on p53. *Hepatology* **2002**, *36*, 39–48. https://doi.org/10.1053/jhep.2002.33899.
51. Zhang, G.; Park, M.A.; Mitchell, C.; Walker, T.; Hamed, H.; Studer, E.; Graf, M.; Rahmani, M.; Gupta, S.; Hylemon, P.B.; et al. Multiple cyclin kinase inhibitors promote bile acid-induced apoptosis and autophagy in primary hepatocytes via p53-CD95-dependent signaling. *J. Biol. Chem.* **2008**, *283*, 24343–24358. https://doi.org/10.1074/jbc.M803444200.
52. Müller, A.; Weyerhäuser, P.; Berte, N.; Jonin, F.; Lyubarskyy, B.; Sprang, B.; Kantelhardt, S.R.; Salinas, G.; Opitz, L.; Schulz-Schaeffer, W.; et al. Concurrent Activation of Both Survival-Promoting and Death-Inducing Signaling by Chloroquine in Glioblastoma Stem Cells: Implications for Potential Risks and Benefits of Using Chloroquine as Radiosensitizer. *Cells* **2023**, *12*, 1290. https://doi.org/10.3390/cells12091290.
53. Agalakova, N.I. Chloroquine and Chemotherapeutic Compounds in Experimental Cancer Treatment. *Int. J. Mol. Sci.* **2024**, *25*, 945. https://doi.org/10.3390/ijms25020945.
54. Xu, N.; Lu, M.; Wang, J.; Li, Y.; Yang, X.; Wei, X.; Si, J.; Han, J.; Yao, X.; Zhang, J.; et al. Ivermectin induces apoptosis of esophageal squamous cell carcinoma via mitochondrial pathway. *BMC Cancer* **2021**, *21*, 1307. https://doi.org/10.1186/s12885-021-09021-x.
55. Dong, W.; Liu, L.; Dou, Y.; Xu, M.; Liu, T.; Wang, S.; Zhang, Y.; Deng, B.; Wang, B.; Cao, H. Deoxycholic acid activates epidermal growth factor receptor and promotes intestinal carcinogenesis by ADAM17-dependent ligand release. *J. Cell. Mol. Med.* **2018**, *22*, 4263–4273. https://doi.org/10.1111/jcmm.13709.
56. Ochsenkühn, T.; Bayerdörffer, E.; Meining, A.; Schinkel, M.; Thiede, C.; Nüssler, V.; Sackmann, M.; Hatz, R.; Neubauer, A.; Paumgartner, G. Colonic mucosal proliferation is related to serum deoxycholic acid levels. *Cancer* **1999**, *85*, 1664–1669. https://doi.org/10.1002/(SICI)1097-0142(19990415)85:8<1664::AID-CNCR4>3.0.CO;2-O.
57. Liu, L.; Han, C.; Yu, H.; Zhu, W.; Cui, H.; Zheng, L.; Zhang, C.; Yue, L. Chloroquine inhibits cell growth in human A549 lung cancer cells by blocking autophagy and inducing mitochondrial-mediated apoptosis. *Oncol. Rep.* **2018**, *39*, 2807–2816. https://doi.org/10.3892/or.2018.6363.
58. Lim, S.C.; Duong, H.Q.; Parajuli, K.R.; Han, S.I. Pro-apoptotic role of the MEK/ERK pathway in ursodeoxycholic acid-induced apoptosis in SNU601 gastric cancer cells. *Oncol. Rep.* **2012**, *28*, 1429–1434. https://doi.org/10.3892/or.2012.1918.
59. Fedele, A.O.; Proud, C.G. Chloroquine and bafilomycin A mimic lysosomal storage disorders and impair mTORC1 signalling. *Biosci. Rep.* **2020**, *40*, BSR20200905. https://doi.org/10.1042/BSR20200905.
60. Tang, M.; Hu, X.; Wang, Y.; Yao, X.; Zhang, W.; Yu, C.; Cheng, F.; Li, J.; Fang, Q. Ivermectin, a potential anticancer drug derived from an antiparasitic drug. *Pharmacol. Res.* **2021**, *163*, 105207. https://doi.org/10.1016/j.phrs.2020.105207.
61. Marin, J.J.; Hernandez, A.; Revuelta, I.E.; Gonzalez-Sanchez, E.; Gonzalez-Buitrago, J.M.; Perez, M.J. Mitochondrial genome depletion in human liver cells abolishes bile acid-induced apoptosis: Role of the Akt/mTOR survival pathway and Bcl-2 family proteins. *Free Radic. Biol. Med.* **2013**, *61*, 218–228. https://doi.org/10.1016/j.freeradbiomed.2013.04.002.
62. Kim, J.H.; Choi, H.S.; Lee, D.S. Primaquine Inhibits the Endosomal Trafficking and Nuclear Localization of EGFR and Induces the Apoptosis of Breast Cancer Cells by Nuclear EGFR/Stat3-Mediated c-Myc Downregulation. *Int. J. Mol. Sci.* **2021**, *22*, 12961. https://doi.org/10.3390/ijms222312961.
63. Caliceti, C.; Punzo, A.; Silla, A.; Simoni, P.; Roda, G.; Hrelia, S. New Insights into Bile Acids Related Signaling Pathways in the Onset of Colorectal Cancer. *Nutrients* **2022**, *14*, 2964. https://doi.org/10.3390/nu14142964.
64. Morgan, M.J.; Gamez, G.; Menke, C.; Hernandez, A.; Thorburn, J.; Gidan, F.; Staskiewicz, L.; Morgan, S.; Cummings, C.; Maycotte, P.; et al. Regulation of autophagy and chloroquine sensitivity by oncogenic RAS in vitro is context-dependent. *Autophagy* **2014**, *10*, 1814–1826. https://doi.org/10.4161/auto.32135.

65. McCubrey, J.A.; Abrams, S.L.; Follo, M.Y.; Manzoli, L.; Ratti, S.; Martelli, A.M.; Cervello, M. Effects of chloroquine and hydroxychloroquine on the sensitivity of pancreatic cancer cells to targeted therapies. *Adv. Biol. Regul.* **2023**, *87*, 100917. https://doi.org/10.1016/j.jbior.2022.100917.
66. Zhang, Y.; Sun, T.; Li, M.; Lin, Y.; Liu, Y.; Tang, S.; Dai, C. Ivermectin-Induced Apoptotic Cell Death in Human SH-SY5Y Cells Involves the Activation of Oxidative Stress and Mitochondrial Pathway and Akt/mTOR-Pathway-Mediated Autophagy. *Antioxidants* **2022**, *11*, 908. https://doi.org/10.3390/antiox11050908.
67. Rust, C.; Karnitz, L.M.; Paya, C.V.; Moscat, J.; Simari, R.D.; Gores, G.J. The bile acid taurochenodeoxycholate activates a phosphatidylinositol 3-kinase-dependent survival signaling cascade. *J. Biol. Chem.* **2000**, *275*, 20210–20216. https://doi.org/10.1074/jbc.M909992199.
68. Mühlbauer, M.; Allard, B.; Bosserhoff, A.K.; Kiessling, S.; Herfarth, H.; Rogler, G.; Schölmerich, J.; Jobin, C.; Hellerbrand, C. Differential effects of deoxycholic acid and taurodeoxycholic acid on NF-kappa B signal transduction and IL-8 gene expression in colonic epithelial cells. *Am. J. Physiol. Gastrointest. Liver Physiol.* **2004**, *286*, G1000–G1008. https://doi.org/10.1152/ajpgi.00338.2003.
69. Dey, S.; Reddy, K.V.V.S.N.; Wadhwani, A. Doxorubicin and chloroquine: A combination therapy to overcome the multi drug resistance in cancer—A review. *Int. J. Pharm. Sci. Res.* **2019**, *4*, 5266–5273. https://doi.org/10.13040/IJPSR.0975-8232.10(12).5266-73.
70. Inokuchi, A.; Hinoshita, E.; Iwamoto, Y.; Kohno, K.; Kuwano, M.; Uchiumi, T. Enhanced expression of the human multidrug resistance protein 3 by bile salt in human enterocytes. A transcriptional control of a plausible bile acid transporter. *J. Biol. Chem.* **2001**, *276*, 46822–46829. https://doi.org/10.1074/jbc.M104612200.
71. Kong, Y.; Bai, P.S.; Sun, H.; Nan, K.J.; Chen, N.Z.; Qi, X.G. The deoxycholic acid targets miRNA-dependent CAC1 gene expression in multidrug resistance of human colorectal cancer. *Int. J. Biochem. Cell Biol.* **2012**, *44*, 2321–2332. https://doi.org/10.1016/j.biocel.2012.08.006.
72. Balic, A.; Sørensen, M.D.; Trabulo, S.M.; Sainz, B., Jr.; Cioffi, M.; Vieira, C.R.; Miranda-Lorenzo, I.; Hidalgo, M.; Kleeff, J.; Erkan, M.; et al. Chloroquine targets pancreatic cancer stem cells via inhibition of CXCR4 and hedgehog signaling. *Mol. Cancer Ther.* **2014**, *13*, 1758–1771. https://doi.org/10.1158/1535-7163.MCT-13-0948.
73. James, C.E.; Davey, M.W. Increased expression of ABC transport proteins is associated with ivermectin resistance in the model nematode Caenorhabditis elegans. *Int. J. Parasitol.* **2009**, *39*, 213–220. https://doi.org/10.1016/j.ijpara.2008.06.009.
74. Lespine, A.; Dupuy, J.; Orlowski, S.; Nagy, T.; Glavinas, H.; Krajcsi, P.; Alvinerie, M. Interaction of ivermectin with multidrug resistance proteins (MRP1, 2 and 3). *Chem. Biol. Interact.* **2006**, *159*, 169–179. https://doi.org/10.1016/j.cbi.2005.11.002.
75. Chewchuk, S.; Boorman, T.; Edwardson, D.; Parissenti, A.M. Bile Acids Increase Doxorubicin Sensitivity in ABCC1-expressing Tumour Cells. *Sci. Rep.* **2018**, *8*, 5413. https://doi.org/10.1038/s41598-018-23496-y.
76. Sharma, A.; Biswas, S. Stage-specific cytosolic protein kinase C-like activity in human malarial parasite Plasmodium falciparum. *Indian J. Biochem. Biophys.* **2005**, *42*, 145–151.
77. Ellis, C.; Nathwani, B.; Morrice, N.; Parker, P.; Evans, F.J.; Aitken, A. Ivermectin: An Inhibitor of Protein Kinase C—A Potential Target Enzyme for Onchocerciasis Chemotherapy. *J. Pharm. Pharmacol.* **1985**, *37*, 22. https://doi.org/10.1111/j.2042-7158.1985.tb14094.x.
78. Quilty, F.; Freeley, M.; Gargan, S.; Gilmer, J.; Long, A. Deoxycholic acid induces proinflammatory cytokine production by model oesophageal cells via lipid rafts. *J. Steroid Biochem. Mol. Biol.* **2021**, *214*, 105987. https://doi.org/10.1016/j.jsbmb.2021.105987.
79. Choi, D.S.; Blanco, E.; Kim, Y.S.; Rodriguez, A.A.; Zhao, H.; Huang, T.H.; Chen, C.L.; Jin, G.; Landis, M.D.; Burey, L.A.; et al. Chloroquine eliminates cancer stem cells through deregulation of Jak2 and DNMT1. *Stem Cells* **2014**, *32*, 2309–2323. https://doi.org/10.1002/stem.1746.
80. Manic, G.; Obrist, F.; Kroemer, G.; Vitale, I.; Galluzzi, L. Chloroquine and hydroxychloroquine for cancer therapy. *Mol. Cell Oncol.* **2014**, *1*, e29911. https://doi.org/10.4161/mco.29911.
81. Feng, Y.; Wang, J.; Cai, B.; Bai, X.; Zhu, Y. Ivermectin accelerates autophagic death of glioma cells by inhibiting glycolysis through blocking GLUT4 mediated JAK/STAT signaling pathway activation. *Environ. Toxicol.* **2022**, *37*, 754–764. https://doi.org/10.1002/tox.23440.
82. Pérez-Plasencia, C.; López-Urrutia, E.; García-Castillo, V.; Trujano-Camacho, S.; López-Camarillo, C.; Campos-Parra, A.D. Interplay Between Autophagy and Wnt/β-Catenin Signaling in Cancer: Therapeutic Potential Through Drug Repositioning. *Front. Oncol.* **2020**, *10*, 1037. https://doi.org/10.3389/fonc.2020.01037.

83. Jang, C.H.; Choi, J.H.; Byun, M.S.; Jue, D.M. Chloroquine inhibits production of TNF-alpha, IL-1beta and IL-6 from lipopolysaccharide-stimulated human monocytes/macrophages by different modes. *Rheumatology* **2006**, *45*, 703–710. https://doi.org/10.1093/rheumatology/kei282.
84. Thibaut de Ménonville, S.; Rosignoli, C.; Soares, E.; Roquet, M.; Bertino, B.; Chappuis, J.P.; Defoin-Platel/Chaussade, C.; Piwnica, D. Topical Treatment of Rosacea with Ivermectin Inhibits Gene Expression of Cathelicidin Innate Immune Mediators, LL-37 and KLK5, in Reconstructed and Ex Vivo Skin Models. *Dermatol. Ther.* **2017**, *7*, 213–225. https://doi.org/10.1007/s13555-017-0176-3.
85. Almudaris, S.A.; Gatea, F.K. Effects of topical Ivermectin on imiquimod-induced Psoriasis in mouse model—Novel findings. *Pharmacia* **2024**, *71*, 1–14. https://doi.org/10.3897/pharmacia.71.e114753.
86. Lee, D.K.; Park, S.Y.; Baik, S.K.; Kwon, S.O.; Chung, J.M.; Oh, E.S.; Kim, H.S. [Deoxycholic acid-induced signal transduction in HT-29 cells: Role of NF-kappa B and interleukin-8]. *Korean J. Gastroenterol.* **2004**, *43*, 176–185. (In Korean)
87. Fawcett, J.; Rabkin, R. The processing of insulin-like growth factor-I (IGF-I) by a cultured kidney cell line is altered by IGF-binding protein-3. *Endocrinology* **1995**, *136*, 1340–1347. https://doi.org/10.1210/endo.136.4.7534695.
88. Stankiewicz, M.; Cabaj, W.; Jonas, W.E.; Moore, L.G.; Millar, K.; Ng Chie, W. Influence of ivermectin on cellular and humoral immune responses of lambs. *Vet. Immunol. Immunopathol.* **1995**, *44*, 347–358. https://doi.org/10.1016/0165-2427(94)05308-f.
89. Morgan, S.S. The Bile Acid, Deoxycholic Acid, Modulates IGF-IR Function in Colon Cancer Cells. Ph.D. Thesis, The University of Arizona, Tucson, AZ, USA, 2009. Available online: https://repository.arizona.edu/handle/10150/194122.
90. Zheng, S.; Wang, S.; Zhang, Q.; Zhang, Z.; Xu, S. Avermectin inhibits neutrophil extracellular traps release by activating PTEN demethylation to negatively regulate the PI3K-ERK pathway and reducing respiratory burst in carp. *J. Hazard. Mater.* **2020**, *389*, 121885. https://doi.org/10.1016/j.jhazmat.2019.121885.
91. Yuan, T.; Ni, Z.; Han, C.; Min, Y.; Sun, N.; Liu, C.; Shi, M.; Lu, W.; Wang, N.; Du, F.; et al. SOX2 interferes with the function of CDX2 in bile acid-induced gastric intestinal metaplasia. *Cancer Cell Int.* **2019**, *19*, 24. https://doi.org/10.1186/s12935-019-0739-8.
92. Nguyen, T.T.; Ung, T.T.; Li, S.; Sah, D.K.; Park, S.Y.; Lian, S.; Jung, Y.D. Lithocholic Acid Induces miR21, Promoting PTEN Inhibition via STAT3 and ERK-1/2 Signaling in Colorectal Cancer Cells. *Int. J. Mol. Sci.* **2021**, *22*, 10209. https://doi.org/10.3390/ijms221910209.
93. Sharma, A.; Mishra, N.C. Inhibition of a protein tyrosine kinase activity in Plasmodium falciparum by chloroquine. *Indian J. Biochem. Biophys.* **1999**, *36*, 299–304.
94. Wang, J.; Xu, Y.; Wan, H.; Hu, J. Antibiotic ivermectin selectively induces apoptosis in chronic myeloid leukemia through inducing mitochondrial dysfunction and oxidative stress. *Biochem. Biophys. Res. Commun.* **2018**, *497*, 241–247. https://doi.org/10.1016/j.bbrc.2018.02.063.
95. Khare, S.; Holgren, C.; Samarel, A.M. Deoxycholic acid differentially regulates focal adhesion kinase phosphorylation: Role of tyrosine phosphatase ShP2. *Am. J. Physiol. Gastrointest. Liver Physiol.* **2006**, *291*, G1100–G1112. https://doi.org/10.1152/ajpgi.00008.2006.
96. Zaidi, A.U.; McDonough, J.S.; Klocke, B.J.; Latham, C.B.; Korsmeyer, S.J.; Flavell, R.A.; Schmidt, R.E.; Roth, K.A. Chloroquine-induced neuronal cell death is p53 and Bcl-2 family-dependent but caspase-independent. *J. Neuropathol. Exp. Neurol.* **2001**, *60*, 937–945. https://doi.org/10.1093/jnen/60.10.937.
97. LaRue, J.M.; Stratagoules, E.D.; Martinez, J.D. Deoxycholic acid-induced apoptosis is switched to necrosis by bcl-2 and calphostin C. *Cancer Lett.* **2000**, *152*, 107–113. https://doi.org/10.1016/s0304-3835(99)00439-5.
98. Yui, S.; Kanamoto, R.; Saeki, T. Deoxycholic acid can induce apoptosis in the human colon cancer cell line HCT116 in the absence of Bax. *Nutr. Cancer* **2008**, *60*, 91–96. https://doi.org/10.1080/01635580701525893.
99. Schmukler, E.; Wolfson, E.; Haklai, R.; Elad-Sfadia, G.; Kloog, Y.; Pinkas-Kramarski, R. Chloroquine synergizes with FTS to enhance cell growth inhibition and cell death. *Oncotarget* **2014**, *5*, 173–184. https://doi.org/10.18632/oncotarget.1500.
100. Chen, L.; Bi, S.; Wei, Q.; Zhao, Z.; Wang, C.; Xie, S. Ivermectin suppresses tumour growth and metastasis through degradation of PAK1 in oesophageal squamous cell carcinoma. *J. Cell. Mol. Med.* **2020**, *24*, 5387–5401. https://doi.org/10.1111/jcmm.15195.
101. Eloranta, K.; Cairo, S.; Liljeström, E.; Soini, T.; Kyrönlahti, A.; Judde, J.G.; Wilson, D.B.; Heikinheimo, M.; Pihlajoki, M. Chloroquine Triggers Cell Death and Inhibits PARPs in Cell Models of Aggressive Hepatoblastoma. *Front. Oncol.* **2020**, *10*, 1138. https://doi.org/10.3389/fonc.2020.01138.
102. Zhang, P.; Zhang, Y.; Liu, K.; Liu, B.; Xu, W.; Gao, J.; Ding, L.; Tao, L. Ivermectin induces cell cycle arrest and apoptosis of HeLa cells via mitochondrial pathway. *Cell Prolif.* **2019**, *52*, e12543. https://doi.org/10.1111/cpr.12543.
103. Szántó, M.; Gupte, R.; Kraus, W.L.; Pacher, P.; Bai, P. PARPs in lipid metabolism and related diseases. *Prog. Lipid Res.* **2021**, *84*, 101117. https://doi.org/10.1016/j.plipres.2021.101117.

104. Plantone, D.; Koudriavtseva, T. Current and Future Use of Chloroquine and Hydroxychloroquine in Infectious, Immune, Neoplastic, and Neurological Diseases: A Mini-Review. *Clin. Drug Investig.* **2018**, *38*, 653–671. https://doi.org/10.1007/s40261-018-0656-y.
105. Juarez, M.; Schcolnik-Cabrera, A.; Dominguez-Gomez, G.; Chavez-Blanco, A.; Diaz-Chavez, J.; Duenas-Gonzalez, A. Antitumor effects of ivermectin at clinically feasible concentrations support its clinical development as a repositioned cancer drug. *Cancer Chemother. Pharmacol.* **2020**, *85*, 1153–1163. https://doi.org/10.1007/s00280-020-04041-z.
106. Qiao, D.; Gaitonde, S.V.; Qi, W.; Martinez, J.D. Deoxycholic acid suppresses p53 by stimulating proteasome-mediated p53 protein degradation. *Carcinogenesis* **2001**, *22*, 957–964. https://doi.org/10.1093/carcin/22.6.957.
107. Rahman, M.A.; Park, M.N.; Rahman, M.H.; Rashid, M.M.; Islam, R.; Uddin, M.J.; Hannan, M.A.; Kim, B. p53 Modulation of Autophagy Signaling in Cancer Therapies: Perspectives Mechanism and Therapeutic Targets. *Front. Cell Dev. Biol.* **2022**, *10*, 761080. https://doi.org/10.3389/fcell.2022.761080.
108. Song, D.; Liang, H.; Qu, B.; Li, Y.; Liu, J.; Zhang, Y.; Li, L.; Hu, L.; Zhang, X.; Gao, A. Ivermectin inhibits the growth of glioma cells by inducing cell cycle arrest and apoptosis in vitro and in vivo. *J. Cell. Biochem.* **2019**, *120*, 622–633. https://doi.org/10.1002/jcb.27420.
109. Roman, S.; Pétré, A.; Thépot, A.; Hautefeuille, A.; Scoazec, J.Y.; Mion, F.; Hainaut, P. Downregulation of p63 upon exposure to bile salts and acid in normal and cancer esophageal cells in culture. *Am. J. Physiol. Gastrointest. Liver Physiol.* **2007**, *293*, G45–G53. https://doi.org/10.1152/ajpgi.00583.2006.
110. Yan, T.; Yan, N.; Wang, H.; Yagai, T.; Luo, Y.; Takahashi, S.; Zhao, M.; Krausz, K.W.; Wang, G.; Hao, H.; Gonzalez, F.J. FXR-Deoxycholic Acid-TNF-α Axis Modulates Acetaminophen-Induced Hepatotoxicity. *Toxicol. Sci.* **2021**, *181*, 273–284. https://doi.org/10.1093/toxsci/kfab027.
111. Kim, E.L.; Wüstenberg, R.; Rübsam, A.; Schmitz-Salue, C.; Warnecke, G.; Bücker, E.M.; Pettkus, N.; Speidel, D.; Rohde, V.; Schulz-Schaeffer, W.; et al. Chloroquine activates the p53 pathway and induces apoptosis in human glioma cells. *Neuro Oncol.* **2010**, *12*, 389–400. https://doi.org/10.1093/neuonc/nop046.
112. Zhou, S.; Wu, H.; Ning, W.; Wu, X.; Xu, X.; Ma, Y.; Li, X.; Hu, J.; Wang, C.; Wang, J. Ivermectin has New Application in Inhibiting Colorectal Cancer Cell Growth. *Front. Pharmacol.* **2021**, *12*, 717529. https://doi.org/10.3389/fphar.2021.717529.
113. Jawad, M.J.; Richardson, A. Ivermectin Augments the Anti-Cancer Activity of Pitavastatin in Ovarian Cancer Cells. *Diseases* **2023**, *11*, 49. https://doi.org/10.3390/diseases11010049.
114. Sulik, M.; Otto-Ślusarczyk, D.; Antoszczak, M.; Struga, M.; Huczyński, A. Ivermectin and its synthetic derivatives—A new class of anticancer agents. *Eur. J. Med. Chem. Rep.* **2024**, *12*, 100176. https://doi.org/10.1016/j.ejmcr.2024.100176.
115. Qiao, L.; Studer, E.; Leach, K.; McKinstry, R.; Gupta, S.; Decker, R.; Kukreja, R.; Valerie, K.; Nagarkatti, P.; El Deiry, W.; et al. Deoxycholic acid (DCA) causes ligand-independent activation of epidermal growth factor receptor (EGFR) and FAS receptor in primary hepatocytes: Inhibition of EGFR/mitogen-activated protein kinase-signaling module enhances DCA-induced apoptosis. *Mol. Biol. Cell* **2001**, *12*, 2629–2645. https://doi.org/10.1091/mbc.12.9.2629.
116. Yang, M.; Huang, L.; Li, X.; Kuang, E. Chloroquine inhibits lytic replication of Kaposi's sarcoma-associated herpesvirus by disrupting mTOR and p38-MAPK activation. *Antivir. Res.* **2016**, *133*, 223–233. https://doi.org/10.1016/j.antiviral.2016.08.010.
117. Fang, H.; Liu, A.; Dahmen, U.; Dirsch, O. Dual role of chloroquine in liver ischemia reperfusion injury: Reduction of liver damage in early phase, but aggravation in late phase. *Cell Death Dis.* **2013**, *4*, e694. https://doi.org/10.1038/cddis.2013.225.
118. Shan, J.; Oshima, T.; Fukui, H.; Watari, J.; Miwa, H. Acidic deoxycholic acid and chenodeoxycholic acid induce interleukin-8 production through p38 mitogen-activated protein kinase and protein kinase A in a squamous epithelial model. *J. Gastroenterol. Hepatol.* **2013**, *28*, 823–828. https://doi.org/10.1111/jgh.12139.
119. Looby, E.; Abdel-Latif, M.M.; Athié-Morales, V.; Duggan, S.; Long, A.; Kelleher, D. Deoxycholate induces COX-2 expression via Erk1/2-, p38-MAPK and AP-1-dependent mechanisms in esophageal cancer cells. *BMC Cancer* **2009**, *9*, 190. https://doi.org/10.1186/1471-2407-9-190.
120. Chiou, J.T.; Lee, Y.C.; Chang, L.S. Hydroquinone-selected chronic myelogenous leukemia cells are sensitive to chloroquine-induced cytotoxicity via MCL1 suppression and glycolysis inhibition. *Biochem. Pharmacol.* **2023**, *218*, 115934. https://doi.org/10.1016/j.bcp.2023.115934.
121. Lee, D.E.; Kang, H.W.; Kim, S.Y.; Kim, M.J.; Jeong, J.W.; Hong, W.C.; Fang, S.; Kim, H.S.; Lee, Y.S.; Kim, H.J.; et al. Ivermectin and gemcitabine combination treatment induces apoptosis of pancreatic cancer cells via mitochondrial dysfunction. *Front. Pharmacol.* **2022**, *13*, 934746. https://doi.org/10.3389/fphar.2022.934746.
122. Liao, M.; Zhao, J.; Wang, T.; Duan, J.; Zhang, Y.; Deng, X. Role of bile salt in regulating Mcl-1 phosphorylation and chemoresistance in hepatocellular carcinoma cells. *Mol. Cancer* **2011**, *10*, 44. https://doi.org/10.1186/1476-4598-10-44.

123. Weber, S.M.; Chen, J.M.; Levitz, S.M. Inhibition of mitogen-activated protein kinase signaling by chloroquine. *J. Immunol.* **2002**, *168*, 5303–5309. https://doi.org/10.4049/jimmunol.168.10.5303.
124. Park, E.J.; Min, K.J.; Choi, K.S.; Kubatka, P.; Kruzliak, P.; Kim, D.E.; Kwon, T.K. Chloroquine enhances TRAIL-mediated apoptosis through up-regulation of DR5 by stabilization of mRNA and protein in cancer cells. *Sci. Rep.* **2016**, *6*, 22921. https://doi.org/10.1038/srep22921.
125. Monma, H.; Iida, Y.; Moritani, T.; Okimoto, T.; Tanino, R.; Tajima, Y.; Harada, M. Chloroquine augments TRAIL-induced apoptosis and induces G2/M phase arrest in human pancreatic cancer cells. *PLoS ONE* **2018**, *13*, e0193990. https://doi.org/10.1371/journal.pone.0193990.
126. Helmy, S.A.; El-Mesery, M.; El-Karef, A.; Eissa, L.A.; El Gayar, A.M. Chloroquine upregulates TRAIL/TRAILR2 expression and potentiates doxorubicin anti-tumor activity in thioacetamide-induced hepatocellular carcinoma model. *Chem. Biol. Interact.* **2018**, *279*, 84–94. https://doi.org/10.1016/j.cbi.2017.11.009.
127. Jia, B.; Xue, Y.; Yan, X.; Li, J.; Wu, Y.; Guo, R.; Zhang, J.; Zhang, L.; Li, Y.; Liu, Y.; Sun, L. Autophagy inhibitor chloroquine induces apoptosis of cholangiocarcinoma cells via endoplasmic reticulum stress. *Oncol. Let.* **2018**, *16*, 3509–3516. https://doi.org/10.3892/ol.2018.9131.
128. Higuchi, H.; Grambihler, A.; Canbay, A.; Bronk, S.F.; Gores, G.J. Bile acids up-regulate death receptor 5/TRAIL-receptor 2 expression via a c-Jun N-terminal kinase-dependent pathway involving Sp1. *J. Biol. Chem.* **2004**, *279*, 51–60. https://doi.org/10.1074/jbc.M309476200.
129. Mauthe, M.; Orhon, I.; Rocchi, C.; Zhou, X.; Luhr, M.; Hijlkema, K.J.; Coppes, R.P.; Engedal, N.; Mari, M.; Reggiori, F. Chloroquine inhibits autophagic flux by decreasing autophagosome-lysosome fusion. *Autophagy* **2018**, *14*, 1435–1455. https://doi.org/10.1080/15548627.2018.1474314.
130. Ye, H.; Chen, M.; Cao, F.; Huang, H.; Zhan, R.; Zheng, X. Chloroquine, an autophagy inhibitor, potentiates the radiosensitivity of glioma initiating cells by inhibiting autophagy and activating apoptosis. *BMC Neurol.* **2016**, *16*, 178. https://doi.org/10.1186/s12883-016-0700-6.
131. Redmann, M.; Benavides, G.A.; Berryhill, T.F.; Wani, W.Y.; Ouyang, X.; Johnson, M.S.; Ravi, S.; Barnes, S.; Darley-Usmar, V.M.; Zhang, J. Inhibition of autophagy with bafilomycin and chloroquine decreases mitochondrial quality and bioenergetic function in primary neurons. *Redox Biol.* **2017**, *11*, 73–81. https://doi.org/10.1016/j.redox.2016.11.004.
132. Li, M.Y.; Zhang, J.; Lu, X.; Zhou, D.; Deng, X.F.; Liu, Q.X.; Dai, J.G.; Zheng, H. Ivermectin induces nonprotective autophagy by downregulating PAK1 and apoptosis in lung adenocarcinoma cells. *Cancer Chemother. Pharmacol.* **2024**, *93*, 41–54. https://doi.org/10.1007/s00280-023-04589-6.
133. Dalle Pezze, P.; Karanasios, E.; Kandia, V.; Manifava, M.; Walker, S.A.; Gambardella Le Novère, N.; Ktistakis, N.T. ATG13 dynamics in nonselective autophagy and mitophagy: Insights from live imaging studies and mathematical modeling. *Autophagy* **2021**, *17*, 1131–1141. https://doi.org/10.1080/15548627.2020.1749401.
134. Payne, C.M.; Crowley-Skillicorn, C.; Holubec, H.; Dvorak, K.; Bernstein, C.; Moyer, M.P.; Garewal, H.; Bernstein, H. Deoxycholate, an endogenous cytotoxin/genotoxin, induces the autophagic stress-survival pathway: Implications for colon carcinogenesis. *J. Toxicol.* **2009**, *2009*, 785907. https://doi.org/10.1155/2009/785907.
135. Panzitt, K.; Fickert, P.; Wagner, M. Regulation of autophagy by bile acids and in cholestasis—CholestoPHAGY or CholeSTOPagy. *Biochim. Biophys. Acta (BBA)-Mol. Basis Dis.* **2021**, *1867*, 166017. https://doi.org/10.1016/j.bbadis.2020.166017.
136. Qu, X.; Sheng, J.; Shen, L.; Su, J.; Xu, Y.; Xie, Q.; Wu, Y.; Zhang, X.; Sun, L. Autophagy inhibitor chloroquine increases sensitivity to cisplatin in QBC939 cholangiocarcinoma cells by mitochondrial ROS. *PLoS ONE* **2017**, *12*, e0173712. https://doi.org/10.1371/journal.pone.0173712.
137. Zhang, P.; Ni, H.; Zhang, Y.; Xu, W.; Gao, J.; Cheng, J.; Tao, L. Ivermectin confers its cytotoxic effects by inducing AMPK/mTOR-mediated autophagy and DNA damage. *Chemosphere* **2020**, *259*, 127448. https://doi.org/10.1016/j.chemosphere.2020.127448.
138. Roesly, H.B.; Khan, M.R.; Chen, H.D.; Hill, K.A.; Narendran, N.; Watts, G.S.; Chen, X.; Dvorak, K. The decreased expression of Beclin-1 correlates with progression to esophageal adenocarcinoma: The role of deoxycholic acid. *Am. J. Physiol. Gastrointest. Liver Physiol.* **2012**, *302*, G864–G872. https://doi.org/10.1152/ajpgi.00340.2011.
139. Grimaldi, A.; Santini, D.; Zappavigna, S.; Lombardi, A.; Misso, G.; Boccellino, M.; Desiderio, V.; Vitiello, P.P.; Di Lorenzo, G.; Zoccoli, A.; et al. Antagonistic effects of chloroquine on autophagy occurrence potentiate the anticancer effects of everolimus on renal cancer cells. *Cancer Biol. Ther.* **2015**, *16*, 567–579. https://doi.org/10.1080/15384047.2015.1018494.

140. Silva, R.C.M.C.; Tan, L.; Rodrigues, D.A.; Prestes, E.B.; Gomes, C.P.; Gama, A.M.; Oliveira, P.L.; Paiva, C.N.; Manoury, B.; Bozza, M.T. Chloroquine inhibits pro-inflammatory effects of heme on macrophages and in vivo. *Free Radic. Biol. Med.* **2021**, *173*, 104–116. https://doi.org/10.1016/j.freeradbiomed.2021.07.028.
141. Iglesias-Corral, D.; García-Valles, P.; Arroyo-Garrapucho, N.; Bueno-Martínez, E.; Ruiz-Robles, J.M.; Ovejero-Sánchez, M.; González-Sarmiento, R.; Herrero, A.B. Chloroquine-induced DNA damage synergizes with DNA repair inhibitors causing cancer cell death. *Front. Oncol.* **2024**, *14*, 1390518. https://doi.org/10.3389/fonc.2024.1390518.
142. Fan, N.; Zhang, L.; Wang, Z.; Ding, H.; Yue, Z. Ivermectin Inhibits Bladder Cancer Cell Growth and Induces Oxidative Stress and DNA Damage. *Anticancer Agents Med. Chem.* **2024**, *24*, 348–357. https://doi.org/10.2174/0118715206274095231106042833.
143. Fang, Y.; Han, S.I.; Mitchell, C.; Gupta, S.; Studer, E.; Grant, S.; Hylemon, P.B.; Dent, P. Bile acids induce mitochondrial ROS, which promote activation of receptor tyrosine kinases and signaling pathways in rat hepatocytes. *Hepatology* **2004**, *40*, 961–971. https://doi.org/10.1002/hep.1840400427.
144. Jenkins, G.J.; Cronin, J.; Alhamdani, A.; Rawat, N.; D'Souza, F.; Thomas, T.; Eltahir, Z.; Griffiths, A.P.; Baxter, J.N. The bile acid deoxycholic acid has a non-linear dose response for DNA damage and possibly NF-kappaB activation in oesophageal cells, with a mechanism of action involving ROS. *Mutagenesis* **2008**, *23*, 399–405. https://doi.org/10.1093/mutage/gen029.
145. Schwarcz, S.; Kovács, P.; Kovács, T.; Ujlaki, G.; Nyerges, P.; Uray, K.; Bai, P.; Mikó, E. The pro- and antineoplastic effects of deoxycholic acid in pancreatic adenocarcinoma cell models. *Mol. Biol. Rep.* **2023**, *50*, 5273–5282. https://doi.org/10.1007/s11033-023-08453-x.
146. Zhang, Y.; Li, Y.; Li, Y.; Li, R.; Ma, Y.; Wang, H.; Wang, Y. Chloroquine inhibits MGC803 gastric cancer cell migration via the Toll-like receptor 9/nuclear factor kappa B signaling pathway. *Mol. Med. Rep.* **2015**, *11*, 1366–1371. https://doi.org/10.3892/mmr.2014.2839.
147. Bouchard, G.; Therriault, H.; Geha, S.; Bérubé-Lauzière, Y.; Bujold, R.; Saucier, C.; Paquette, B. Stimulation of triple negative breast cancer cell migration and metastases formation is prevented by chloroquine in a pre-irradiated mouse model. *BMC Cancer* **2016**, *16*, 361. https://doi.org/10.1186/s12885-016-2393-z.
148. Zhang, X.; Song, Y.; Xiong, H.; Ci, X.; Li, H.; Yu, L.; Zhang, L.; Deng, X. Inhibitory effects of ivermectin on nitric oxide and prostaglandin E2 production in LPS-stimulated RAW 264.7 macrophages. *Int. Immunopharmacol.* **2009**, *9*, 354–359. https://doi.org/10.1016/j.intimp.2008.12.016.
149. Aryannejad, A.; Tabary, M.; Noroozi, N.; Mashinchi, B.; Iranshahi, S.; Tavangar, S.M.; Mohammad Jafari, R.; Rashidian, A.; Dehpour, A.R. Anti-inflammatory Effects of Ivermectin in the Treatment of Acetic Acid-Induced Colitis in Rats: Involvement of $GABA_B$ Receptors. *Dig. Dis. Sci.* **2022**, *67*, 3672–3682. https://doi.org/10.1007/s10620-021-07258-x.
150. Yoon, J.H.; Higuchi, H.; Werneburg, N.W.; Kaufmann, S.H.; Gores, G.J. Bile acids induce cyclooxygenase-2 expression via the epidermal growth factor receptor in a human cholangiocarcinoma cell line. *Gastroenterology* **2002**, *122*, 985–993. https://doi.org/10.1053/gast.2002.32410.
151. Zhu, Y.; Zhu, M.; Lance, P. Stromal COX-2 signaling activated by deoxycholic acid mediates proliferation and invasiveness of colorectal epithelial cancer cells. *Biochem. Biophys. Res. Commun.* **2012**, *425*, 607–612. https://doi.org/10.1016/j.bbrc.2012.07.137.
152. Sheng, J.; Sun, H.; Yu, F.B.; Li, B.; Zhang, Y.; Zhu, Y.T. The Role of Cyclooxygenase-2 in Colorectal Cancer. *Int. J. Med. Sci.* **2020**, *17*, 1095–1101. https://doi.org/10.7150/ijms.44439.
153. Park, Y.C.; Pae, H.O.; Yoo, J.C.; Choi, B.M.; Jue, D.M.; Chung, H.T. Chloroquine inhibits inducible nitric oxide synthase expression in murine peritoneal macrophages. *Pharmacol. Toxicol.* **1999**, *85*, 188–191. https://doi.org/10.1111/j.1600-0773.1999.tb00090.x.
154. McAdam, E.; Haboubi, H.N.; Forrester, G.; Eltahir, Z.; Spencer-Harty, S.; Davies, C.; Griffiths, A.P.; Baxter, J.N.; Jenkins, G.J. Inducible nitric oxide synthase (iNOS) and nitric oxide (NO) are important mediators of reflux-induced cell signalling in esophageal cells. *Carcinogenesis* **2012**, *33*, 2035–2043. https://doi.org/10.1093/carcin/bgs241.
155. Ertel, W.; Morrison, M.H.; Ayala, A.; Chaudry, I.H. Chloroquine attenuates hemorrhagic shock-induced suppression of Kupffer cell antigen presentation and major histocompatibility complex class II antigen expression through blockade of tumor necrosis factor and prostaglandin release. *Blood* **1991**, *78*, 1781–1788. https://doi.org/10.1182/blood.V78.7.1781.1781.
156. Manku, M.S.; Horrobin, D.F. Chloroquine, quinine, procaine, quinidine and clomipramine are prostaglandin agonists and antagonists. *Prostaglandins* **1976**, *12*, 789–801. https://doi.org/10.1016/0090-6980(76)90053-8.
157. Zhu, Y.; Hua, P.; Rafiq, S.; Waffner, E.J.; Duffey, M.E.; Lance, P. Ca^{2+}- and PKC-dependent stimulation of PGE_2 synthesis by deoxycholic acid in human colonic fibroblasts. *Am. J. Physiol. Gastrointest. Liver Physiol.* **2002**, *283*, G503–G510. https://doi.org/10.1152/ajpgi.00525.2001.

158. Liu, Y.; Meng, Y.; Zhang, J.; Gu, L.; Shen, S.; Zhu, Y.; Wang, J. Pharmacology Progresses and Applications of Chloroquine in Cancer Therapy. *Int. J. Nanomed.* **2024**, *19*, 6777–6809. https://doi.org/10.2147/IJN.S458910.
159. Liu, Y.; Fang, S.; Sun, Q.; Liu, B. Anthelmintic drug ivermectin inhibits angiogenesis, growth and survival of glioblastoma through inducing mitochondrial dysfunction and oxidative stress. *Biochem. Biophys. Res. Commun.* **2016**, *480*, 415–421. https://doi.org/10.1016/j.bbrc.2016.10.064.
160. Song, X.; An, Y.; Chen, D.; Zhang, W.; Wu, X.; Li, C.; Wang, S.; Dong, W.; Wang, B.; Liu, T.; et al. Microbial metabolite deoxycholic acid promotes vasculogenic mimicry formation in intestinal carcinogenesis. *Cancer Sci.* **2022**, *113*, 459–477. https://doi.org/10.1111/cas.15208.
161. Kang, C.; Ju, S.; Kim, J.; Jung, Y. Chloroquine prevents hypoxic accumulation of HIF-1α by inhibiting ATR kinase: Implication in chloroquine-mediated chemosensitization of colon carcinoma cells under hypoxia. *Pharmacol. Rep.* **2023**, *75*, 211–221. https://doi.org/10.1007/s43440-022-00441-5.
162. Kosyna, F.K.; Nagel, M.; Kluxen, L.; Kraushaar, K.; Depping, R. The importin α/β-specific inhibitor Ivermectin affects HIF-dependent hypoxia response pathways. *Biol. Chem.* **2015**, *396*, 1357–1367. https://doi.org/10.1515/hsz-2015-0171.
163. Wang, C.; Chu, Q.; Dong, W.; Wang, X.; Zhao, W.; Dai, X.; Liu, W.; Wang, B.; Liu, T.; Zhong, W.; et al. Microbial metabolite deoxycholic acid-mediated ferroptosis exacerbates high-fat diet-induced colonic inflammation. *Mol. Metab.* **2024**, *84*, 101944. https://doi.org/10.1016/j.molmet.2024.101944.
164. Lee, S.Y.; Kim, S.J.; Park, K.H.; Lee, G.; Oh, Y.; Ryu, J.-H.; Huh, Y.H. Differential but complementary roles of HIF-1α and HIF-2α in the regulation of bone homeostasis. *Commun. Biol.* **2024**, *7*, 892. https://doi.org/10.1038/s42003-024-06581-z.
165. Lesiak, A.; Narbutt, J.; Kobos, J.; Kordek, R.; Sysa-Jedrzejowska, A.; Norval, M.; Wozniacka, A. Systematic administration of chloroquine in discoid lupus erythematosus reduces skin lesions via inhibition of angiogenesis. *Clin. Exp. Dermatol.* **2009**, *34*, 570–575. https://doi.org/10.1111/j.1365-2230.2008.03006.x.
166. Liu, J.; Zhang, K.; Cheng, L.; Zhu, H.; Xu, T. Progress in Understanding the Molecular Mechanisms Underlying the Antitumour Effects of Ivermectin. *Drug Des. Dev. Ther.* **2020**, *14*, 285–296. https://doi.org/10.2147/DDDT.S237393.
167. Siddiqui, A.J.; Khan, M.F.; Hamadou, W.S.; Goyal, M.; Jahan, S.; Jamal, A.; Ashraf, S.A.; Sharma, P.; Sachidanandan, M.; Badraoui, R.; et al. Molecular Docking and Dynamics Simulation Revealed Ivermectin as Potential Drug against Schistosoma-Associated Bladder Cancer Targeting Protein Signaling: Computational Drug Repositioning Approach. *Medicina* **2021**, *57*, 1058. https://doi.org/10.3390/medicina57101058.
168. Mahmoud, M.A.A.; Saleh, D.O.; Safar, M.M.; Agha, A.M.; Khattab, M.M. Chloroquine ameliorates bone loss induced by d-galactose in male rats via inhibition of ERK associated osteoclastogenesis and antioxidant effect. *Toxicol. Rep.* **2021**, *8*, 366–375. https://doi.org/10.1016/j.toxrep.2021.02.007.
169. Draganov, D.; Han, Z.; Rana, A.; Bennett, N.; Irvine, D.J.; Lee, P.P. Ivermectin converts cold tumors hot and synergizes with immune checkpoint blockade for treatment of breast cancer. *NPJ Breast Cancer* **2021**, *7*, 22. https://doi.org/10.1038/s41523-021-00229-5.
170. Chen, X.; Mellon, R.D.; Yang, L.; Dong, H.; Oppenheim, J.J.; Howard, O.M. Regulatory effects of deoxycholic acid, a component of the anti-inflammatory traditional Chinese medicine Niuhuang, on human leukocyte response to chemoattractants. *Biochem. Pharmacol.* **2002**, *63*, 533–541. https://doi.org/10.1016/s0006-2952(01)00917-0.
171. Zhou, Q.; Yang, X.; Xiong, M.; Xu, X.; Zhen, L.; Chen, W.; Wang, Y.; Shen, J.; Zhao, P.; Liu, Q.H. Chloroquine Increases Glucose Uptake via Enhancing GLUT4 Translocation and Fusion with the Plasma Membrane in L6 Cells. *Cell Physiol. Biochem.* **2016**, *38*, 2030–2040. https://doi.org/10.1159/000445562.
172. Byrne, A.M.; Sharma, R.; Duggan, G.; Kelleher, D.; Long, A. Deoxycholic acid impairs glycosylation and fucosylation processes in esophageal epithelial cells. *Glycobiology* **2012**, *22*, 638–648. https://doi.org/10.1093/glycob/cwr190.
173. Wahlström, A.; Brumbaugh, A.; Sjöland, W.; Olsson, L.; Wu, H.; Henricsson, M.; Lundqvist, A.; Makki, K.; Hazen, S.L.; Bergström, G.; et al. Production of deoxycholic acid by low-abundant microbial species is associated with impaired glucose metabolism. *Nat. Commun.* **2024**, *15*, 4276. https://doi.org/10.1038/s41467-024-48543-3.
174. Kuznik, A.; Bencina, M.; Svajger, U.; Jeras, M.; Rozman, B.; Jerala, R. Mechanism of endosomal TLR inhibition by antimalarial drugs and imidazoquinolines. *J. Immunol.* **2011**, *186*, 4794–4804. https://doi.org/10.4049/jimmunol.1000702.
175. Zhu, X.; Pan, Y.; Li, Y.; Jiang, Y.; Shang, H.; Gowda, D.C.; Cui, L.; Cao, Y. Targeting Toll-like receptors by chloroquine protects mice from experimental cerebral malaria. *Int. Immunopharmacol.* **2012**, *13*, 392–397. https://doi.org/10.1016/j.intimp.2012.05.012.
176. Gergen, A.K.; Jarrett, M.J.; Li, A.; Meng, X.; Pratap, A.; Fullerton, D.A.; Weyant, M.J. Toll-like Receptor 4 Mediates Reflux-Induced Inflammation in a Murine Reflux Model. *Semin. Thorac. Cardiovasc. Surg.* **2022**, *34*, 1324–1335. https://doi.org/10.1053/j.semtcvs.2021.07.033.

177. Trelford, C.B.; Dagnino, L.; Di Guglielmo, G.M. Transforming growth factor-β in tumour development. *Front. Mol. Biosci.* **2022**, *9*, 991612. https://doi.org/10.3389/fmolb.2022.991612.
178. Tian, S.; Zheng, Y.; Xiao, S.; Luo, P.; Sun, R.; Liu, J.; Xia, Z. Ivermectin inhibits cell proliferation and the expression levels of type I collagen, α-SMA and CCN2 in hypertrophic scar fibroblasts. *Mol. Med. Rep.* **2021**, *24*, 488. https://doi.org/10.3892/mmr.2021.12127.
179. Hazel, K. The Role of Bile Acids in Regulating Inflammation and Fibrosis in Inflammatory Bowel Disease. Ph.D. Thesis, Royal College of Surgeons in Ireland, Dublin, Ireland, 2022. https://doi.org/10.25419/rcsi.20227386.v1.
180. Güiza, J.; Arriagada, J.; Rodríguez, L.; Gutiérrez, C.; Duarte, Y.; Sáez, J.C.; Vega, J.L. Anti-parasitic drugs modulate the non-selective channels formed by connexins or pannexins. *Biochim. Biophys. Acta Mol. Basis Dis.* **2021**, *1867*, 166188. https://doi.org/10.1016/j.bbadis.2021.166188.
181. Iglesias, R.; Locovei, S.; Roque, A.; Alberto, A.P.; Dahl, G.; Spray, D.C.; Scemes, E. P2X7 receptor-Pannexin1 complex: Pharmacology and signaling. *Am. J. Physiol. Cell Physiol.* **2008**, *295*, C752–C760. https://doi.org/10.1152/ajpcell.00228.2008.
182. Stokes, L.; Surprenant, A. Dynamic regulation of the $P2X_4$ receptor in alveolar macrophages by phagocytosis and classical activation. *Eur. J. Immunol.* **2009**, *39*, 986–995. https://doi.org/10.1002/eji.200838818.
183. Hounjet, J.; Habets, R.; Schaaf, M.B.; Hendrickx, T.C.; Barbeau, L.M.O.; Yahyanejad, S.; Rouschop, K.M.; Groot, A.J.; Vooijs, M. The anti-malarial drug chloroquine sensitizes oncogenic NOTCH1 driven human T-ALL to γ-secretase inhibition. *Oncogene* **2019**, *38*, 5457–5468. https://doi.org/10.1038/s41388-019-0802-x.
184. Li, N.; Zhan, X. Anti-parasite drug ivermectin can suppress ovarian cancer by regulating lncRNA-EIF4A3-mRNA axes. *EPMA J.* **2020**, *11*, 289–309. https://doi.org/10.1007/s13167-020-00209-y.
185. Morrow, D.J.; Avissar, N.E.; Toia, L.; Redmond, E.M.; Watson, T.J.; Jones, C.; Raymond, D.P.; Litle, V.; Peters, J.H. Pathogenesis of Barrett's esophagus: Bile acids inhibit the Notch signaling pathway with induction of CDX2 gene expression in human esophageal cells. *Surgery* **2009**, *146*, 714–722. https://doi.org/10.1016/j.surg.2009.06.050.
186. Dang, C.V. Antimalarial therapy prevents Myc-induced lymphoma. *J. Clin. Investig.* **2008**, *118*, 15–17. https://doi.org/10.1172/JCI34503.
187. Nappi, L.; Aguda, A.H.; Nakouzi, N.A.; Lelj-Garolla, B.; Beraldi, E.; Lallous, N.; Thi, M.; Moore, S.; Fazli, L.; Battsogt, D.; et al. Ivermectin inhibits HSP27 and potentiates efficacy of oncogene targeting in tumor models. *J. Clin. Investig.* **2020**, *130*, 699–714. https://doi.org/10.1172/JCI130819.
188. Tselepis, C.; Morris, C.D.; Wakelin, D.; Hardy, R.; Perry, I.; Luong, Q.T.; Harper, E.; Harrison, R.; Attwood, S.E.; Jankowski, J.A. Upregulation of the oncogene c-myc in Barrett's adenocarcinoma: Induction of c-myc by acidified bile acid in vitro. *Gut* **2003**, *52*, 174–180. https://doi.org/10.1136/gut.52.2.174.
189. Ishibashi, Y.; Nakamura, O.; Yamagami, Y.; Nishimura, H.; Fukuoka, N.; Yamamoto, T. Chloroquine Enhances Rapamycin-induced Apoptosis in MG63 Cells. *Anticancer Res.* **2019**, *39*, 649–654. https://doi.org/10.21873/anticanres.13159.
190. Fu, B.; Lou, Y.; Wu, P.; Lu, X.; Xu, C. Emerging role of necroptosis, pyroptosis, and ferroptosis in breast cancer: New dawn for overcoming therapy resistance. *Neoplasia* **2024**, *55*, 101017. https://doi.org/10.1016/j.neo.2024.101017.
191. Li, Q.; Yuan, D.M.; Ma, L.H.; Ma, C.H.; Liu, Y.F.; Lv, T.F.; Song, Y. Chloroquine inhibits tumor growth and angiogenesis in malignant pleural effusion. *Tumour Biol.* **2016**, *37*, 16249–16258. https://doi.org/10.1007/s13277-016-5441-z.
192. Gurel-Gurevin, E.; Kiyan, H.T.; Esener, O.B.B.; Aydinlik, S.; Uvez, A.; Ulukaya, E.; Dimas, K.; Armutak, E.I. Chloroquine Used in Combination with Chemotherapy Synergistically Suppresses Growth and Angiogenesis In Vitro and In Vivo. *Anticancer Res.* **2018**, *38*, 4011–4020. https://doi.org/10.21873/anticanres.12689.
193. Ferreira, P.M.P.; Sousa, R.W.R.; Ferreira, J.R.O.; Militão, G.C.G.; Bezerra, D.P. Chloroquine and hydroxychloroquine in antitumor therapies based on autophagy-related mechanisms. *Pharmacol. Res.* **2021**, *168*, 105582. https://doi.org/10.1016/j.phrs.2021.105582.
194. Huang, H.; He, Q.; Guo, B.; Xu, X.; Wu, Y.; Li, X. Progress in Redirecting Antiparasitic Drugs for Cancer Treatment. *Drug Des. Dev. Ther.* **2021**, *15*, 2747–2767. https://doi.org/10.2147/DDDT.S308973.
195. Yang, X.; Jin, Z.; Chen, G.; Hu, G. Chloroquine inhibits salinomycin-induced autophagy for collaborative anticancer effect in breast cancer. *Bioimpacts* **2025**, *15*, 30821. https://doi.org/10.34172/bi.30821.
196. Dou, Q.; Chen, H.N.; Wang, K.; Yuan, K.; Lei, Y.; Li, K.; Lan, J.; Chen, Y.; Huang, Z.; Xie, N.; et al. Ivermectin Induces Cytostatic Autophagy by Blocking the PAK1/Akt Axis in Breast Cancer. *Cancer Res.* **2016**, *76*, 4457–4469. https://doi.org/10.1158/0008-5472.CAN-15-2887.

197. Wang, X.; Wang, J.; Zhang, P.; Zhang, C.; Wang, W.; Wu, M.; Xu, W.; Tao, L.; Li, Z.; Zhang, Y. Cytotoxicity and Autophagy Induced by Ivermectin via AMPK/mTOR Signaling Pathway in RAW264.7 Cells. *Molecules* **2023**, *28*, 2201. https://doi.org/10.3390/molecules28052201.
198. Liu, H.; Chai, Z.; Gao, Y.; Wang, Y.; Lu, M. Ivermectin inhibits the growth of ESCC by activating the ATF4-mediated endoplasmic reticulum stress-autophagy pathway. *Acta Biochim. Biophys. Sin.* **2024**, *57*, 995–1005. https://doi.org/10.3724/abbs.2024210.
199. Maruta, H.; He, H. PAK1-blockers: Potential Therapeutics against COVID-19. *Med. Drug Discov.* **2020**, *6*, 100039. https://doi.org/10.1016/j.medidd.2020.100039.
200. Zhao, L.; Au, J.L.; Wientjes, M.G. Comparison of methods for evaluating drug-drug interaction. *Front. Biosci.* **2010**, *2*, 241–249. https://doi.org/10.2741/e86.
201. Feng, H.; He, L.; Umar, T.; Wang, X.; Li, W.; Zhang, B.; Zhu, X.; Deng, G.; Qiu, C. Synergistic Antitumor Effects of Ivermectin and Metformin in Canine Breast Cancer via PI3K/AKT/mTOR Pathway Inhibition. *Curr. Issues Mol. Biol.* **2025**, *47*, 403. https://doi.org/10.3390/cimb47060403.
202. Parks, S.E.; Yustein, J.T. PAK1 and PAK4 as therapeutic targets for Ewing sarcoma: A commentary. *J. Cancer Biol.* **2021**, *2*, 94–97. https://doi.org/10.46439/cancerbiology.2.032.
203. Li, C.F.; Chan, T.C.; Fang, F.M.; Yu, S.C.; Huang, H.Y. PAK1 overexpression promotes myxofibrosarcoma angiogenesis through STAT5B-mediated *CSF2* transactivation: Clinical and therapeutic relevance of amplification and nuclear entry. *Int. J. Biol. Sci.* **2023**, *19*, 3920–3936. https://doi.org/10.7150/ijbs.83467.
204. Mei, L.; Chen, Y.; Wang, Z.; Wang, J.; Wan, J.; Yu, C.; Liu, X.; Li, W. Synergistic anti-tumour effects of tetrandrine and chloroquine combination therapy in human cancer: A potential antagonistic role for p21. *Br. J. Pharmacol.* **2015**, *172*, 2232–2245. https://doi.org/10.1111/bph.13045.
205. Bano, N.; Ansari, M.I.; Kainat, K.M.; Singh, V.K.; Sharma, P.K. Chloroquine synergizes doxorubicin efficacy in cervical cancer cells through flux impairment and down regulation of proteins involved in the fusion of autophagosomes to lysosomes. *Biochem. Biophys. Res. Commun.* **2023**, *656*, 131–138. https://doi.org/10.1016/j.bbrc.2023.03.048.
206. Abdel-Aziz, A.K.; Shouman, S.; El-Demerdash, E.; Elgendy, M.; Abdel-Naim, A.B. Chloroquine synergizes sunitinib cytotoxicity via modulating autophagic, apoptotic and angiogenic machineries. *Chem. Biol. Interact.* **2014**, *217*, 28–40. https://doi.org/10.1016/j.cbi.2014.04.007.
207. Xie, F.; Zhang, S.; Liu, J.; Gong, Z.; Yang, K.; Zhang, H.; Lu, Y.; Zou, H.; Yu, Y.; Chen, Y.; et al. Codelivery of salinomycin and chloroquine by liposomes enables synergistic antitumor activity in vitro. *Nanomedicine* **2016**, *11*, 1831–1846. https://doi.org/10.2217/nnm-2016-0125.
208. Liang, A.L.; Zhang, J.; Du, S.L.; Zhang, B.; Ma, X.; Wu, C.Y.; Liu, Y.J. Chloroquine increases the anti-cancer activity of epirubicin in A549 lung cancer cells. *Oncol. Lett.* **2020**, *20*, 53–60. https://doi.org/10.3892/ol.2020.11567.
209. Duarte, D.; Vale, N. New Trends for Antimalarial Drugs: Synergism between Antineoplastics and Antimalarials on Breast Cancer Cells. *Biomolecules* **2020**, *10*, 1623. https://doi.org/10.3390/biom10121623.
210. Wang, G.; Chen, S.; Edwards, H.; Cui, X.; Cui, L.; Ge, Y. Combination of chloroquine and GX15-070 (obatoclax) results in synergistic cytotoxicity against pancreatic cancer cells. *Oncol. Rep.* **2014**, *32*, 2789–2794. https://doi.org/10.3892/or.2014.3525.
211. Li, M.L.; Xu, Y.Z.; Lu, W.J.; Li, Y.H.; Tan, S.S.; Lin, H.J.; Wu, T.M.; Li, Y.; Wang, S.Y.; Zhao, Y.L. Chloroquine potentiates the anticancer effect of sunitinib on renal cell carcinoma by inhibiting autophagy and inducing apoptosis. *Oncol. Lett.* **2018**, *15*, 2839-2846. https://doi.org/10.3892/ol.2017.7635. Erratum in *Oncol. Lett.* **2024**, *27*, 270. https://doi.org/10.3892/ol.2024.14403.
212. Ryabaya, O.O.; Inshakov, A.N.; Egorova, A.V.; Emelyanova, M.A.; Nasedkina, T.V.; Zasedatelev, A.S.; Khochenkov, D.A.; Stepanova, E.V. Autophagy inhibitors chloroquine and LY294002 enhance temozolomide cytotoxicity on cutaneous melanoma cell lines in vitro. *Anticancer Drugs* **2017**, *28*, 307–315. https://doi.org/10.1097/CAD.0000000000000463.
213. Kang, M.; Lee, K.H.; Lee, H.S.; Wook Jeong, C.; Kwak, C.; Kim, H.H.; Ku, J.H. Concurrent autophagy inhibition overcomes the resistance of epidermal growth factor receptor tyrosine kinase inhibitors in human bladder cancer cells. *Int. J. Mol. Sci.* **2017**, *18*, 321. https://doi.org/10.3390/ijms18020321.
214. Quan, Y.; Lei, H.; Wahafu, W.; Liu, Y.; Ping, H.; Zhang, X. Inhibition of autophagy enhances the anticancer effect of enzalutamide on bladder cancer. *Biomed. Pharmacother.* **2019**, *120*, 109490. https://doi.org/10.1016/j.biopha.2019.109490.
215. Liang, L.; Hui, K.; Hu, C.; Wen, Y.; Yang, S.; Zhu, P.; Wang, L.; Xia, Y.; Qiao, Y.; Sun, W.; et al. Autophagy inhibition potentiates the anti-angiogenic property of multikinase inhibitor anlotinib through JAK2/STAT3/VEGFA signaling in non-small cell lung cancer cells. *J. Exp. Clin. Cancer Res.* **2019**, *38*, 71. https://doi.org/10.1186/s13046-019-1093-3.

216. Peng, J.; Zhou, J.; Sun, R.; Chen, Y.; Pan, D.; Wang, Q.; Chen, Y.; Gong, Z.; Du, Q. Dual-targeting of artesunate and chloroquine to tumor cells and tumor-associated macrophages by a biomimetic PLGA nanoparticle for colorectal cancer treatment. *Int. J. Biol. Macromol.* **2023**, *244*, 125163. https://doi.org/10.1016/j.ijbiomac.2023.125163.
217. Grasso, S.; Pereira, G.J.S.; Palmeira-Dos-Santos, C.; Calgarotto, A.K.; Martínez-Lacaci, I.; Ferragut, J.A.; Smaili, S.S.; Bincoletto, C. Autophagy regulates Selumetinib (AZD6244) induced-apoptosis in colorectal cancer cells. *Eur. J. Med. Chem.* **2016**, *122*, 611–618. https://doi.org/10.1016/j.ejmech.2016.06.043.
218. Ovejero-Sánchez, M.; González-Sarmiento, R.; Herrero, A.B. Synergistic effect of Chloroquine and Panobinostat in ovarian cancer through induction of DNA damage and inhibition of DNA repair. *Neoplasia* **2021**, *23*, 515–528. https://doi.org/10.1016/j.neo.2021.04.003.
219. Santiago-O'Farrill, J.M.; Weroha, S.J.; Hou, X.; Oberg, A.L.; Heinzen, E.P.; Maurer, M.J.; Pang, L.; Rask, P.; Amaravadi, R.K.; Becker, S.E.; et al. Poly(adenosine diphosphate ribose) polymerase inhibitors induce autophagy-mediated drug resistance in ovarian cancer cells, xenografts, and patient-derived xenograft models. *Cancer* **2020**, *126*, 894–907. https://doi.org/10.1002/cncr.32600.
220. Ovejero-Sánchez, M.; Rubio-Heras, J.; Vicente de la Peña, M.D.C.; San-Segundo, L.; Pérez-Losada, J.; González-Sarmiento, R.; Herrero, A.B. Chloroquine-Induced DNA Damage Synergizes with Nonhomologous End Joining Inhibition to Cause Ovarian Cancer Cell Cytotoxicity. *Int. J. Mol. Sci.* **2022**, *23*, 7518. https://doi.org/10.3390/ijms23147518.
221. Nunes, M.; Duarte, D.; Vale, N.; Ricardo, S. Pitavastatin and Ivermectin Enhance the Efficacy of Paclitaxel in Chemoresistant High-Grade Serous Carcinoma. *Cancers* **2022**, *14*, 4357. https://doi.org/10.3390/cancers14184357.
222. Gallardo, F.; Teiti, I.; Rochaix, P.; Demilly, E.; Jullien, D.; Mariamé, B.; Tilkin-Mariamé, A.F. Development and Potentiate Activity of Anti–BRAF V600 Inhibitors. *Clin. Skin Cancer* **2016**, *1*, 4–14.e3. https://doi.org/10.1016/j.clsc.2016.05.001.
223. Aloss, K.; Leroy Viana, P.H.; Bokhari, S.M.Z.; Giunashvili, N.; Schvarcz, C.A.; Bócsi, D.; Koós, Z.; Benyó, Z.; Hamar, P. Ivermectin Synergizes with Modulated Electro-hyperthermia and Improves Its Anticancer Effects in a Triple-Negative Breast Cancer Mouse Model. *ACS Pharmacol. Transl. Sci.* **2024**, *7*, 2496–2506. https://doi.org/10.1021/acsptsci.4c00314.
224. Luo, H.; Feng, Y.; Wang, F.; Lin, Z.; Huang, J.; Li, Q.; Wang, X.; Liu, X.; Zhai, X.; Gao, Q.; et al. Combinations of ivermectin with proteasome inhibitors induce synergistic lethality in multiple myeloma. *Cancer Lett.* **2023**, *565*, 216218. https://doi.org/10.1016/j.canlet.2023.216218.
225. Popović, K.J.; Popović, D.J.; Miljković, D.; Popović, J.K.; Lalošević, D.; Čapo, I. Co-treatment with nitroglycerin and metformin exhibits physicochemically and pathohistologically detectable anticancer effects on fibrosarcoma in hamsters. *Biomed. Pharmacother.* **2020**, *130*, 110510. https://doi.org/10.1016/j.biopha.2020.110510.
226. Popović, K.J.; Popović, D.J.; Miljković, D.; Popović, J.K.; Lalošević, D.; Poša, M.; Čapo, I. Disulfiram and metformin combination anticancer effect reversible partly by antioxidant nitroglycerin and completely by NF-κB activator mebendazole in hamster fibrosarcoma. *Biomed. Pharmacother.* **2021**, *143*, 112168. https://doi.org/10.1016/j.biopha.2021.112168.
227. Toffoli, G.; Doglioni, C.; Cernigoi, C.; Frustaci, S.; Perin, T.; Canal, B.; Boiocchi, M. P53 overexpression in human soft tissue sarcomas: Relation to biological aggressiveness. *Ann. Oncol.* **1994**, *5*, 167–172. https://doi.org/10.1093/oxfordjournals.annonc.a058771.
228. Hwang, H.J.; Nam, S.K.; Park, H.; Park, Y.; Koh, J.; Na, H.Y.; Kwak, Y.; Kim, W.H.; Lee, H.S. Prediction of TP53 mutations by p53 immunohistochemistry and their prognostic significance in gastric cancer. *J. Pathol. Transl. Med.* **2020**, *54*, 378–386. https://doi.org/10.4132/jptm.2020.06.01.
229. Das, P.; Kotilingam, D.; Korchin, B.; Liu, J.; Yu, D.; Lazar, A.J.; Pollock, R.E.; Lev, D. High prevalence of p53 exon 4 mutations in soft tissue sarcoma. *Cancer* **2007**, *109*, 2323–2333. https://doi.org/10.1002/cncr.22680.
230. Park, E.; Han, H.; Choi, S.E.; Park, H.; Woo, H.Y.; Jang, M.; Shim, H.S.; Hwang, S.; Kang, H.; Cho, N.H. p53 Immunohistochemistry and Mutation Types Mismatching in High-Grade Serous Ovarian Cancer. *Diagnostics* **2022**, *12*, 579. https://doi.org/10.3390/diagnostics12030579.
231. Flanagan, L.; Meyer, M.; Fay, J.; Curry, S.; Bacon, O.; Duessmann, H.; John, K.; Boland, K.C.; McNamara, D.A.; Kay, E.W.; et al. Low levels of Caspase-3 predict favourable response to 5FU-based chemotherapy in advanced colorectal cancer: Caspase-3 inhibition as a therapeutic approach. *Cell Death Dis.* **2016**, *7*, e2087. https://doi.org/10.1038/cddis.2016.7.
232. O'Donovan, N.; Crown, J.; Stunell, H.; Hill, A.D.; McDermott, E.; O'Higgins, N.; Duffy, M.J. Caspase 3 in breast cancer. *Clin. Cancer Res.* **2003**, *9*, 738–742.
233. Nakopoulou, L.; Alexandrou, P.; Stefanaki, K.; Panayotopoulou, E.; Lazaris, A.C.; Davaris, P.S. Immunohistochemical expression of caspase-3 as an adverse indicator of the clinical outcome in human breast cancer. *Pathobiology* **2001**, *69*, 266–273. https://doi.org/10.1159/000064337.

234. Nassar, A.; Lawson, D.; Cotsonis, G.; Cohen, C. Survivin and caspase-3 expression in breast cancer: Correlation with prognostic parameters, proliferation, angiogenesis, and outcome. *Appl. Immunohistochem. Mol. Morphol.* **2008**, *16*, 113–120. https://doi.org/10.1097/PAI.0b013e318032ea73.
235. Silva, F.F.V.E.; Padín-Iruegas, M.E.; Caponio, V.C.A.; Lorenzo-Pouso, A.I.; Saavedra-Nieves, P.; Chamorro-Petronacci, C.M.; Suaréz-Peñaranda, J.; Pérez-Sayáns, M. Caspase 3 and Cleaved Caspase 3 Expression in Tumorogenesis and Its Correlations with Prognosis in Head and Neck Cancer: A Systematic Review and Meta-Analysis. *Int. J. Mol. Sci.* **2022**, *23*, 11937. https://doi.org/10.3390/ijms231911937.
236. Rendic, S.; Guengerich, F.P. Metabolism and Interactions of Chloroquine and Hydroxychloroquine with Human Cytochrome P450 Enzymes and Drug Transporters. *Curr. Drug Metab.* **2020**, *21*, 1127–1135. https://doi.org/10.2174/1389200221999201208211537.
237. Rendic, S.P. Metabolism and interactions of Ivermectin with human cytochrome P450 enzymes and drug transporters, possible adverse and toxic effects. *Arch. Toxicol.* **2021**, *95*, 1535–1546. https://doi.org/10.1007/s00204-021-03025-z.
238. Vanachayangkul, P.; Kodchakorn, C.; Ta-Aksorn, W.; Im-Erbsin, R.; Tungtaeng, A.; Tipthara, P.; Tarning, J.; Lugo-Roman, L.A.; Wojnarski, M.; Vesely, B.A.; et al. Safety, pharmacokinetics, and potential neurological interactions of ivermectin, tafenoquine, and chloroquine in Rhesus macaques. *Antimicrob. Agents Chemother.* **2024**, *68*, e0018124. https://doi.org/10.1128/aac.00181-24.
239. Vanachayangkul, P.; Im-Erbsin, R.; Tungtaeng, A.; Kodchakorn, C.; Roth, A.; Adams, J.; Chaisatit, C.; Saingam, P.; Sciotti, R.J.; Reichard, G.A.; et al. Safety, Pharmacokinetics, and Activity of High-Dose Ivermectin and Chloroquine against the Liver Stage of Plasmodium cynomolgi Infection in Rhesus Macaques. *Antimicrob. Agents Chemother.* **2020**, *64*, e00741–20. https://doi.org/10.1128/AAC.00741-20.
240. Moore, B.R.; Page-Sharp, M.; Stoney, J.R.; Ilett, K.F.; Jago, J.D.; Batty, K.T. Pharmacokinetics, pharmacodynamics, and allometric scaling of chloroquine in a murine malaria model. *Antimicrob. Agents Chemother.* **2011**, *55*, 3899–3907. https://doi.org/10.1128/AAC.00067-11.
241. Mustapha, K.B.; Bakara-Odunola, B.T.; Magaji, G.; Obodozie-Ofoegbu, O.O.; Akumka, D.D. Pharmacokinetics of Chloroquine and Metronidazole in Rats. *J. Appl. Pharm. Sci.* **2015**, *5*, 090–094. https://doi.org/10.7324/JAPS.2015.50814.
242. de Vries, P.J.; Oosterhuis, B.; van Boxtel, C.J. Single-Dose Pharmacokinetics of Chloroquine and its Main Metabolite in Healthy Volunteers. *Drug Investig.* **1994**, *8*, 143–149. https://doi.org/10.1007/BF03259430.
243. Doeppner, T.R.; Coman, C.; Burdusel, D.; Ancuta, D.L.; Brockmeier, U.; Pirici, D.N.; Yaoyun, K.; Hermann, D.M.; Popa-Wagner, A. Long-term treatment with chloroquine increases lifespan in middle-aged male mice possibly via autophagy modulation, proteasome inhibition and glycogen metabolism. *Aging* **2022**, *14*, 4195–4210. https://doi.org/10.18632/aging.204069.
244. Mendes, A.M.; Albuquerque, I.S.; Machado, M.; Pissarra, J.; Meireles, P.; Prudêncio, M. Inhibition of Plasmodium Liver Infection by Ivermectin. *Antimicrob. Agents Chemother.* **2017**, *61*, e02005-16. https://doi.org/10.1128/AAC.02005-16.
245. Chiu, S.H.L.; Green, M.L.; Baylis, F.P.; Eline, D.; Rosegay, A.; Meriwether, H.; Jacob, T.A. Absorption, tissue distribution, and excretion of tritium-labeled ivermectin in cattle, sheep, and rat. *J. Agric. Food Chem.* **1990**, *38*, 2072–2078. https://doi.org/10.1021/jf00101a015.
246. Lo, P.K.; Fink, D.W.; Williams, J.B.; Blodinger, J. Pharmacokinetic studies of ivermectin: Effects of formulation. *Vet. Res. Commun.* **1985**, *9*, 251–268. https://doi.org/10.1007/BF02215150.
247. Chaccour, C.; Hammann, F.; Rabinovich, N.R. Ivermectin to reduce malaria transmission I. Pharmacokinetic and pharmacodynamic considerations regarding efficacy and safety. *Malar. J.* **2017**, *16*, 161. https://doi.org/10.1186/s12936-017-1801-4.
248. Juarez, M.; Schcolnik-Cabrera, A.; Dueñas-Gonzalez, A. The multitargeted drug ivermectin: From an antiparasitic agent to a repositioned cancer drug. *Am. J. Cancer Res.* **2018**, *8*, 317–331.
249. Rainsford, K.D.; Parke, A.L.; Clifford-Rashotte, M.; Kean, W.F. Therapy and pharmacological properties of hydroxychloroquine and chloroquine in treatment of systemic lupus erythematosus, rheumatoid arthritis and related diseases. *Inflammopharmacology* **2015**, *23*, 231–269. https://doi.org/10.1007/s10787-015-0239-y.
250. Schrezenmeier, E.; Dörner, T. Mechanisms of action of hydroxychloroquine and chloroquine: Implications for rheumatology. *Nat. Rev. Rheumatol.* **2020**, *16*, 155–166. https://doi.org/10.1038/s41584-020-0372-x.
251. Smit, M.R.; Ochomo, E.; Aljayyoussi, G.; Kwambai, T.; Abong’o, B.; Bayoh, N.; Gimnig, J.; Samuels, A.; Desai, M.; Phillips-Howard, P.A.; et al. Efficacy and Safety of High-Dose Ivermectin for Reducing Malaria Transmission (IVERMAL): Protocol for a Double-Blind, Randomized, Placebo-Controlled, Dose-Finding Trial in Western Kenya. *JMIR Res. Protoc.* **2016**, *5*, e213. https://doi.org/10.2196/resprot.6617.

252. Guzzo, C.A.; Furtek, C.I.; Porras, A.G.; Chen, C.; Tipping, R.; Clineschmidt, C.M.; Sciberras, D.G.; Hsieh, J.Y.; Lasseter, K.C. Safety, tolerability, and pharmacokinetics of escalating high doses of ivermectin in healthy adult subjects. *J. Clin. Pharmacol.* **2002**, *42*, 1122–1133. https://doi.org/10.1177/009127002401382731.
253. Duncan, D.; Rotunda, A.M. Injectable therapies for localized fat loss: State of the art. *Clin. Plast. Surg.* **2011**, *38*, 489–501. https://doi.org/10.1016/j.cps.2011.02.005.
254. Kast, R.E.; Alfieri, A.; Assi, H.I.; Burns, T.C.; Elyamany, A.M.; Gonzalez-Cao, M.; Karpel-Massler, G.; Marosi, C.; Salacz, M.E.; Sardi, I.; et al. MDACT: A New Principle of Adjunctive Cancer Treatment Using Combinations of Multiple Repurposed Drugs, with an Example Regimen. *Cancers* **2022**, *14*, 2563. https://doi.org/10.3390/cancers14102563.
255. Quesada, J.; Amato, R. The Molecular Biology of Soft-Tissue Sarcomas and Current Trends in Therapy. *Sarcoma* **2012**, *12*, 849456. https://doi.org/10.1155/2012/849456.
256. Anderson, J.L.; Denny, C.T.; Tap, W.D.; Federman, N. Pediatric sarcomas: Translating molecular pathogenesis of disease to novel therapeutic possibilities. *Pediatr. Res.* **2012**, *72*, 112–121. https://doi.org/10.1038/pr.2012.54.
257. Issaq, S.H.; Teicher, B.A.; Monks, A. Bioenergetic properties of human sarcoma cells help define sensitivity to metabolic inhibitors. *Cell Cycle* **2014**, *13*, 1152–1161. https://doi.org/10.4161/cc.28010.
258. Hoang, N.T.; Acevedo, L.A.; Mann, M.J.; Tolani, B. A review of soft-tissue sarcomas: Translation of biological advances into treatment measures. *Cancer Manag. Res.* **2018**, *10*, 1089–1114. https://doi.org/10.2147/CMAR.S159641.
259. Burningham, Z.; Hashibe, M.; Spector, L.; Schiffman, J.D. The epidemiology of sarcoma. *Clin. Sarcoma Res.* **2012**, *2*, 14. https://doi.org/10.1186/2045-3329-2-14.
260. Lakhter, A.J.; Sahu, R.P.; Sun, Y.; Kaufmann, W.K.; Androphy, E.J.; Travers, J.B.; Naidu, S.R. Chloroquine promotes apoptosis in melanoma cells by inhibiting BH3 domain-mediated PUMA degradation. *J. Investig. Dermatol.* **2013**, *133*, 2247–2254. https://doi.org/10.1038/jid.2013.56.
261. Molejon, M.I.; Swayden, M.; Fanale, D.; Bintz, J.; Gayet, O.; Soubeyran, P.; Iovanna, J. Chloroquine plays a cell-dependent role in the response to treatment of pancreatic adenocarcinoma. *Oncotarget* **2018**, *9*, 30837–30846. https://doi.org/10.18632/oncotarget.25745.
262. Masud Alam, M.; Kariya, R.; Kawaguchi, A.; Matsuda, K.; Kudo, E.; Okada, S. Inhibition of autophagy by chloroquine induces apoptosis in primary effusion lymphoma in vitro and in vivo through induction of endoplasmic reticulum stress. *Apoptosis* **2016**, *21*, 1191–1201. https://doi.org/10.1007/s10495-016-1277-7.
263. Folkerts, H.; Hilgendorf, S.; Wierenga, A.T.J.; Jaques, J.; Mulder, A.B.; Coffer, P.J.; Schuringa, J.J.; Vellenga, E. Inhibition of autophagy as a treatment strategy for p53 wild-type acute myeloid leukemia. *Cell Death Dis.* **2017**, *8*, e2927. https://doi.org/10.1038/cddis.2017.317.
264. Burikhanov, R.; Hebbar, N.; Noothi, S.K.; Shukla, N.; Sledziona, J.; Araujo, N.; Kudrimoti, M.; Wang, Q.J.; Watt, D.S.; Welch, D.R.; et al. Chloroquine-Inducible Par-4 Secretion Is Essential for Tumor Cell Apoptosis and Inhibition of Metastasis. *Cell Rep.* **2017**, *18*, 508–519. https://doi.org/10.1016/j.celrep.2016.12.051.
265. Weyerhäuser, P.; Kantelhardt, S.R.; Kim, E.L. Re-purposing Chloroquine for Glioblastoma: Potential Merits and Confounding Variables. *Front. Oncol.* **2018**, *8*, 335. https://doi.org/10.3389/fonc.2018.00335.
266. Alghamdi, H.A.; Al-Zharani, M.; Aljarba, N.H.; Alghamdi, A.A.; Alghamdi, A.A.; Aldahmash, B.A.; Elnagar, D.M.; Alkahtani, S. Efficacy of ivermectin against colon cancer induced by dimethylhydrazine in male wistar rats. *Saudi Pharm. J.* **2022**, *30*, 1273–1282. https://doi.org/10.1016/j.jsps.2022.06.024.
267. Sharmeen, S.; Skrtic, M.; Sukhai, M.A.; Hurren, R.; Gronda, M.; Wang, X.; Fonseca, S.B.; Sun, H.; Wood, T.E.; Ward, R.; et al. The antiparasitic agent ivermectin induces chloride-dependent membrane hyperpolarization and cell death in leukemia cells. *Blood* **2010**, *116*, 3593–3603. https://doi.org/10.1182/blood-2010-01-262675.
268. de Castro, C.G., Jr.; Gregianin, L.J.; Burger, J.A. Continuous high-dose ivermectin appears to be safe in patients with acute myelogenous leukemia and could inform clinical repurposing for COVID-19 infection. *Leuk. Lymphoma* **2020**, *61*, 2536–2537. https://doi.org/10.1080/10428194.2020.1786559.
269. Mohi-Ud-Din, R.; Chawla, A.; Sharma, P.; Mir, P.A.; Potoo, F.H.; Reiner, Ž.; Reiner, I.; Ateşşahin, D.A.; Sharifi-Rad, J.; Mir, R.H.; et al. Repurposing approved non-oncology drugs for cancer therapy: A comprehensive review of mechanisms, efficacy, and clinical prospects. *Eur. J. Med. Res.* **2023**, *28*, 345. https://doi.org/10.1186/s40001-023-01275-4.
270. Hijazi, M.A.; Gessner, A.; El-Najjar, N. Repurposing of Chronically Used Drugs in Cancer Therapy: A Chance to Grasp. *Cancers* **2023**, *15*, 3199. https://doi.org/10.3390/cancers15123199.
271. Stoker, M.; Macpherson, I. Syrian hamster fibroblast cell line BHK21 and its derivatives. *Nature* **1964**, *203*, 1355–1357. https://doi.org/10.1038/2031355a0.

272. Lalosević, D.; Stankov, S.; Lazarević-Ivanc, L.; Lalosević, V.; Knezević, I. Immunogenicity of BHK-rabies vaccine in human volunteers. *Med. Pregl.* **1998**, *51*, 17–19.
273. Shipman, C.; Vander Weide, G.C.; Ma, B.I. Prevalence of type R virus-like particles in clones of BHK-21 cells. *Virology* **1969**, *38*, 707–710. https://doi.org/10.1016/0042-6822(69)90192-5.
274. Walker, T.A.; Wilson, B.A.; Lewis, A.M.; Cook, J.L. E1A oncogene induction of cytolytic susceptibility eliminates sarcoma cell tumorigenicity. *Proc. Natl. Acad. Sci. USA* **1991**, *88*, 6491–6495. https://doi.org/10.1073/pnas.88.15.6491.
275. Mayo, J.; Lombardo, J.L.; Klein-Szanto, A.J.P.; Conti, C.J.; Moreira, J.L. An Oncogenic Virus Carried by Hamster Kidney Cells. *Cancer Res.* **1973**, *33*, 2273–2277.
276. Johnson, T.S.; Scholfield, C.I.; Parry, J.; Griffin, M. Induction of tissue transglutaminase by dexamethasone: Its correlation to receptor number and transglutaminase- mediated cell death in a series of malignant hamster fibrosarcomas. *Biochem. J.* **1998**, *331*, 105–112. https://doi.org/10.1042/bj3310105.
277. Traykovich, V. A Transplantable Hamster Sarcoma Produced by Spontaneously Transformed Kidney Cell Line of Baby Hamster. *J. Natl. Cancer Inst.* **1968**, *41*, 1039–1050. https://doi.org/10.1093/jnci/41.5.1039.
278. Marshall, R. Karyotype of the BHK 21 C13 cell line of the syrian hamster determined with the aid of quinacrine staining. *Chromosoma* **1972**, *37*, 395–404. https://doi.org/10.1007/BF00284888. PMID: 4115459.
279. Di Mayorca, G.; Greenblatt, M.; Trauthen, T.; Soller, A.; Giordano, R. Malignant transformation of BHK21 clone 13 cells in vitro by nitrosamines--a conditional state. *Proc. Natl. Acad. Sci. USA* **1973**, *70*, 46–49. https://doi.org/10.1073/pnas.70.1.46. PMID: 4346037; PMCID: PMC433180.
280. Shin, J.W.; Seol, I.C.; Son, C.G. Interpretation of Animal Dose and Human Equivalent Dose for Drug Development. *J. Korean Med.* **2010**, *31*, 1–7.
281. Nair, A.B.; Jacob, S. A simple practice guide for dose conversion between animals and human. *J. Basic Clin. Pharm.* **2016**, *7*, 27–31. https://doi.org/10.4103/0976-0105.177703.
282. Blanchard, O.L.; Smoliga, J.M. Translating dosages from animal models to human clinical trials—Revisiting body surface area scaling. *FASEB J.* **2015**, *29*, 1629–1634. https://doi.org/10.1096/fj.14-269043.
283. LaRusso, N.F.; Szczepanik, P.A.; Hofmann, A.F. Effect of deoxycholic acid ingestion on bile acid metabolism and biliary lipid secretion in normal subjects. *Gastroenterology* **1977**, *72*, 132–140.
284. Liu, L.; Dong, W.; Wang, S.; Zhang, Y.; Liu, T.; Xie, R.; Wang, B.; Cao, H. Deoxycholic acid disrupts the intestinal mucosal barrier and promotes intestinal tumorigenesis. *Food Funct.* **2018**, *9*, 5588–5597. https://doi.org/10.1039/C8FO01143E.
285. Ju, J.; Zhang, C.; Yang, J.; Yang, Q.; Yin, P.; Sun, X. Deoxycholic acid exacerbates intestinal inflammation by modulating interleukin-1β expression and tuft cell proportion in dextran sulfate sodium-induced murine colitis. *PeerJ* **2023**, *11*, e14842. https://doi.org/10.7717/peerj.14842.
286. Sousa, A.T.O.; de Vasconcelos, J.M.B.; Soares, M.J.G.O. Software Image Tool 3.0 as an Instrument for Measuring Wounds. *J. Nurs. UFPE On Line* **2012**, *6*, 2569–2573. Available online: https://scispace.com/pdf/software-image-tool-3-0-as-an-instrument-for-measuring-4qym1lz30p.pdf. .
287. Scudiero, D.A.; Shoemaker, R.H.; Paull, K.D.; Monks, A.; Tierney, S.; Nofziger, T.H.; Currens, M.J.; Seniff, D.; Boyd, M.R. Evaluation of a soluble tetrazolium/formazan assay for cell growth and drug sensitivity in culture using human and other tumor cell lines. *Cancer Res.* **1988**, *48*, 4827–4833.

Disclaimer/Publisher's Note: The statements, opinions and data contained in all publications are solely those of the individual author(s) and contributor(s) and not of MDPI and/or the editor(s). MDPI and/or the editor(s) disclaim responsibility for any injury to people or property resulting from any ideas, methods, instructions or products referred to in the content.
